# Response of winter wheat genotypes to salinity stress under controlled environments

**DOI:** 10.3389/fpls.2024.1396498

**Published:** 2024-06-24

**Authors:** Amal Ehtaiwesh, V. S. John Sunoj, Maduraimuthu Djanaguiraman, P. V. Vara Prasad

**Affiliations:** ^1^ Department of Agronomy, Crop Physiology Lab, 2004 Throckmorton Plant Science Center, Kansas State University, Manhattan, KS, United States; ^2^ Crop Eco-physiology, Texas A&M AgriLife Research and Extension Center, Uvalde, TX, United States; ^3^ Department of Crop Physiology, Tamil Nadu Agricultural University, Coimbatore, India

**Keywords:** salinity, germination, early seedling stage, booting stage, yield, wheat

## Abstract

This study was conducted in controlled environmental conditions to systematically evaluate multi-traits responses of winter wheat (*Triticum aestivum* L.) genotypes to different salinity levels. Responses were assessed at the germination to early seedling stage (Experiment 1). Seeds of different genotypes (n=292) were subjected to three salinity levels (0 [control], 60, and 120 mM NaCl). Principal Component Analysis (PCA) revealed that among studied traits seedling vigor index (SVI) contributed more towards the diverse response of genotypes to salinity stress. Based on SVI, eight contrasting genotypes assumed to be tolerant (Gage, Guymon, MTS0531, and Tascosa) and susceptible (CO04W320, Carson, TX04M410211) were selected for further physio-biochemical evaluation at the booting stage (Experiment 2) and to monitor grain yield. Higher level of salinity (120 mM NaCl) exposure at the booting stage increased thylakoid membrane damage, lipid peroxidation, sugars, proline, and protein while decreasing photosynthesis, chlorophyll index, starch, and grain yield. Based on grain yield, the assumed magnitude of the genotypic response shown in Experiment 1 was not analogous in Experiment 2. This indicates the necessity of individual screening of genotypes at different sensitive growth stages for identifying true salinity-tolerant and susceptible genotypes at a particular growth stage. However, based on higher grain yield and its least percentage reduction under higher salinity, Guymon and TX04M410211 were identified as tolerant, and Gage and CO04W320 as susceptible at the booting stage, and their biparental population can be used to identify genomic regions for booting stage-specific salinity response.

## 1 Introduction

Increases in soil and water salinity negatively impact growth, development, and yield of different crops around the globe (El Sabagh et al., 2021; Saddiq et al., 2021[wheat; *Triticum aestivum* L.]; Ali et al., 2021; Khan et al., 2022 [rice; *Oryza sativa* L.]; Zahra et al., 2020; Zakavi et al., 2022 [maize; *Zea mays* L.]; Ehtaiwesh, 2022 [barley *Hordeum vulgare* L]; Mulaudzi et al., 2022; Sun et al., 2023 [sorghum *Sorghum bicolor* (L.) Moench]) and is a risk to food security (Fatima et al., 2020; Mukhopadhyay et al., 2021). It is projected that over 800 million hectares of land will be affected by salinity in the near future. At present, 20% of the world’s irrigated land is salt-affected and/or irrigated with saline water, and every year, two million additional hectares of cropping land are affected by salinity (Rengasamy, 2006; Tuteja, 2007; FAO, 2008; Qadir et al., 2008).

Additionally, because of the increase in the salinity of agricultural land, it is predicted that 50% of agricultural land will become barren by the middle of the 21^st^ century and it will be more critical in the areas with low rainfall and near coastal regions (Mustafa et al., 2019; Hopmans et al., 2021). Conversely, salt can also accumulate in regions with adequate rainfall as a result of poor drainage of soils and substantial use of fertilizers that make the land unsuitable for crop production (Plaut et al., 2013). These trends and future demographic projections propose the importance of identifying salinity-tolerant crops and their diverse genotypes with the potential to withstand salinity and/or develop salinity-tolerant genotypes of important crops that can be cultivated for effective utilization of salt-affected land and saline water resources.

Wheat plays a crucial role in human nutrition and food security by providing 19% of the daily calories and 21% of protein requirements (Tadesse et al., 2019). Wheat occupies 30% of world cereal production with 808 million tons from 219 million hectares (FAOSTAT, 2022), and the demand for wheat is expected to increase in the future to feed the uncontrollably and rapidly growing global population (UN, 2022). Therefore, improvement in wheat yield by exploitation of underutilized land for wheat cultivation will be one of the strategies to meet increasing demand. Unfortunately, the major limitation is that the majority of underutilized lands are salt-affected regions.

Wheat is normally grown under irrigated, dryland, and rain-fed conditions and it has a moderate potential to tolerate salinity (Acevedo et al., 2002; Dubcovsky and Dvora, 2007; Paul et al., 2019). Still, salinity alters the morphological, physiological, biochemical, and agronomic traits of wheat leading to a reduction in total grain yield (Yassin et al., 2019; Zeeshan et al., 2020; Saddiq et al., 2021; Seleiman et al., 2022). Francois et al. (1986) found that a salinity level of >4.5 dSm^-1^ electrical conductivity (EC) reduces the percentage of wheat plant establishment per unit area and an 8.8 dSm^-1^ salinity level reduces 50% of plant emergence. Thus, salinity is considered one of the chief challenges of wheat productivity. Additionally, salinity negatively affects plant growth by reducing seed germination (Sairam et al., 2002), seedling characters (Ehtaiwesh, 2019), root/shoot length, total dry matter accumulation (Datta et al., 2009), number of spikelet in the main spike of wheat (Tabatabaee et al., 2023) and cell division and development of shoot and root (Ragaey et al., 2022). Salinity also reduces of number of leaves in the main shoot of wheat which results in lesser leaf area and that in turn leads to the reduction in whole plant carbon assimilation (El Sabagh et al., 2021).

Besides, salinity increases the reactive oxygen species (ROS) production and oxidative stress and reduces enzymatic or nonenzymatic ROS quenching activities, as a result, it causes mild to severe oxidative damage in cellular and membrane components such as DNA, proteins, and lipids (Zhang et al., 2022; Sadak and Dawood, 2023). The salinity creates an imbalance in osmotic regulation that prevents or reduces water uptake, and Na and Cl ions toxicity are the identified explanations related to the adverse effect of salinity on germination and seedling growth (Munns et al., 2006; Taha and Abd El-Samad, 2022). Furthermore, salinity affects the balance of primary metabolites such as total soluble sugars, reduced sugars, nonreducing sugars, starch, lipids, and proteins (De Santis et al., 2021; Hussain et al., 2021; Kesh et al., 2022; Masarmi et al., 2023; Sadak and Dawood, 2023).

Alterations in morphological and physio-biochemical mechanisms under hostile situations created by salinity generate serious negative impacts on crop yield resulting in crop loss. For instance, Hasan et al. (2015) conducted a study in a salinity level of 12 dSm^−1^ significantly reduced grains per spike, grain weight, and seed yield in both tolerant and sensitive wheat cultivars with considerable changes in physio-biochemical traits. There are a few field studies that imply the importance of mitigating salinity to improve wheat production in salt-affected regions. A field study from Libya on hard wheat (*Triticum durum* desf) revealed that spike number, grain number, grain yield, and harvest index were substantially reduced due to salinity (Ehtaiwesh and Rashed, 2020). In another field study from Egypt, Mansour et al. (2020) reported that an increase in salinity levels of irrigation water resulted in a significant reduction in grain yield and other agronomic traits. Hence, identifying and cultivating wheat genotypes that demonstrate tolerance to salinity is one of the best strategies to limit and/or overcome the reduction of yield which is also a reinforcement to confront present and future negative impacts of salinity on wheat production. Additionally, identifying contrasting genetic pools of diversely responding genotypes to salinity can be exploited for developing high-yielding and salt-tolerant genotypes is a potential mode to increase or maintain the production in salt-affected agroclimatic regions and/or to introduce wheat cultivation in salt-affected underutilized regions.

To the best of our knowledge, till now the screening to scale the multi-traits responses of large germplasm collections of winter wheat genotypes to salinity at highly sensitive growth stages such as seed germination and early seedling stage to exploit the rich genetic resource for the breeding program and salinity management are inadequate and limited. Therefore, this study was conducted with the following specific objectives; (1) to screen the multi-trait responses of different genotypes of winter wheat at seed germination to early seedling stage exposed to different salinity levels to identify and select contrastingly responding genotypes and (2) to further monitor the important physiological and biochemical traits of selected contrasting winter wheat genotypes after the exposure of higher salinity level at booting stage and validate its salinity tolerance and susceptibility based on agronomic traits. We hypothesize that the identified magnitude of genotypic tolerance and susceptibility from the multi-trait responses at germination to the early seedling stage are persistent in successive growth stages. And the most responsive traits at germination to the early seedling stage can be engaged as indicators and selection criteria for identifying salinity tolerance.

## 2 Materials and methods

### 2.1 Experiment 1

#### 2.1.1 Plant materials, salinity treatments, and growth conditions

Experiment 1 was conducted at the Department of Agronomy, Kansas State University, Manhattan, KS, USA to evaluate the multi-trait responses of different winter wheat genotypes at the germination to early seedling stage. The Hard Winter Wheat Association Mapping Panel (HWWAMP) developed by the Triticeae Coordinated Agricultural Project (TCAP; U.S. Department of Agriculture [USDA) and National Institute of Food and Agriculture [NIFA]; USA) was used for experiment 1.

To begin experiment 1, healthy seeds of 292 winter wheat genotypes (**Supplementary Table 1**) from the germplasm were surface sterilized with sodium hypochlorite solution (5%; NaOCl) for five minutes, washed with distilled water, and air dried. Two different concentrations of saline solutions were prepared using deionized water (60 and 120 mM sodium chloride [NaCl] with electric conductivity [EC] values of 7.5 and 14.5 dSm^-1^, respectively), and deionized water was used as a control solution (0 mM NaCl). To study the germination traits, three sets of 20 seeds from each genotype were placed in a petri dish lined with Whatman No. 1 filter paper disc. To this, different saline (5 mL; 60, and 120 mM NaCl) and control (5 mL; 0 mM NaCl) solutions were added which were counted as salinity and absolute control treatments, respectively. A total of five replications were used per genotype per control and salinity treatments. After that, all the petri dishes were placed in the dark for 8 days (8 days after sowing [DAS]; germination period) at 20 ± 2°C in a refrigerated incubator (Thermo Precision Model 818; Thermo Fisher Scientific Inc., USA). Filter paper discs were moisturized in the same manner on a daily basis till the end of the germination period and discs were changed once every two days to prevent salt accumulation due to evaporation. The following germination traits were recorded during the germination period.

#### 2.1.2 Germination traits

The seeds were considered germinated (Feekes 0.9) when both plumule and radicle were extended more than 2 mm from the seed (Islam et al., 2012).

The germination percentage (G %) was calculated according to Nasri et al., 2011. The following formula was used to calculate G %.


(1) G % =(NSG÷TNSS)×100 

where, NSG is the number of seeds germinated at the end of the germination period and TNSS is the total number of seeds sown.

The germination index (GI %) of seeds in the salinity treatments was calculated according to Karim et al. (1992). The following formula was used to calculate GI %.


(2) GI %=(% GNaCl÷% GC)×100

where, % GNaCl is the percentage of seed germination at different salinity treatments. % GC is the percentage of seed germination in the control treatment.

The germination rate (GR) was calculated according to Rubio-Casal et al. (2003). The number of germinated seeds was recorded at a 24-hour interval from sowing till the end of the germination period to calculate GR. The following formula was used to calculate GR.


(3) GR=(n1t1)+ (n2t2)+…+ (nxtx)÷TNGS

where, n_1_ is the number of seeds germinated on the first day of germination, t_1_ is the number of days taken for first germination and TNGS is the total number of seeds germinated.

Mean daily germination (MDG) was calculated according to Gairola et al. (2011). The following formula was used to calculate MDG.


(4) MDG=TNGS÷TNDG

where, TNDG is the total number of days taken for final germination.

#### 2.1.3 Early seedling traits

At the early seedling stage (Feekes 1.0; 9 DAS), morphological traits (shoot and root length and dry weight) were measured from five representative uniform seedlings from each replication. Selected seedlings were dissected and shoot and root lengths were recorded. The lengths from the seed to the tip of the root and leaf blade were measured using a digital vernier caliper and were recorded as root length and shoot length, respectively. The fresh weights of the shoot and root were recorded using a digital weighing balance (Salter Brecknell, ESA-600, Florida, USA) and then dried in a hot air oven at 70°C till they attained stable weight. After that, shoot and root dry weights were recorded. Using the morphological traits, the salinity tolerance index (STI) and seedling vigor index (SVI) were calculated.

The following formula was used to calculate STI (Tsegay and Gebreslassie, 2014):


(5) STI=(SdwNaCl÷SdwC)×100

where, SdwNaCl is the dry weight of the seedling from salinity treatments and SdwC is the dry weight of the seedling from the control treatment.

The following formula was used to calculate SVI (Abdoli et al., 2013):


(6) SVI=(SL×G%)100

where, SL is the seedling length and G% is the germination percentage.

#### 2.1.4 Statistical analysis

The statistical design of experiment 1 was a factorial complete randomized block with five replications per genotype and treatment. The recorded germination and early seedling stage traits were subjected to Analysis of Variance (ANOVA) using the generalized linear model (GLM) procedure in SAS 9.4 (SAS Institute Inc., Cary, NC, USA). Salinity levels, genotypes, and their interactions were used as independent factors. The means were compared using *post-hoc* Duncan’s multiple range test (DMRT) using SPSS (SPSS Inc. Ver.16, Chicago, USA).

#### 2.1.5 Principal component analysis

Principal Component Analysis (PCA) was conducted to identify the traits that contribute to the differential response of genotypes to different salinity levels. Principal Component Analysis (PCA) was performed using software XLstat ver. 2014.5.

### 2.2 Experiment 2

#### 2.2.1 Plant materials and husbandry, salinity treatments, and growth conditions

Experiment 2 was conducted in controlled environment facilities at the Department of Agronomy, Kansas State University, Manhattan, KS, USA. From experiment 1, eight genotypes were selected and classified into two categories based on their contrasting responses to different salinity levels (Gage, Guymon, MTS0531, and Tascosa [tolerant] and CO04W320, Carson, TX04M410211 and 2174–05 [susceptible], details of the selection of genotype are in the result section). These genotypes were used for further evaluation of responses of physiological and biochemical traits after exposure to higher salinity level (120 mM NaCl [EC=14.5 dSm^-1^]) at the booting stage (Feekes 10.0) and to monitor the agronomic traits.

Seeds of selected winter wheat genotypes were sown at a depth of 3 cm in trays containing Sunshine Metro Mix 360 growing medium (Hummert International, Topeka, KS, USA). Eight days after germination (DAG), seedlings were vernalized for 56 days at 4°C with 8 hours of photoperiod. After the vernalization, seedlings were transplanted into pots (3 seedlings/genotype/pot [1.6 L; length 24 cm x width 10 cm]), filled with Sunshine Metro Mix 360 growing medium. A total of five replications were maintained for each genotype and control salinity treatments. Two days after transplanting (DAT), seedlings were fertilized with liquid iron (Iron 5%; Bonide products, Oriskany, NY, USA), 35 g and 4 g of Osmocote classic controlled release plant nutrients (14:14:14 NPK), and Micro max micronutrient granules (Hummert International, Topeka, KS, USA), respectively.

Subsequently, pots were transferred and maintained in growth chambers (Conviron Model CMP 3244, Winnipeg, MB, Canada) with set optimum growth conditions viz., chamber temperature of 25/15°C (day/night; mean daily temperature of 20°C; Maswada et al., 2021), relative humidity (RH) of 70% and photoperiod of 16 hours with a light intensity of 800 µmol m^2^ s^1^ photosynthetically active radiation (PAR) at the plant canopy level using cool fluorescent lamps. A periodic transition from maximum day to minimum night temperature and vice versa within a time span of 4 hours was followed to replicate the diurnal temperature fluctuation under natural field conditions. The air temperature inside the growth chambers was monitored at 10-minute intervals throughout the experiment using HOBO data logger (Onset UTBi-001; TidbiT v2 Temperature logger; Bourne, MA, USA). Plants were watered on a daily basis to avoid water stress and the position of pots was changed randomly at 7-day intervals to avoid positional effects. The light intensity on the canopy was measured by using a light sensor reader and 6 sensor quantum bars (field scout and light scout; Spectrum Technologies, Inc., Aurora, IL, USA) and maintained constant light intensity on the canopy according to the growth of plants by adjusting the fluorescent lamps towards the roof of the growth chambers. After proper establishment of plants, seedlings were thinned to two plants per pot and a systemic insecticide, Marathon (1.5 g; 1% Imidacloprid: 1–([6–Chloro–3–pyridinyl] methyl–N–nitro–2–imidazolidinimine; OHP Inc, Maryland, PA, USA) was applied to each pot to avoid infestation of sucking insect pests. At the onset of the booting stages (Feekes 10.0), plants of each genotype were divided into two groups, one set was treated with a high concentration of a saline solution (120 mM NaCl [14.5 dSm^-1^]), and the other was irrigated with deionized water (0 mM NaCl) counted as salinity and absolute control treatments, respectively. After 10 days of salinity imposition at the booting stage, seedlings were irrigated with deionized water and maintained at optimum growth temperature (25/15°C day/night) till they attained physiological maturity. The reason behind selecting the booting stage is that most cereal crops are sensitive to abiotic stress during booting stage, because at this stage, the microgametogenesis and macrogametogenesis processes occur. Compared to the heading or anthesis stage, booting stage is highly sensitive to salinity (He et al., 2019).

Another independent experiment with the same genotypes, growing conditions, growth stage, and stress for the same period was repeated, and the same set of physiological, biochemical, growth, and yield traits were recorded to validate the results and check the repeatability. A total of two different growth chambers were used during each independent experiment to expose plants to high salinity levels as mentioned above.

#### 2.2.2 Physiological traits

##### 2.2.2.1 Leaf photosynthetic rate, chlorophyll fluorescence, and chlorophyll index

At the booting stage, the main stem of all the plants was tagged for recording leaf photosynthesis, chlorophyll fluorescence, and chlorophyll index. The above measurements were taken from the same flag leaves of three tagged plants of each genotype after 10 days of imposition of salinity. The leaf photosynthetic rate (*P*_N_) and stomatal conductance (*g*_s_) were measured between 1000 and 1100 h from fully expanded flag leaves of tagged plants using a portable photosynthesis system (LI-6400 XT; LICOR, USA). The CO_2_ concentration in the leaf chamber of the portable photosynthesis system was set to 400 µmol mol^-1^ and the block temperature was adjusted to the set daytime maximum temperature (25°C). The internal light source (red-blue light-emitting diode [LED]) in the portable photosynthesis system was set to supply a light intensity of 1000 µmol m^-2^ s^-1^ photosynthetically active radiation (PAR). The intrinsic water use efficiency (iWUE) was calculated from the ratio of *P*_N_ to *g*_s_ (Rymbai et al., 2014).

The chlorophyll fluorescence was measured to estimate the maximum quantum yield of PS II (Fv/Fm) and thylakoid membrane damage (F0/Fm) from flag leaves after 60 min of dark adaptation using chlorophyll fluorometer (OS30p+; OptiSciences, USA) equipped with light pulse intensity of 3000 mmol m^-2^ s^-1^ and pulse duration of three seconds. The chlorophyll index was measured using a chlorophyll meter (SPAD-502 Plus; Konica Minolta Inc., Japan).

#### 2.2.3 Biochemical traits

Leaf tissues for analyzing biochemical traits were collected from the middle portion of the flag leaves (without midrib) on which physiological traits were recorded. The collected leaf tissue samples were stored in vials, immersed in liquid nitrogen, and kept at -80°C until further analysis of the following biochemical traits.

##### 2.2.3.1 Sugars and starch

Sugars were extracted from frozen leaf tissue (0.2 g) using ethanol (70%). The tissue was ground into powder using liquid nitrogen, homogenized thoroughly with ethanol (70%), incubated at 70°C in a water bath for 30 min, and filtered through Whatman No 1 filter paper. The filtrate was used for the estimation of total soluble sugars (Dubois et al., 1956) and reducing sugars (Somogyi, 1952). The difference between total sugars and reducing sugars was considered as non-reducing sugars (Malhotra and Sarkar, 1979; Sunoj et al., 2016). Starch was estimated by following the methods of Hedge and Hofreiter (1962).

##### 2.2.3.2 Protein, proline, and lipid peroxidation

Total protein was extracted and estimated by following Sunoj et al. (2014) and Bradford (1976), respectively. Free proline was quantified according to the method of Bates et al. (1973). Lipid peroxidation was measured in terms of malondialdehyde content (MDA, extinction coefficient (ε) = 155 mmol^-1^ cm^-1^), a product of lipid peroxidation, following the method of Heath and Packer (1968).

#### 2.2.4 Agronomic traits

At maturity, five plants per genotype from control and salinity treatments were hand-harvested by cutting them from the base at the soil level. The harvested plants were separated into different parts (i.e., leaves, stems, main spike, and other spikes) and dried in a hot air oven at 40°C for 10 days. The total dry matter accumulation was calculated after the plant parts reached constant dry weight. After that, the number of spikelet per plant was counted from the spikes, hand-threshed to separate grains, and the grain number per spike was manually counted. After that, the total grain yield from the spike per plant and individual grain weight were calculated. The harvest index (HI) was calculated as the ratio of total grain weight per plant to total dry matter accumulated (including the grains).

#### 2.2.5 Statistical analysis

The statistical design of experiment 2 was a split-plot design with five replications (one pot with three plants considered as one replication). Salinity was the main plot factor and genotypes were assigned to sub-plots. Two levels of treatments were control (0 mM NaCl) and salinity (120 mM NaCl), and the genotype had eight levels selected from experiment 1. The recorded physiological, biochemical, and agronomical traits were subjected to Analysis of Variance (ANOVA) using the generalized linear model (GLM) procedure in SAS 9.4 (SAS Institute Inc., Cary, NC, USA). The means were compared using *post-hoc* Duncan’s multiple range test (DMRT) using SPSS (SPSS Inc. Ver.16, Chicago, USA).

## 3 Results

### 3.1 Experiment 1

Significant responses (*P*<0.01) were observed in all the studied germination and early seedling traits when treated with different salinity levels (0, 60, and 120 mM NaCl), in which five out of ten traits are highly significant (*P*<0.001) across genotypes, salinity levels, and their interaction. Shoot length, root length, seedling length, seedling dry weight, and salt tolerance index (STI) were the traits that showed highly significant differences (**Table 1**). The effect of salinity levels decreased all the studied traits across genotypes as compared to the control treatment (0 mM NaCl), except the germination rate (GR). At the same time, the magnitude of the above changes in traits varied across the genotypes and different salinity levels (**Table 2**; **Supplementary Tables 2, 3**).

**Table 1:** Probability values of effects of salinity (S), genotype (G), and interaction (S x G) on different winter wheat genotypes (n*=*292) at germination (Feekes 0.9) to early seedling (Feekes 1.0) stages (experiment 1).

	Variables		
Traits	Salinity (S)	Genotype (G)	S x G
Germination traits			
Germination percentage (G%)	<0.01	0.456	0.498
Germination index (GI; %)	<0.01	0.520	0.554
Mean daily germination (MDG)	<0.01	0.269	0.432
Germination rate (GR; day ^-1^)	<0.01	0.349	0.416
Early seedling traits			
Shoot length (cm)	< 0.001	< 0.001	< 0.001
Root length (cm)	< 0.001	< 0.001	< 0.001
Seedling length (cm)	< 0.001	< 0.001	< 0.001
Seedling dry weight (g)	< 0.001	< 0.001	< 0.001
Salt tolerance index (STI)	< 0.001	< 0.001	< 0.001
Seedling vigor index (SVI)	<0.01	0.235	0.374

**Table 2:** Variations in mean and range of variations across different winter wheat genotypes (n*=*292) in germination and early seedling traits at germination (Feekes 0.9) and early seedling (Feekes 1.0) stages exposed to control treatment (0 mM NaCl) and different levels of salinity (60, and 120 mM NaCl) (experiment 1).

Traits	Salinity levels (mM NaCl)			Range of variation and % change	
	0	60	120	(60 mM NaCl)	(120 mM NaCl)
Germination traits					
Germination percent (G%)	98^a*^	92^b^	60^c^	77.5 – 98.7^Ψ^ (-21.5/0.0)^¥^	28.8 - 87.5 (-70.9/-11.4)
Germination index (GI; %)	100^a^	94^a^	60^b^	78.5 - 100 (-21.4/0.5)	29.1 - 88.7 (-70.9/-11.3)
Mean daily germination (MDG)	7^a^	4^b^	2^c^	3.2 - 5.6 (-54.4/-28.8)	1.1 - 4.8 (-84.9/-41.9)
Germination rate (GR; d ^-1^)	2^c^	3^b^	4^a^	2.4 - 3.3 (17.4/52.4)	2.5 - 4.5 (22.6/117.2)
Early seedling traits					
Shoot length (cm)	8^a^	5^b^	2^c^	4 - 6.9 (-45.8/-16.0)	1.9 - 5.6 (-76.2/-32.5)
Root length (cm)	7^a^	4^b^	2^c^	3.2 - 5.7 (-52.1/-15.1)	0.9 - 4.0 (-86.6/-43.2)
Seedling length (cm)	14^a^	9^b^	4^c^	7.2 - 12.7 (-48.8/-16.9)	2.8 - 9.7 (-80.9/-37.5)
Seedling dry weight (g)	0.06^a^	0.04^b^	0.02^c^	0.03–0.05 (-50.9/-17.1)	0.012- 0.03 (-78.7/-46.9)
Salt tolerance index (STI)	100^a^	63^b^	33^c^	49.3 - 83.6 (-50.7/-16.4)	22.5 - 52.8 (-77.4/-47.2)
Seedling vigor index (SVI)	14^a^	9^b^	2^c^	6.7 - 12.2 (-50.7/-19.1)	1.00 - 8.4 (-92.5/-45.4)

*Individual value is the mean of different winter wheat genotypes (n=292) under different salinity levels 946 (0, 60, and 120 mM NaCl). Values followed by different letters are significantly different according to 947 Duncan’s multiple range test (DMRT; P < 0.05). ^Ψ^Highest and lowest values of a trait at different salinity 948 levels (60 and 120 mM NaCl). ^¥^Values in parenthesis are the range of least and highest relative change 949 (%) of a trait from the control treatment (0 mM NaCl) to salinity levels (60 and 120 mM NaCl). (-) 950 indicates percentage reduction and (+) indicates percentage increase.

#### 3.1.1 Traits contributing to the differential response of genotypes to salinity

**Figure 1:**
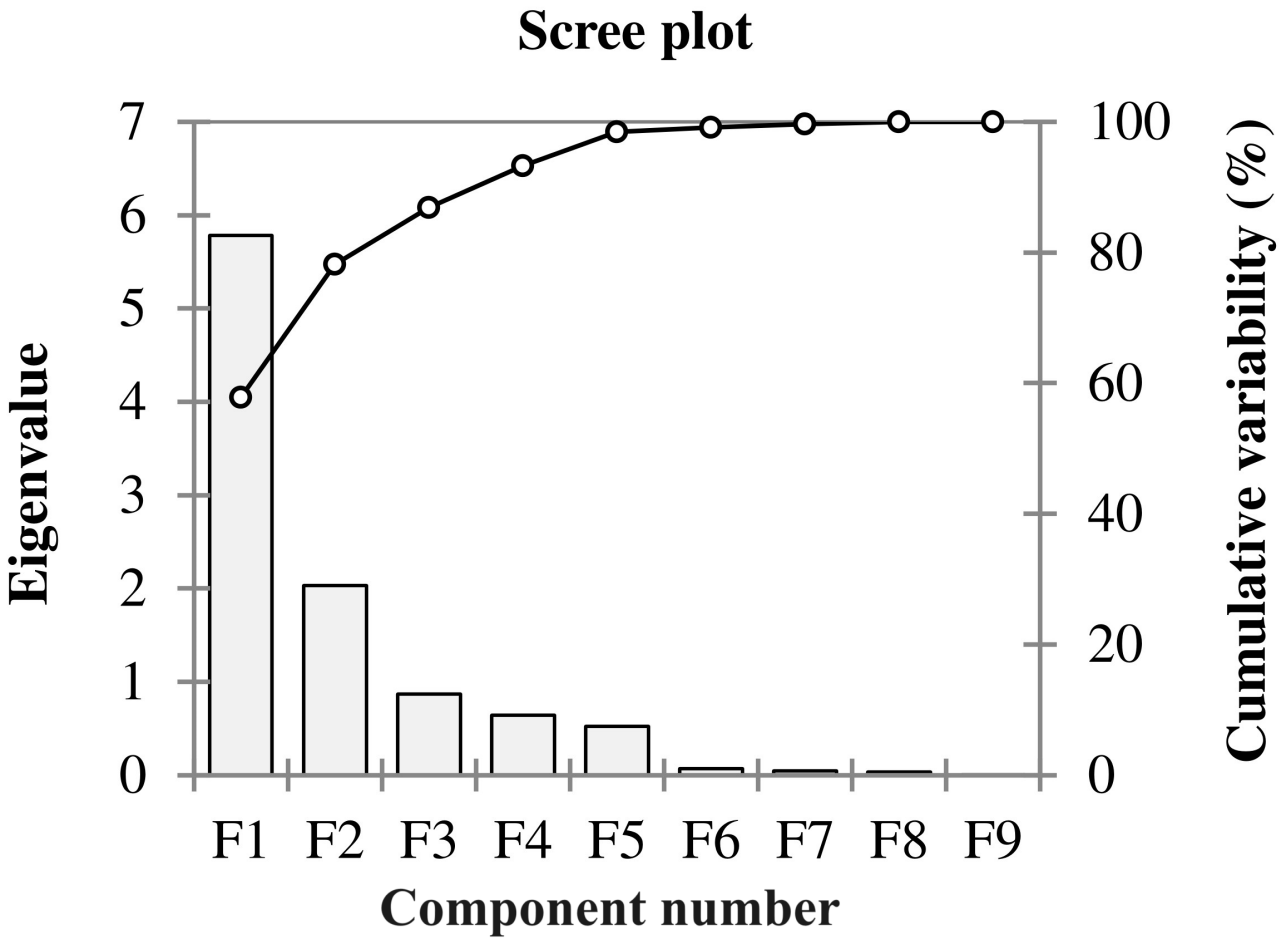
Scree plot showing eigenvalues in response to the number of components for the estimated variables of different winter wheat genotypes (n*=*292) in experiment 1 at germination (Feekes 0.9) and early seedling (Feekes 1.0) stages.

**Figure 2:**
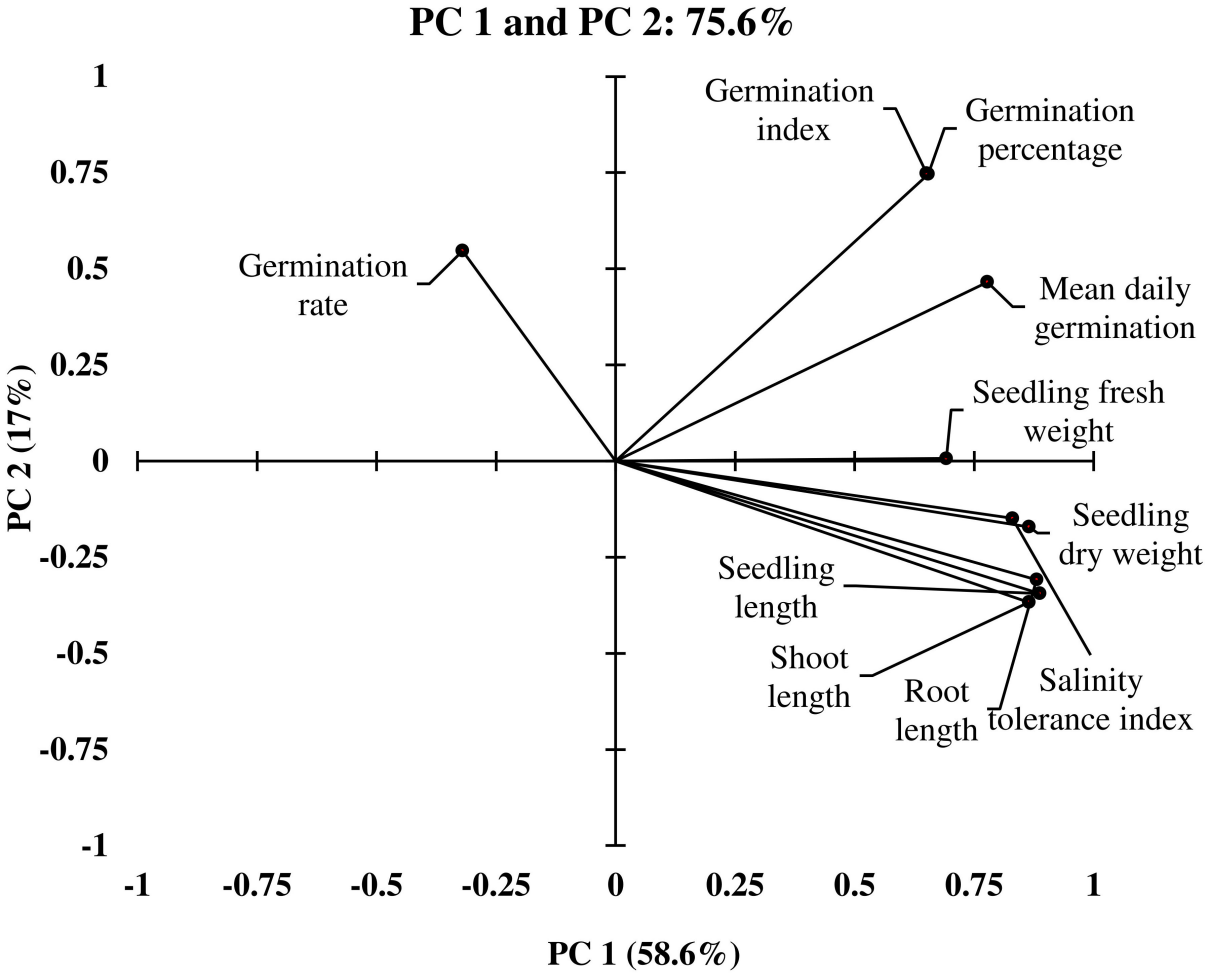
Loading plot of Principle Component Analysis (PCA) illustrating the distribution of germination and early seedling traits measured from different winter wheat genotypes (n*=*292) in experiment 1 at germination (Feekes 0.9) and early seedling (Feekes 1.0) stages.

The Principal Component Analysis (PCA) was conducted to identify the important germination and early seedling traits that contribute more towards the differential response of genotypes to salinity. These could be indicators of and/or responsible for the salinity tolerance in genotypes. The outputs from PCA were used to select the extremely contrasting genotypes for further evaluation in experiment 2. Out of ten principal components, the first two principal components (PC1 and PC2) of PCA comprise 75.62% (PC1 [58.68%] and PC2 [16.93%]) of cumulative variance of the total variance with an eigenvalue of 6.4 and 1.7, respectively (**Table 3**, **Figures 1**, **2**).

**Table 3:** Principal component analysis (PCA) and contribution (%) for germination and early seedling traits of different winter wheat genotypes (n*=*292) at germination (Feekes 0.9) and early seedling (Feekes 1.0) stages (experiment 1).

	PC1	PC2
Eigenvalue	6.46	1.86
Variability (%)	58.68	16.93
Cumulative (%)	58.68	75.62
	Contribution of traits (%)	
Germination traits		
Germination percentage (G%)	6.10	31.67
Germination index (GI; %)	5.81	31.90
Mean daily germination (MDG)	10.22	2.345
Germination rate (GR; d^-1^)	3.72	9.78
Early seedling traits		
Shoot length (cm)	11.26	7.09
Root length (cm)	12.37	4.16
Seedling length (cm)	12.54	5.90
Seedling dry weight (g)	9.10	1.51
Salinity tolerance index (STI)	7.20	0.04
Seedling vigor index (SVI)	13.96	3.29

**Figure 3:**
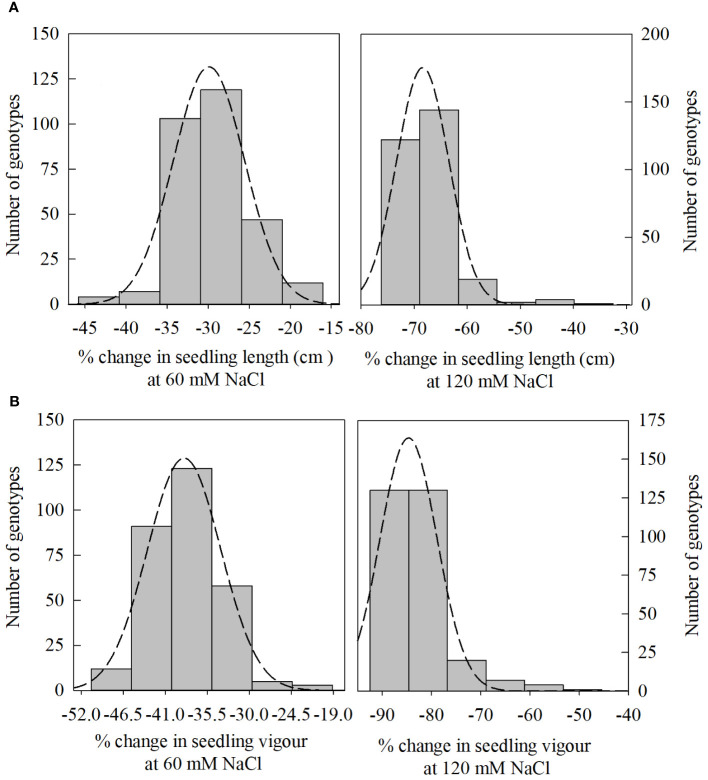
Distribution of change (%) in seedling length [**(A)**; 60, and 120 mM NaCl] and seedling vigor index (SVI) [**(B)**; 60, and 120 mM NaCl] from control treatment (0 NaCl) to different salinity levels (60, and 120 mM NaCl) across different winter wheat genotypes (n*=*292) in experiment 1 at germination (Feekes 0.9) and early seedling (Feekes 1.0) stages.

**Figure 4:**
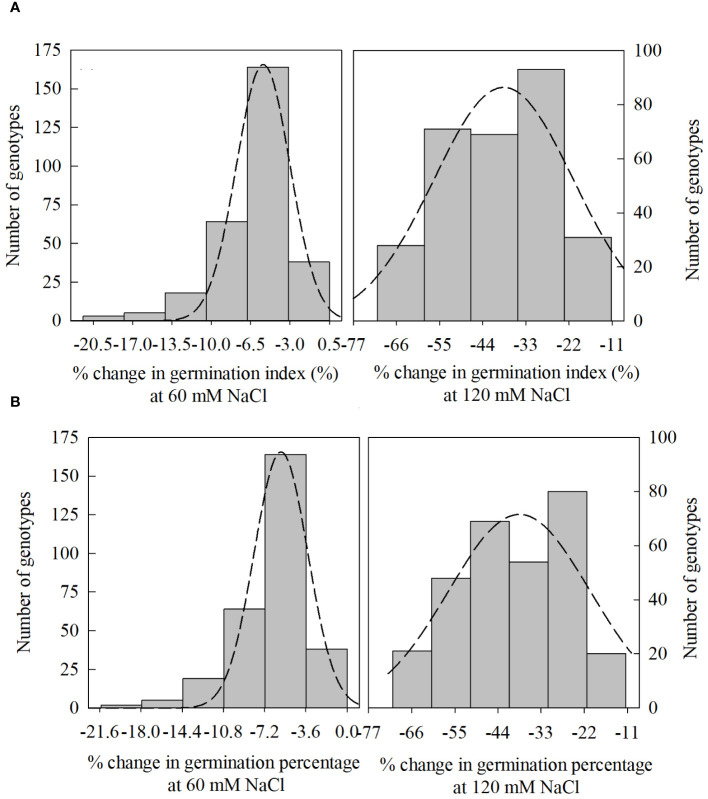
Distribution of change (%) in germination index [**(A)**; 60, and 120 mM NaCl] and germination percentage [**(B)**; 60, and 120 mM NaCl] from control treatment (0 NaCl) to different salinity levels (60, and 120 mM NaCl) across different winter wheat genotypes (n*=*292) in experiment 1 at germination (Feekes 0.9) and early seedling (Feekes 1.0) stages.

In PC1, seedling vigor index (SVI; 14.0%) and seedling length (12.5%) were the traits that contributed more to the differential response of genotypes as compared with other traits. Simultaneously, germination index (GI; 31.9%) and germination percent (G%; 31.7%) were the traits that showed more contribution in PC2 (**Table 3**). The magnitude of changes in the above four traits of 292 genotypes was randomly distributed in different ranges (**Figures 3**, **4**). Among these four traits, the SVI, which showed a high contribution in principal component (PC1) with a high variance of the total variance was selected to identify the extremely contrasting genotypes in response to salinity (**Supplementary Table 3**). Hence, genotypes that showed the highest and lowest percentage of reduction in SVI under the highest-level salinity (120 mM NaCl) were selected and classified, which were (1) tolerant (Gage, Guymon, MTS0531 and Tascosa [lowest reduction in SVI]); and (2) susceptible (CO04W320, Carson, TX04M410211 and 2174–05 [highest reduction in SVI]). These two categories of genotypes were used for further evaluation to understand the response to salinity stress at the booting stage (experiment 2).

The changes in germination and early seedling traits of selected genotypes of the tolerant and susceptible categories from experiment 1 are discussed below. The results are discussed by comparing the plants grown in the control treatment with those grown at 120 mM NaCl level due to the highly significant changes as compared to the 60 mM NaCl level. There were distinguishable changes observed in studied germination and early seedling traits between the two different categories of genotypes at 120 mM NaCl salinity level (**Table 4**).

Table 4Variation in germination and early seedling traits of selected salinity tolerant (n=4) and suspectable (n=4) winter wheat genotypes exposed to control treatment (0 mM NaCl) and different levels of salinity (60, and 120 mM NaCl) at germination (Feekes 0.9) and early seedling (Feekes 1.0) stages (experiment 1).

**Table:** 

Traits	Salinity tolerant genotypes											
	Gage			Guymon			MTS0531			Tascosa		
	Salinity levels (mM NaCl)											
	0	60	120	0	60	120	0	60	120	0	60	120
Germination traits												
Germination Percentage (G; %)	99^a^	96^b^	85(-14%)^a^	99^a^	96^b^	86(-13%)^a^	99^a^	99^a^	88(-11%)^a^	98^a^	95^b^	86(-12%)^a^
Germination Index (GI; %)	100^a^	98^a^	86(-14%)^a^	100^a^	98^a^	87(-13%)^a^	100^a^	100^a^	89(-11%)^a^	100^a^	98^a^	89(-11%)^a^
Mean daily germination (MDG)	8.21^a^	4.81^ab^	4.39(-47%)^a^	8.21^a^	5.63^a^	4.31(-48%)^a^	8.21^a^	4.85^ab^	4.77(-42%)^a^	8.08^a^	5.13^a^	4.09(-49%)^a^
Germination rate (GR; d^-1^)	2.02^aa^	2.59^b^	2.62(+30%)^a^	2.02^a^	2.51^b^	2.49(+23%)^a^	2.06^a^	2.67^b^	2.53(+23%)^a^	2.02^a^	2.47^b^	2.65(+31%)^a^
Early seedling traits												
Shoot length (cm)	8.05^a^	6.53^a^	4.45(-45%)^a^	8.30^a^	6.98^a^	5.60(-33%)^a^	8.25^a^	6.35a	4.43(-46%)^a^	8.20^a^	6.63^a^	4.38(-47%)^a^
Root length (cm)	7.20^a^	5.40^a^	3.43(-52%)^a^	7.27^a^	5.73^a^	4.13(-43%)^a^	7.22^a^	5.20a	3.63(-50%)^a^	7.20^a^	5.33^a^	3.48(-52%)^a^
Seedling length (cm)	15.25^a^	11.93^a^	7.88(-48%)^b^	15.57^a^	12.71^a^	9.73(-38%)^a^	15.47^a^	11.55a	8.06(-48%)^a^	15.40^a^	11.95^a^	7.86(-49%)^b^
Seedling dry weight (g)	0.052^b^	0.04^b^	0.03(-40%)^a^	0.061^a^	0.052^a^	0.031(-50%)^a^	0.053^b^	0.042b	0.031(-40%)^a^	0.062^a^	0.051^a^	0.032(-50%)^a^
Seedling vigor index (SVI)	15.06^a^	11.48^a^	6.69(-56%)^a^	15.37^a^	12.22^a^	8.39(-45%)^a^	15.31^a^	11.41a	7.04(-54%)^a^	15.02^a^	11.35^a^	6.77(-55%)^a^
Salt tolerance index (STI)	100^a^	81.15^a^	52.25(-48%)^a^	100^a^	80.10^a^	52.85(-47%)^a^	100^a^	79.65a	50.45(-50%)^a^	100^a^	79.25^a^	51.00(-49%)^a^

**Table:** 

Traits	Salinity suspectable genotypes											
	CO04W320			Carson			TX04M410211			2174–05		
	Salinity levels (mM NaCl)											
	0	60	120	0	60	120	0	60	120	0	60	120
Germination traits												
Germination Percentage (G; %)	99^a^	78^d^	36(-64%)^b^	99^a^	86^c^	36(-64%)^b^	99^a^	95^b^	39(-61%)^b^	99^a^	88^c^	29(-71%)^b^
Germination Index (GI; %)	100^a^	79^b^	31(-69%)^b^	100^a^	87^b^	30(-70%)^b^	100^a^	96^a^	39(-61%)^b^	100^a^	89^ab^	29(-71%)^b^
Mean daily germination (MDG)	7.38^ab^	4.50^ab^	1.41(-81%)^b^	7.38^ab^	3.88^b^	1.47(-80%)^b^	7.38^ab^	4.04^b^	1.42–81%)^b^	7.38^ab^	3.93^b^	1.11(-85%)^b^
Germination rate (GR; d^-1^)	2.05^a^	2.63^b^	3.37(+64%)^b^	2.08^a^	3.02^a^	3.26(+57%)^b^	2.06^a^	2.79^b^	3.35(+63%)^b^	2.08^a^	2.74^b^	3.67(+76%)^b^
Early seedling traits												
Shoot length (cm)	8.58^a^	5.95^b^	3.87(-55%)^b^	8.60^a^	6.25^a^	4.40(-49%)^a^	8.60^a^	6.45^a^	3.03(-65%)^b^	8.20^a^	5.45^b^	2.70(-67%)^c^
Root length (cm)	7.30^a^	5.08^a^	2.35(-68%)^b^	7.53^a^	4.98^ab^	2.41(-68%)^b^	7.50^a^	4.90^b^	1.93(-74%)^b^	7.10^a^	4.38^b^	1.60(-77%)^b^
Seedling length (cm)	15.88^a^	11.03^a^	6.22(-61%)^c^	16.13^a^	11.23^a^	6.81(-58%)^c^	16.10^a^	11.35^a^	4.96(-69%)^d^	15.30^a^	9.83^b^	4.30(-72%)^d^
Seedling dry weight (g)	0.068^a^	0.043^b^	0.018(-73%)^b^	0.069^a^	0.041^b^	0.023(-67%)^b^	0.065^a^	0.038^b^	0.019(-72%)^b^	0.072^a^	0.042^b^	0.016(-78%)^b^
Seedling vigor index (SVI)	15.68^a^	8.54^b^	2.3(-86%)^b^	15.92^a^	9.68^ab^	2.47(-84%)^b^	15.99^a^	10.78^a^	1.92(-88%)^b^	15.11^a^	8.59^b^	1.24(-92%)^b^
Salt tolerance index (STI)	100^a^	55.5^b^	27.6(-72%)^b^	100^a^	58.70^b^	32.7(-67%)^b^	100^a^	59.05^b^	28.9(-71%)^b^	100^a^	58.05^b^	22.5(-77%)^b^

Values in parenthesis indicate the percent differences from the control (0 mol/L NaCl) to the highest level of salinity (120 mM NaCl). (-) indicate percentage reduction and (+) indicates percentage increase. Values followed by different letters are significantly different according to Duncan’s multiple range test (DMRT; P < 0.05).

#### 3.1.2 Variation in germination and early seedling traits

Among the selected eight genotypes, genotype 2174–05 from the susceptible category showed the highest percentage decrease in all germination traits, except GR, under the highest salinity level of 120 mM NaCl as compared to the control treatment (0 mM NaCl), while it showed highest percentage increase in GR. On the other hand, the least percentage reduction in G% and mean daily germination (MDG) was observed in MTS0531 from the tolerant category. At the same time, Guymon and MTS0531 showed the least increase in GR, and Tascosa and MTS0531 highest increase in GI as compared to the other genotypes (**Table 4**).

All the early seedling traits were reduced under higher salinity treatment. The least percentage reduction in shoot, root, and seedling length, SVI, dry weight, and STI was observed in Guymon. At the same time, a higher reduction in the above traits was found in 2174–05 (**Table 4**).

### 3.2 Experiment 2

Highly significant changes (*P*<0.01) were observed in most of the studied physiological and biochemical traits of selected eight genotypes treated with higher salinity level (120 mM NaCl) at the booting stage. However, the effect of diverse genotypes on individual grain weight and harvest index (HI) was non-significant, while other agronomic (*P*<0.05), physiological (*P*<0.01), and biochemical (*P*<0.001) traits were significant (**Table 5**). The interaction effect of salinity and genotype were significant (*P*<0.05) in different traits such as photosynthetic rate (*P*_N_), thylakoid membrane damage (F0/Fm), chlorophyll index, total soluble sugars, reducing sugars, proline, total protein, and lipid peroxidation, while all the agronomic traits were nonsignificant (**Table 4**). The studied traits showed different magnitudes of response across genetically diverse genotypes when exposed to higher levels of salinity (**Tables 6**, **7**).

**Table 5:** Probability values of effects of salinity (S), genotype (G), and interaction (S x G) on physiological, biochemical, and agronomic traits of selected salinity tolerant (n=4) and suspectable (n=4) winter wheat genotypes at the booting (Feekes 10.0) stage (experiment 2).

	Variables		
Traits	Salinity (S)	Genotype (G)	S x G
Physiological traits			
Maximum quantum yields of PS II (Fv/Fm)	<0.001	<0.001	0.304
Thylakoid membrane damage (F0/Fm)	<0.001	<0.001	<0.001
Photosynthetic rate (P_N_; µmol m^-2^ s^-1^)	<0.001	<0.001	<0.001
Stomatal Conductance (*g*_s_; mmol m² s¹)	<0.001	<0.001	0.0424
Intrinsic water use efficiency (iWUE)	<0.001	<0.01	<0.01
Chlorophyll index (SPAD)	<0.001	<0.001	<0.001
Biochemical traits			
Total soluble sugars (g kg^-1^)	<0.001	<0.001	<0.001
Reducing sugars (g kg^-1^)	<0.001	<0.001	<0.001
Non-reducing sugars (g kg^-1^)	<0.001	<0.001	0.187
Starch (g kg^-1^)	<0.001	<0.001	0.723
Proline (µmol g^-1^)	<0.001	<0.001	<0.05
Total protein (g kg^-1^)	<0.001	<0.001	<0.001
MDA (µmo/g^-1^)	<0.001	<0.001	<0.001
Agronomic traits			
Spikelet number (spike ^-1^)	<0.001	<0.001	0.845
Total dry weight (g plant^-1^)	<0.001	<0.05	0.997
Grain number (plant^-1^)	<0.001	<0.001	0.997
Individual grain weight (mg)	<0.001	0.970	0.650
Total grain yield (g plant^-1^)	<0.001	<0.001	0.991
Harvest index (HI; %)	<0.001	0.190	0.989

Table 6Variation in physiological and biochemical traits of selected salinity tolerant (n=4) and suspectable (n=4) winter wheat genotypes exposed to control treatment (0 mM NaCl) and a higher level of salinity (120 mM NaCl) at the booting stage (Feekes 10.0) (experiment 2).

**Table:** 

Traits	Salinity tolerant genotypes							
	Gage		Guymon		MTS0531		Tascosa	
	Salinity levels (mM NaCl)							
	0	120	0	120	0	120	0	120
Physiological traits								
Maximum quantum yields of PS II (Fv/Fm)	0.764^b^	0.707 (-7%)^b^	0.789^a^	0.739 (-6%)^a^	0.766^b^	0.704 (-8%)^b^	0.787^a^	0.736 (-6%)^a^
Thylakoid membrane damage (F0/Fm)	0.183^a^	0.208 (+14%)^a^	0.178^a^	0.192 (+8%)^b^	0.184^a^	0.205 (+11%)^a^	0.184^a^	0.181 (+2%)^b^
Photosynthetic rate (*P*_N_; µmol m^-2^ s^-1^)	18.39^a^	15.29 (-17%)^b^	19.89^a^	16.99 (-15%)^a^	18.33^a^	15.66 (-15%)^b^	19.25^a^	17.04 (-11%)^a^
Stomatal Conductance (*g*_s_; mmol m² s¹)	0.79^b^	0.56 (-29%)^b^	0.78^b^	0.63 (-20%)^a^	0.82^a^	0.56 (-32%)^b^	0.811a	0.63 (-23%)^a^
Intrinsic water use efficiency (*P*_N_/*g*_s_; iWUE)	23.3^b^	27.3 (+17.3)^a^	25.5^a^	26.97 (+5.8)^a^	22.3^b^	28.0 (+25.3)^a^	23.7^ab^	27.0 (+14.0)^a^
Chlorophyll index (SPAD)	54.75^a^	48.72 (-11%)^a^	55.83^a^	50.9 (-9%)^a^	53.87^a^	48.60 (-10%)^a^	54.23^a^	51.01 (-6%)^a^
Biochemical traits								
Total soluble sugars (g kg^-1^)	66.5^ab^	80.7 (+21%)^b^	65.9^ab^	82.8 (+26%)^b^	64.2^ab^	83.1 (+29%)^a^	65.7^ab^	84.6 (+29%)^a^
Reducing sugars (g kg^-1^)	48.3^a^	58.1 (+20%)^b^	47.4^a^	58.9 (+24%)^b^	45.9^b^	59.8 (+30%)^ab^	49.4^a^	61.3 (+24%)^a^
Non-reducing sugars (g kg^-1^)	18.2^a^	22.6 (+24%)^ab^	18.8^a^	23.8 (+27%)^a^	18.3^a^	23.4 (+28%)^a^	16.3^b^	23.3 (+43%)^a^
Starch (g kg^-1^)	82.9^ab^	75.0 (-10%)^b^	82.9^ab^	74.2 (-10%)^b^	79.4^b^	69.5 (-12%)^c^	84.4^a^	76.1 (-10%)^b^
Proline (µmol g^-1^)	2.92^a^	4.77 (+63%)^b^	3.31^a^	5.50 (+66%)^a^	2.69^b^	4.54 (+69%)^b^	3.19^a^	5.77 (+81%)^a^
Total soluble protein (g kg^-1^)	12.9^a^	15.7 (+22%)^ab^	13.1^a^	18.0 (+37%)^a^	12.9^a^	15.3 (+19%)^ab^	13.4^a^	18.2 (+36%)^a^
Lipid peroxidation (MDA; µmol g^-1^)	2.42^a^	4.03 (+67%)^a^	2.00^a^	3.38 (+69%)^ab^	2.54^a^	4.10 (+61%)^a^	2.07^a^	3.38 (+63%)^ab^

**Table:** 

Traits	Salinity suspectable genotypes							
	CO04W320		Carson		TX04M410211		2174–05	
	Salinity levels (mM NaCl)							
	0	120	0	120	0	120	0	120
Physiological traits								
Maximum quantum yields of PS II (Fv/Fm)	0.769^b^	0.701 (-9%)^b^	0.771^ab^	0.704 (-9%)^b^	0.788^a^	0.742 (-6%)^a^	0. 779^ab^	0.712 (-9%)^b^
Thylakoid membrane damage (F0/Fm)	0.182^a^	0.203 (+12%)^a^	0.184^a^	0.207 (+13%)^a^	0.175^b^	0.189 (+8%)^ab^	0.185^a^	0.204 (+10%)^a^
Photosynthetic rate (*P*_N_; µmol m^-2^ s^-1^)	19.14^a^	15.13 (-21%)^b^	18.79^a^	15.13 (-19%)^b^	19.36^a^	16.84 (-13%)^ab^	19.11^a^	15.07 (-21%)^b^
Stomatal Conductance (*g*_s_; mmol m² s¹)	0.78^b^	0.55 (-29%)^b^	0.83^a^	0.55 (-33%^)b^	0.81^a^	0.65 (-20%)^a^	0.80^a^	0.53 (-34%)^b^
Intrinsic water use efficiency (*P*_N_/*g*_s_; iWUE)	24.5^a^	27.5 (+12.1)^a^	22.6^b^	27.5 (+21.5)^a^	23.9^ab^	25.9 (+8.4)^b^	23.9^ab^	28.4 (+19.0)^a^
Chlorophyll index (SPAD)	54.13^a^	49.39 (-9%)^a^	54.69^a^	49.49 (-10%)^a^	55.02^a^	50.5 (-8%)^a^	55.24^a^	48.73 (-12%)^a^
Biochemical traits								
Total soluble sugars (g kg^-1^)	62.6^b^	80.6 (+29%)^b^	67.2^a^	82.9 (+23%)^b^	67.7^a^	85.4 (+26%)^a^	69.3^a^	83.5 (+20%)^a^
Reducing sugars (g kg^-1^)	45.3^b^	56.0 (+24%)^b^	52.0^a^	60.5 (+16%)^a^	51.1^a^	62.2 (+22%)^a^	49.7^a^	59.4 (+20%)^ab^
Non-reducing sugars (g kg^-1^)	17.3^a^	24.6 (+42%)^a^	15.2^b^	22.4 (+47%)^ab^	16.6^b^	23.2 (+40%)^a^	19.6^a^	24.2 (+23%)^a^
Starch (g kg^-1^)	81.0^ab^	74.9 (-8%)^b^	86.5^a^	75.2 (-13%)^b^	84.5^b^	75.4 (-11%)^b^	83.4^ab^	80.9 (-3%)^a^
Proline (µmol/g^-1^	2.73^b^	5.11 (+87%)^a^	3.15^a^	4.81 (+53%)^b^	3.27^a^	5.85 (+79%)^a^	3.11^a^	4.81 (+55%)^b^
Total soluble protein (g kg^-1^)	12.9^a^	15.4 (+19%)^ab^	13.6^a^	15.9 (+17%)^ab^	13.3^a^	17.7 (+33%)^a^	13.3^a^	16.5 (+24%)^ab^
Lipid peroxidation (MDA; µmol g^-1^)	2.39^a^	3.99 (+67%)^a^	2.37^a^	4.02 (+70%)^a^	2.01^a^	3.44 (+71%)^ab^	2.45^a^	3.96 (+62%)^a^

Values in parenthesis indicate the percent differences from the control treatment (0 mol/L NaCl) to the highest level of salinity (120 mM NaCl). (-) indicate percentage reduction and (+) indicates percentage increase. Values followed by different letters are significantly different according to Duncan’s multiple range test (DMRT; P < 0.05).

Table 7Variation in agronomic traits of selected salinity tolerant (n=4) and suspectable (n=4) winter wheat genotypes after exposed control treatment (0 mM NaCl) and different levels of salinity (60, and 120 mM NaCl) at the booting (Feekes 10.0 stage (experiment 2).

**Table:** 

Traits	Salinity tolerant genotypes							
	Gage		Guymon		MTS0531		Tascosa	
	Salinity levels (mM NaCl)							
	0	120	0	120	0	120	0	120
Agronomic traits								
Spikelet number (spike ^-1^)	19.2^a^	15.7 (-18%)^a^	19.2^a^	16.2. (-16%)^a^	18.8^a^	15.6 (-17%)^a^	19.2a	16.2 (-16%)^a^
Grain number (plant^-1^)	271^a^	207 (-24%)^b^	252^a^	215 (-15%)^b^	265^a^	211 (-20%)^b^	249a	229 (-8%)^a^
Individual grain weight (mg)	40.0^a^	33.1 (-17%)^a^	40.3^a^	33.0 (-18%)^a^	39.9^a^	32.9 (-18%)^a^	41.1a	32.9 (-20%)^a^
Grain yield (g plant^-1^)	11.2^a^	6.9 (-38%)^a^	11.1^a^	7.6 (-32%)^a^	10.9^a^	7.0 (-36%)^a^	11.4a	7.6 (-33%)^a^
Total dry weight (g plant^-1^)	21.1^a^	19.7 (-7%)^a^	20.7^a^	18.6 (-10%)^a^	20.7^a^	18.5 (-11%)^a^	20.9a	19.6 (-6%)^a^
Harvest index (HI; %)	53.1^a^	35.0 (-34.0)^c^	53.6a	40.9 (-23.8)^a^	52.7a	37.8 (-28.1)^b^	54.5a	38.8 (-28.9)^b^

**Table:** 

Traits	Salinity suspectable genotypes							
	CO04W320		Carson		TX04M410211		2174–05	
	Salinity levels (mM NaCl)							
	0	120	0	120	0	120	0	120
Agronomic traits								
Spikelet number (spike ^-1^)	19.3^a^	15.7 (-19%)^a^	19.2^a^	15.6 (-19%)^a^	19.3^a^	16.3 (-17%)^a^	18.8^a^	15.5 (-18%)^a^
Grain number (plant^-1^)	262^a^	204 (-22%)^b^	267^a^	204 (-24%)^b^	254^a^	225 (-11%)^a^	252^a^	201 (-20%)^b^
Individual grain weight (mg)	41.2^a^	32.5 (-21%)^a^	39.2^a^	33.7 (-14%)^a^	40.9^a^	32.3 (-21%)^a^	41.2^a^	33.6 (-18%)^a^
Grain yield (g plant^-1^)	11.0^a^	6.8 (-38%)^a^	10.8^a^	7.1 (-34%)^a^	11.1^a^	7.5 (-32%)^a^	10.7^a^	6.9 (-36%)^a^
Total dry weight (g plant^-1^)	20.7^a^	18.7 (-10%)^a^	20.4^a^	18.3 (-10%)^a^	21.5^a^	18.7 (-13%)^a^	20.9^a^	19.4 (-7%)^a^
Harvest index (HI; %)	53.1^a^	36.4 (-31.6)^c^	52.9^a^	38.8 (-26.7)^b^	51.6^b^	40.1 (-22.3)^a^	51.2	35.6 (-30.5)^c^

Values in parenthesis indicate the percent differences from the control treatment (0 mol/L NaCl) to the highest level of salinity (120 mM NaCl). (-) indicate percentage reduction and (+) indicates percentage increase. Values followed by different letters are significantly different according to Duncan’s multiple range test (DMRT; P< 0.05).

#### 3.2.1 Response of physiological traits

##### 3.2.1.1 Leaf photosynthetic rate, chlorophyll fluorescence, and chlorophyll index

The *P*_N_, maximum quantum yield of PS II (Fv/Fm), and chlorophyll index reduced at higher level of salinity, while F0/Fm and intrinsic water use efficiency (iWUE) were increased significantly across genotypes (**Table 6**). Among the genotypes, Tascosa showed the least reduction in *P*_N_ and chlorophyll index; and a non-significant least increase in F0/Fm. The increase in iWUE was highest in MTS0531 and Carson with the highest reduction in stomatal conductance (*g_s_*). At the same time, CO04W320, Carson, and 2174–05 showed the highest reduction in Fv/Fm, while the least was in Guymon and Tascosa. On the other hand, the highest increase in F0/Fm was observed in Gage and the highest reduction in chlorophyll index was 2174–05 (**Table 6**).

#### 3.2.2 Response of biochemical traits

##### 3.2.2.1 Sugars and starch

The total soluble sugars, reducing sugars, and non-reducing sugars were increased across genotypes at the higher level of salinity, while starch concentration was reduced (**Table 6**). The genotypes MTS0531, Tascosa, and CO04W320 showed the highest percentage increase in total soluble sugars. At the same time, Carson showed the highest increase in non-reducing sugars and the highest reduction in starch. The least increase in non-reducing sugars and reduction in starch was observed in 2174–05 (**Table 6**).

##### 3.2.2.2 Total protein, proline, and Lipid peroxidation

The total protein and proline, and lipid peroxidation (malondialdehyde; MDA) were increased across all the genotypes at the higher level of salinity (**Table 6**). Among the genotypes, the highest increase in total protein was observed in Guymon followed by Tascosa, while it was the least in Carson and MTS0531. Simultaneously, proline was high in CO04W320 and followed by Tascosa Tascosa, while the least increase was observed in Carson. Genotype TX04M410211 showed the highest increase in MDA followed by Carson. At the same time, the least reduction in MDA was found in MTS0531 (**Table 6**).

#### 3.2.3 Variation in agronomic traits

All the agronomic traits recorded were reduced across genotypes after exposure to higher level of salinity at booting stage (**Table 7**). Tascosa showed the least reduction in total dry weight and grain number, while the least reduction in spikelet number was in Guymon and Tascosa. Simultaneously, the least reduction in individual grain weight was observed in Carson and grain yield in Guymon and Tascosa. At the same time, the highest reduction in grain yield was in Gage and CO04W320. The HI was reduced more in Gage and least in TX04M410211 (**Table 7**).

## 4 Discussion

Even though the wheat is moderately tolerant to salinity (Dubcovsky and Dvora, 2007), our study demonstrated the diverse response across 292 winter wheat genotypes at germination to early seedling (Feeks 0.9 to 1.0) stage exposed to different salinity levels (60 and 120 mM NaCl). The results specify the importance of systematic screening of germplasm collections to understand the diverse salinity responses to identify the candidate genotypes for breeding, crop improvement, and salinity management programs. There were inconsistencies in the magnitude of the final grain yield of selected eight genotypes, which were assumed to be high salinity tolerant (Gage, Guymon, MTS0531 and Tascosa [lowest reduction in SVI) and suspectable (CO04W320, Carson, TX04M410211 and 2174–05 [highest reduction in SVI]) genotypes based on SVI (experiment 1; **Table 4**), treated with higher level of salinity (120 mM NaCl) at booting (Feeks 10.0) stage. A mixed response in grain yield was observed among the eight selected genotypes in experiment 2 (**Table 7**). Based on the actual grain yield and its percentage reduction as compared to the control treatment, Guymon and TX04M410211 were the tolerant genotypes, and Gage and CO04W320 were the susceptible genotypes under higher levels of salinity at the booting stage. Such contrasting responses of genotypes imply that the sensitivity and adaptability of genotypes to salinity can vary with different sensitive growth stages. At the same time, several other factors such as duration of exposure and intensity of salinity that are determined by mode occurrence of salinity can play a major role in the fluctuating salinity response of genotypes as well.

In other words, since the mode of occurrences of salinity in a particular agroclimatic region is natural and/or anthropogenic, the time (growth stages) and duration of exposure and intensity of salinity can vary which can influence the multi-traits responses of genotypes with diverse genetic backgrounds result in changes in the magnitude of final grain yield. The regions in or near saline areas (salt lakes, salt pans, salt marshes, and salt flats) with considerably high soil salinity levels are counted as natural modes of occurrences of salinity and anthropogenic mode is the exposure of salinity to soil through poor quality irrigation water due to the intrusion of seawater and contamination from effluents from industrial wastewater (Zaeri et al., 2023). As the salient findings of the current study are not in alignment with the proposed hypothesis leads to the recommendation of deciphering the genotypic responses more precisely and specifically at each sensitive growth stage with an emphasis on the duration of exposure and intensity of salinity. First and foremost, the salinity screening for tolerant genotypes for a specific agroclimatic region should consider the mode of occurrence of salinity which is unavoidable to reach beyond-doubt conclusions. However, the results of the current study were discussed without any pre-conceptualization about the unique response of genotypes.

In this study, the diverse response of genotypes starts from the seed germination traits and is accompanied by early seedling traits (**Table 2**). Similar results were reported from earlier studies by Rahman et al. (2008); Khayatnezhad and Gholamin (2010), and Kumar et al. (2012). Salinity affects seed germination in two ways (1) a high concentration of salt in the growth medium reduces the osmotic potential to a level that prevents water uptake and reduces utilization of nutrients that are essential for germination, and (2) the toxic effect of Na^+^ and Cl^-^ ions on embryo (Kayani et al., 1990; Munns et al., 2006). Besides, the magnitude of germination traits of genotypes varied under different salinity levels (0 [control], 60, and 120 mM NaCl; **Table 2**, **4**) with clear evidence of linearity in adverse change which is reported to be unfavorable for tolerance response to salinity. It appears that, even at a higher salinity level of 120 mM NaCl, the water potential of the seeds was still sufficiently low to bring an adequate amount of water for the several metabolic processes that lead to germination, but inadequate for further growth and development (Muhammad and Hussain, 2012). And the results of this study are analogous to the findings described by other researchers (Kazemi and Eskandari, 2011; Muhammad and Hussain, 2012).

The salinity has a negative impact on different physiological and biochemical processes which can significantly reduce cell division and expansion, and such changes in the cellular level reduce early seedling traits such as shoot, and root length and seedling dry weight (**Table 4**) and biomass accumulation is relatively dependent on shoot and root lengths and branches. The results obtained in this study were consistent with previous findings that have indicated significant differences in the salinity tolerance of wheat genotypes and their differential responses to increased salinity levels (Rahman et al., 2008; Adjel et al., 2013).

The PCA is a multivariate statistical data analysis technique that revealed the SVI and seedling length (shoot and root length) are important traits monitored in experiment 1 and were assumed to be indicators and responsible for salinity tolerance followed by GI and G% (**Table 2**). These traits contributed more toward the diverse response of 292 genotypes to salinity among the studied germination and early seedling traits. However, the genotypes with higher G% also showed higher root length, shoot length, and dry matter production. It is estimated that in addition to higher dry weight, longer shoot and root development are more important for selection for high salt tolerance. Yet, shoot and root length and dry weight can be considered as selection criteria only when there is a high germination percentage. For these reasons, the SVI, which shows a function of both G% and seedling length, was determined to be a more reliable trait for the selection criterion at germination to the early seedling stage and was used for classifying genotypes into contrasting categories (salinity tolerant and susceptible).

However, the selected eight genotypes from experiment 1 showed significant variation among the studied physiological and biochemical traits in experiment 2 after being treated with a higher salinity level (120 mM NaCl) at the booting stage (**Tables 5**, **6**). But the response did not follow the expected magnitude of trend in salinity responses (tolerance and susceptibility based on SVI) and that was reflected in the final yield (**Table 7**). Response of physiological traits in current study indicates that there was a significant reduction in interrelated traits such as *P*_N_, *g*_s_, iWUE, chlorophyll index, Fv/Fm, and an increase in F0/Fm exposed to high salinity level, which can be attributed to the negative effect of salinity on chlorophyll synthesis, photosynthetic enzymes, thylakoid membrane, photosystems and stomatal regulation and chlorophyll degradation (Djanaguiraman and Prasad, 2013; Sabbagh et al., 2014; Hannachi et al., 2022). At the same time, maintenance and/or least reduction of *P*_N_ and *g*_s_ resulted in high iWUE under salinity can be related to the capacity of tolerant genotypes acquired by successful osmotic regulation, compartmentalization of the salts in the vacuole and/or excretion of salt ions from the cytosol (Munns and Tester, 2008; Sabbagh et al., 2014; Hannachi et al., 2022).

Further, under diverse stress conditions, plants are induced to create changes in several metabolites such as sugars (reducing [glucose and fructose] and non-reducing sugars [sucrose]), starch, protein, and proline (Laxman et al., 2013; Sunoj et al., 2016; Ozturk et al., 2021). Among these metabolites, sugars, protein, and proline are known as osmolytes or osmoprotectants take part in osmotic regulation (Laxman et al., 2013; Ozturk et al., 2021; Biradar et al., 2022). Osmotic tolerance is vital for the plant to survive and maintain its growth rate by preserving optimum photosynthesis at a high salinity level. It can be achieved by maintaining cell volume and turgor, importantly in the guard cells of stomata, during exposure to sanity by osmotic adjustments or regulation (Munns and Tester, 2008). In fact, the accumulation of soluble inorganic and organic compounds in plants has been widely reported as a response to salinity (Hamada and Khulaef, 1995; Yang et al., 2009; Radi et al., 2013; Sabbagh et al., 2014; Dadkhah and Rassam, 2016). Some plants withstand salinity by reducing the cellular osmotic potential because of a net increase in inorganic and organic solute accumulation (Yang et al., 2009; Sabbagh et al., 2014).

In the current study, higher salinity level induced an accumulation of total soluble sugars, protein, and proline across genotypes (**Table 6**). It is well known that soluble sugars play an important role in plant metabolism such as products of hydrolytic processes, substrates in biosynthetic processes, and energy production, and act as cellular osmotic regulators (Sunoj et al., 2020; Biradar et al., 2022). Numerous studies have tried to link the increase of soluble sugars to salinity tolerance and the present result agrees with results from wheat genotypes treated with higher salinity level resulted in a significant increase in sugars and proline in some genotypes (Dadkhah and Rassam, 2016; Akbar et al., 2021, 2022). Simultaneously, proline is also a critical component for osmoprotection in many plants, and one of the most common responses of many plants subjected to abiotic stresses is its accumulation (Goharrizi et al., 2020; Liu et al., 2020; Masarmi et al., 2023). It has been reported that proline plays a protective role in plants exposed to diverse stresses and is thought to be acting as a cellular osmotic regulator. And along with other enzymatic and non-enzymatic ROS quenching mechanisms proline regulates increased ROS production due to the reduced light-dependent and CO_2_ assimilation reactions of photosynthesis (Munns and Tester, 2008; Aycan et al., 2022; Singh et al., 2022; Sunoj et al., 2022). A study reported the genotypic differences in proline accumulation under salinity were seen in wheat genotypes (Tavakoli et al., 2016). On the other hand, an increase of specific proteins such as osmotin, germin, and total soluble protein was reported in different crops, especially in the genotypes, which are tolerant of high level of salinity (Athar et al., 2022). Studies reported that in wheat genotypes, different proteins play important roles under salinity such as in osmotic regulation and storage pool on nitrogen, which can be utilized in growth and development (Radi et al., 2013; Zhu et al., 2021; Masarmi et al., 2023).

Starch is the most abundant storage carbohydrate produced in plants that high related to sugar metabolism and photosynthesis and is reduced across all the genotypes in the current study because of higher salinity level (**Table 7**). Our result herein agreed with studies on the effect of salinity on wheat (Hadif and Ibrahim, 2021; Sezer et al., 2021). The reduced starch under salinity can be due to lower photosynthesis and changes in starch-sugar interconversion-related enzymes involved in starch synthesis and degradation and it can also be related to the increased sugars in wheat genotypes (Dubey and Singh, 1999; Yin et al., 2010). A higher percentage reduction in non-reducing sugars in wheat genotypes as compared to the reducing sugars can be an indication of higher inhibition of starch biosynthesis enzymes. Another explanation of the high sugar content in wheat leaves at the same time reduction in starch content can be due to the inhibition of the distribution of these sugars to storage tissues. Furthermore, feedback inhibition from sink to source due to the accumulation of sugars can also result in a reduction in photosynthesis and starch. However, there are studies that reported contrasting results as well which stated that the starch in wheat leaves increased with salinity (Dadkhah and Rassam, 2016; Sadak, 2019).

The MDA is a product of oxidative stress by ROS that peroxidizes the polyunsaturated fatty acids (PUFA) in lipids of cell membranes. Quantification of MDA is frequently used as an indicator of cell membrane stability and permeability, electrolyte leakage, and imbalance in osmotic regulation under stress conditions (Lu et al., 2017; Biradar et al., 2022). Likewise, MDA has been considered an indicator of salt-induced oxidation in cell membranes and a tool for determining salt tolerance in plants (Ghafiyehsanj et al., 2013; Radi et al., 2013; Zeeshan et al., 2020). In this study, MDA was significantly increased under salinity stress. However, the accumulation of MDA content was increased to a greater degree in some genotypes than in other genotypes, this suggests that within the genotypes tested, there were some susceptible genotypes that accumulated more MDA. Many studies agree that increasing MDA is linked with increasing the degree of stress in wheat (Mansoor and Naqvi, 2013; Shamloo-Dashtpagerdi et al., 2022; Singh et al., 2022) and that genotypes that accumulate less MDA are more tolerant.

In wheat, yield is determined by the number of spikes per plant and yield components such as spikelet number, grain number, and grain weight (Mizuno et al., 2021; Matkovic Stojsin et al., 2022; Roychowdhury et al., 2023). The result from this study showed that spikelet number per spike had a positive and highly significant relationship with grain yield under salinity stress. This result agrees with other studies under salinity conditions (Sairam et al., 2002; Ashik et al., 2021). In this study, the individual grain weight and number of grains were least sensitive to salinity. This is because grain weight is determined between flowering and maturity, which was after the exposure of salinity in this study and reported earlier (Matkovic Stojsin et al., 2022). A reduction of spikelet number per spike was observed after salinity exposure, which can be due to the fact that spikelet number initiation occurs at the vegetative stage, and salinity may have resulted in shortening the vegetative stage, in turn causing a reduction in a number of spikelets per spike. This agreed with another study which reported a positive correlation between the length of the vegetative phase and the number of spikelet number per spike (Rahman et al., 2008; Fatima et al., 2020; El Sabagh et al., 2021). The reduction in the above yield-related traits resulted in the reduction of grain yield and HI, which is positively related to hostile environmental conditions such as salinity, high temperature, drought, light, and cold (Sairam et al., 2002; Prasad et al., 2008; Sunoj et al., 2017; Singh, 2023). However, the ultimate output of this study is to identify the tolerant and susceptible genotypes, which can be determined by grain yield. All other factors support and play a major role in achieving the final output, which is grain yield.

## 5 Conclusions

Salinity reduced all germination and early seedling traits in studied winter wheat genotypes, except germination and results indicate the existence of a wide range of genetic variation in salinity responses across the winter wheat genotype. Further evaluation on selected genotypes assumed to be tolerant (Gage, Guymon, MTS0531 and Tascosa) and suspectable (CO04W320, Carson, TX04M410211 and 2174–05) based on the SVI showed inconsistency in the magnitude of response of physiological and biochemical traits at subsequent booting stage and in agronomic traits. Based on the grain yield of selected genotypes exposed to high level of salinity at the booting stage, genotypes showed a mixed response, and genotype Guymon and TX04M410211 are found to be highly tolerant, and Gage and CO04W320 are highly susceptible. The results clearly suggest that the salinity screening of a large number of genotypes based on germination and early seedling traits needed to be reconfirmed with their response at other specific growth stages. Further, for an agro-climatic region-specific screening for salinity tolerant genotype, it is important to consider the mode of occurrence of salinity that determines the duration of exposure and intensity of sanity to determine suitable genotypes. However, genotypes identified in our study can be used for the developing biparental population with an aim to map the genomic regions responsible for the tolerance and susceptibility to salinity at germination to early seedling and the booting stages. The physiological and biochemical traits that showed a tradeoff should be studied thoroughly using contrasting genotypes and used for screening salinity-tolerant genotypes and future breeding programs.

## Data availability statement

The original contributions presented in the study are included in the article/**Supplementary Material**. Further inquiries can be directed to the corresponding authors.

## Author contributions

AE: Conceptualization, Data curation, Formal analysis, Investigation, Methodology, Writing – original draft, Writing – review & editing. VS: Conceptualization, Data curation, Formal analysis, Investigation, Methodology, Software, Supervision, Validation, Visualization, Writing – original draft, Writing – review & editing. MD: Writing – review & editing, Formal analysis. PP: Conceptualization, Data curation, Funding acquisition, Methodology, Project administration, Resources, Supervision, Validation, Visualization, Writing – review & editing.
