# Supplementary material for: Response of winter wheat genotypes to salinity stress under controlled environments
Source: Front Plant Sci. 2024 Jun 24;15:1396498. doi: 10.3389/fpls.2024.1396498 (PMC11228282; doi:10.3389/fpls.2024.1396498)
Supplement: Supplementary file 1 [file Table_1.docx]

Supplementary Table 1. List of genotypes in the hard winter wheat association mapping panel (HWWAMP) with year of release, type (C: cultivar, L: landrace, and B: breeding line), origin (USA [CO: Colorado, KS: Kansas, MT: Michigan, MO: Montana, NE: Nebraska, ND: North Dakota, OK: Oklahoma, SD: South Dakota, and TX: Texas] and Ukraine), National Society of Genetic Counselors (NSGC) accession number, and pedigree.

| Genotype | Year | Type | Origin | NSGC Accession | Pedigree |
| --- | --- | --- | --- | --- | --- |
| CO03064 | . | B | CO | . | CO970547/Prowers 99 |
| CO03W043 | . | B | CO | . | KS96HW94/CO980352 |
| CO03W054 | . | B | CO | . | Arlin/KS89H20 (KS96HW94)/6/Trego/5/(CO960293) PI 222668 / TAM 107 /4/(CO0850034) Novi Sad 14 / Novi Sad 603 // Newton /3/ Probrand 835 |
| CO04025 | . | B | CO | . | CO940610/CO960293//CO99W189 |
| CO04393 | . | B | CO | . | Stanton/CO950043 |
| CO04499 | . | B | CO | . | Above/Stanton |
| CO04W320 | . | B | CO | . | CO950635/CO99W1126 |
| CO050337-2 | . | B | CO | . | CO980829/TAM 111 |
| CO940610 | . | B | CO | GSTR10702 | H15A13333 /5* Larned // Eagle / Sage /3/ TAM 105 (KS87H22) /4/ (MW09) Clark's Cream/5*KS75216 (Newton Sib) |
| TAM107-R7 | . | B | CO | GSTR11601 | CO850034 / PI372129 //5* TAM107 |
| ABOVE | 2001 | C | CO | PI631449 | TAM 110*4/FS2 |
| AKRON | 1994 | C | CO | PI584504 | TAM 107 / Hail |
| ANTERO | 2013 | C | CO | PI 667743 | Trego/Betty sib (KS01HW152-1)//TAM 111 |
| AVALANCHE | 2001 | C | CO | PI620766 | RL6005 / RL6008 // Larned /3/ Cheney / Larned /4/ Bennett sib /5/ TAM107 (KS87H325) /6/ Rio Blanco |
| BILL BROWN | 2007 | C | CO | PI653260 | Yumar/Arlin |
| BOND CL | 2004 | C | CO | PI639924 | Yumar//TXGH12588-120*4/FS2 |
| BYRD | 2011 | C | CO | PI 664257 | TAM112//(CO970547-7) Ike/Halt |
| CARSON | 1986 | C | CO | PI501534 | Anza / Scout // Centurk |
| DAWN | 1982 | C | CO | CItr17801 | II 21031 / Trapper /4/(CO 652363) Warrior // Kenya 58 / Newthatch /2*( Cheyenne / Tenmarq / Mediterranean )/ Hope /3/ Parker |
| DENALI | 2011 | C | CO | PI 664256 | Yuma/T-57//CO850034/3/4*Yuma/4/NEWS12 (CO980829)/5/Tam 111 |
| DUKE | 1981 | C | CO | CItr17856 | 3* Sonora 64 / Warrior // Selkirk /2* Cheyenne /5/ Scout /4/ Quivera /3/ Tenmark // Marquis 1 / Oro |
| HAIL | 1982 | C | CO | PI470927 | Mexican / USA // Scout /3/ Mara /4/ Scout /5/ Ciano /6/ Trapper /7/ Parker |
| HALT | 1994 | C | CO | PI584505 | Sumner / CO820026 // PI372129 /3/ TAM 107 |
| HATCHER | 2004 | C | CO | PI638512 | Yuma / PI 372129 // TAM 200 /3/4* Yuma /4/ KS91H184 / Vista |
| JULES | 1993 | C | CO | PI564851 | Warrior *5/ Agent // Agate sib (NE76667)/3/ Hawk |
| LAMAR | 1988 | C | CO | PI559719 | 74 F878 ( Mexican dwarf )/ Wings // Vona |
| LINDON | 1975 | C | CO | CItr17440 | Andes 64A / Sonora 64 // Tacuari (II21183)/4/(CO 652363) Warrior 2 / Kenya 58 / Newthatch // Cheyenne / Tenmark / Mediterranean / Hope /3/ Parker/5/ Lancer /3/(KS 62136) Norin 16 / CI 12500 // Kaw |
| OGALLALA | 1993 | C | CO | PI573037 | TX81V6187 / Abilene |
| PRAIRIE RED | 2000 | C | CO | PI605390 | CO850034 / PI 372129 //5* TAM 107 |
| PROWERS | 1997 | C | CO | PI605389 | CO850060 / PI 372129 //5* Lamar |
| RIPPER | 2006 | C | CO | PI644222 | PI 220127/P5//TAM-200/KS87H66 (CO940606)/3/(TAM107R-2) CO850034/PI 372129//5*TAM 107 |
| SANDY | 1981 | C | CO | CItr17857 | Sonora 64A / Tezanos Pintos Precoz / Yaqui 54 //( Frontana / Kenya 58 / Newthatch )/ Norin 10 / Brevor / Gabo 55B / Trapper // Centurk |
| THUNDER CL | 2008 | C | CO | PI655528 | FS2/KS97HW150//KS97HW349 (KS01-5539)/3/(CO99W165) KS92WGRC25/Halt |
| VONA | 1976 | C | CO | CItr17441 | Andes 64A / Sonora 64 // Tacuari (II 21183) /4/ (CO 652363) Warrior // Kenya 58 / Newthatch /2*( Cheyenne / Tenmarq / Mediterranean / Hope /3/ Parker /5/ Lancer /4/ KS 62136 |
| YUMA | 1992 | C | CO | PI559720 | NS14 / NS25 //2* Vona |
| YUMAR | 2000 | C | CO | PI605388 | Yuma / PI 372129 , F1 // CO850034 /3/4* Yuma |
| HV906-865 | . | B | KS | . | G980039/Onaga |
| HV9W03-1379R | . | B | KS | . | B1127/3/B1551W//ROWDY/RWA 671 MONT |
| HV9W03-1551WP | . | B | KS | . | B1043/PL2180 |
| HV9W03-1596R | . | B | KS | . | B1397-1/WGRC33 |
| HV9W05-1280R | . | B | KS | . | SPARTANKA/G980761 |
| HV9W06-504 | . | B | KS | . | G982231/G982159//KS920709W |
| KS00F5-20-3 | . | B | KS | . | 0 |
| W04-417 | . | B | KS | . | BULK POPULATION |
| WB411W | . | B | KS | . | G3006/ARLIN |
| 2145 | 2002 | C | KS | PI 631087 | HBA142A/HBZ621A//Abilene |
| 2180 | 1989 | C | KS | PI532912 | TAM W-101 / Pioneer W603 // Pioneer W558 |
| ARLIN | 1992 | C | KS | PI564246 | Selection from population of intercrossed hard red winter wheat and hard red spring wheat genotypes |
| BAKER'S WHITE | 2004 | C | KS | PI 633865 | Ponderosa/Jagger |
| BISON | 1956 | C | KS | CItr12518 | Chiefkan // Oro / Tenmarq |
| BURCHETT | 2004 | C | KS | PI 633863 | W91-126/WI88-052-05 |
| CHENEY | 1978 | C | KS | CItr17765 | Scout / Tascosa |
| COMANCHE | 1942 | C | KS | CItr11673 | Oro / Tenmarq |
| COSSACK | 1998 | C | KS | PI 606780 | BCD1828/83 |
| CUTTER | 2002 | C | KS | PI 631389 | JAGGER//(WI89-189-14)Tam200/Stallion sib |
| DANBY | 2007 | C | KS | PI 648010 | Trego/Jagger 'S' |
| DODGE | 1986 | C | KS | PI506344 | KS73H530 ( Newton sib )/ KS76HN1978-1 ( Arkan sib ) |
| DUMAS | 2001 | C | KS | PI 619199 | WI90-425/WI89-483 |
| EAGLE | 1970 | C | KS | CItr15068 | Selection from Scout |
| ENHANCER | 1998 | C | KS | PI606779 | 1992 Nebraska bulk selection |
| FULLER | 2007 | C | KS | PI 653521 | Ogallala/KS95WGRC33//Jagger |
| G1878 | 1995 | C | KS | PI 591622 | Hawk/Sturdy//Plainsman V |
| HEYNE | 2001 | C | KS | PI612577 | KS82W422 / SWM754308 / KS831182 / KS82W422 |
| HONDO | 1999 | C | KS | PI 603958 | W84-179/W81-171/5/Sturdy/Hawk/4/Vona/3/NDD63/CO652643//Centurk |
| JAGALENE | 2002 | C | KS | PI 631376 | JAGGER/ABILENE |
| JAGGER | 1994 | C | KS | PI593688 | KS82W418 / Stephens |
| KARL 92 | 1992 | C | KS | PI564245 | Selection from Karl = Plainsman V /3/ Kaw / Atlas 50 // Parker *5/ Agent |
| KAW61 | 1960 | C | KS | CItr12871 | purification and re - release of Kaw = Oro // Mediterranean / Hope /3/ Early Blackhull / Tenmarq |
| KEOTA | 2007 | C | KS | PI 648007 | CUSTER/JAGGER |
| KIOWA | 1950 | C | KS | CItr12133 | Chiefkan // Oro / Tenmarq |
| KIRWIN | 1973 | C | KS | CItr17275 | Parker *3/ Bison |
| LAKIN | 2002 | C | KS | PI617032 | KS89H130 / Arlin |
| LARNED | 1976 | C | KS | CItr17650 | Ottawa /5* Scout |
| LONGHORN | 1991 | C | KS | PI552813 | NS2630-1 / Thunderbird |
| NEOSHO | 2006 | C | KS | PI 639739 | W91-376-20/W95-084 |
| NEWTON | 1978 | C | KS | CItr17715 | Pitic 62 / Chris sib //2* Sonora 64 /3/ Klein Rendidor /4/ Scout |
| NORKAN | 1986 | C | KS | PI506345 | Plainsman V /3/2*( KS76H3705 ) Larned / Eagle // Sage |
| NUFRONTIER | 2002 | C | KS | PI 619089 | 2180/HBZ356A//Mesa |
| NUHORIZON | 2001 | C | KS | PI 619198 | WI89-282/Arlin |
| ONAGA | 1998 | C | KS | . | HT43-231-19 (Pioneer bulk) |
| OVERLEY | 2004 | C | KS | PI 634974 | TAM-107 *3/TA 2460 (U1275-1-4-2-2)//Heyne ‘S’/3/Jagger |
| PARKER | 1966 | C | KS | CItr13285 | Quivira /3/ Kanred / Hard Federation // Prelude / Kanred /4/ Kawvale / Marquillo // Kawvale / Tenmarq |
| PARKER 76 | 1976 | C | KS | CItr17685 | Parker *5/ Agent |
| PLATTE | 1997 | C | KS | PI 596297 | Tesia 79 / Chat'S' // Abilene |
| POSTROCK | 2006 | C | KS | PI 643093 | Ogallala/KSU94U261//Jagger |
| RONL | 2007 | C | KS | PI 648020 | Trego/3/(CO9600293) PI222668/TAM 107//CO850034 |
| SAGE | 1973 | C | KS | CItr17277 | Agent /4* Scout |
| SANTA FE | 2006 | C | KS | PI 641772 | G1878/Jagger |
| SHAWNEE | 1967 | C | KS | CItr14157 | Mediterranean / Hope // Pawnee /3/ Oro / Illinois No. 1// Comanche |
| SHOCKER | 2006 | C | KS | PI 646185 | FREEDOM/TOMAHAWK//JAGGER |
| SMOKYHILL | 2006 | C | KS | PI 646184 | 97 8/64 MASA (Population developed by combining several crosses with a common female "G2500") |
| SPARTAN | 2007 | C | KS | . | RL8400193/PL2180 |
| STANTON | 2002 | C | KS | PI617033 | PI 220350 / KS87H57 // TAM200 / KS87H66 /3/ KS87H325 |
| TARKIO | 2006 | C | KS | . | OK90604/KSSB-369-7//SnowWhite |
| THUNDERBOLT | 2000 | C | KS | PI 608000 | ABILENE/KS90WGRC10 |
| TREGO | 1999 | C | KS | PI612576 | RL6005 / RL6008 // Larned /3/ Cheney / Larned /4/ Bennet sib /5/ TAM107 (KS87H325)/6/ Rio Blanco |
| TRISON | 1973 | C | KS | CItr17278 | Triumph / Bison |
| VENANGO | 2000 | C | KS | . | HBE1066-105/HBF0551-137 |
| WICHITA | 1944 | C | KS | CItr11952 | Early Blackhull / Tenmarq |
| WICHITA | 1944 | C | KS | CItr11952 | Early Blackhull / Tenmarq |
| E2041 | . | B | MI | . | Pioneer Brand 2552/Pioneer Brand 2737W |
| MT0495 | . | B | MT | . | MT9640/NB1133 |
| MT06103 | . | B | MT | . | Composite cross |
| MT85200 | . | B | MT | . | Froid/Winoka/3/TX55-391-56-D8/Westmont//Trader |
| MT9513 | . | B | MT | . | NuWest/MT8030 |
| MT9904 | . | B | MT | . | MT85200/Tiber |
| MT9982 | . | B | MT | . | Promontory/Judith |
| MTS0531 | . | B | MT | . | L'Govskaya167/Rampart//MT9409 |
| BIG SKY | 2001 | C | MT | PI619166 | NuWest / Tiber |
| CREST | 1967 | C | MT | CItr13880 | Westmont *2/ PI 178383 |
| DECADE | 2010 | C | MT | PI660291 | Composite |
| GENOU | 2004 | C | MT | PI640424 | Lew/Tiber//Redwin (MTS92015)/3/Vanguard/Norstar |
| JUDEE | 2011 | C | MT | PI 665227 | Vanguard/Norstar//Judith/3/NuHorizon |
| JUDITH | 1989 | C | MT | PI584526 | Lancota / Froid // NE69559 / Winoka |
| NORRIS | 2005 | C | MT | PI643430 | BigSky//TAM110sib*4/FS2 |
| NUSKY | 2001 | C | MT | PI619167 | NuWest / Tiber |
| ROSEBUD | 1981 | C | MT | PI473570 | Lancer /2* BWH 1376-8 |
| YELLOWSTONE | 2005 | C | MT | PI643428 | Selected from a composite of F2 seed from two closely related populations: Promontory/Judith and Judith-phenotypic dwarf selection/Promontory |
| JERRY | 2001 | C | ND | PI632433 | Roughrider // Winoka / NB66425 /3/ Arapahoe |
| NE02558 | . | B | NE | . | JAGGER/ALLIANCE |
| NE04490 | . | B | NE | . | NE95589/3/(NE94632) ABILENE/NORKAN//RAWHIDE/4/(NE95510)ABILENE/ARAPAHOE |
| NE05430 | . | B | NE | . | IN92823A1-1-4-5/NE92458 |
| NE05496 | . | B | NE | . | KS87H325/RIO BLANCO (KS95HW62-6)//HALLAM |
| NE06607 | . | B | NE | . | KS89H50-4/3/(NE90518)BRL//SXL/BENN (NE98466)/4/WESLEY |
| NE99495 | . | B | NE | . | ALLIANCE/KARL 92 |
| NI06736 | . | B | NE | . | KM602-90/NE89657//ARLIN (NW97S312)/3/(KS96HW10-3) KS91HW29// RIO BLANCO/KS91H184 |
| NI06737 | . | B | NE | . | KM602-90/NE89657//ARLIN (NW97S312)/3/(KS96HW10-3) KS91HW29// RIO BLANCO/KS91H184 |
| NI07703 | . | B | NE | . | 919021/B725//K92 (G97343, R-148)/5/(NI00436) BEZ 1/CTK78//ARTHUR/CTK78/3/BENNET/4/NORKAN |
| NI08707 | . | B | NE | . | Yuma/T-57//CO850034/3/4*Yuma/4/NEWS1 (CO980829)/5/Wesley |
| NI08708 | . | B | NE | . | Yuma/T-57//CO850034/3/4*Yuma/4/NEWS1 (CO980829)/5/Wesley |
| NW03666 | . | B | NE | . | N94S097KS/NE93459 |
| AGATE | 1979 | C | NE | CI17463 | Ponca /3* Cheyenne // Kenya 58 / Newthatch //2*( Cheyenne / Tenmarq / Mediterranean / Hope )/3/ Scout |
| ALLIANCE | 1993 | C | NE | PI573096 | Arkan/Colt//Chisholm (sib) |
| ANTELOPE | 2005 | C | NE | PI633910 | Pronghorn / Arlin |
| ANTON | 2007 | C | NE | PI651044 | WA691213-27 / PI 559717 // Platte |
| ARAPAHOE | 1988 | C | NE | PI518591 | Brule /3/ Parker *4/ Agent // Belocerkovskaja 198 / Lancer |
| BENNETT | 1978 | C | NE | CI17723 | Scout /3/ Quivira / Tenmarq // Marquillo / Oro /4/ Homestead |
| BUCKSKIN | 1973 | C | NE | CI17263 | Scout/3/Quivera/Tenmarq//Marquillo/Oro |
| CAMELOT | 2008 | C | NE | PI653832 | KS91H184/ARLIN SIB//KS91HW29/3/NE82761/REDLAND (NE91631)//VBFO168 |
| CENTURA | 1983 | C | NE | PI476974 | Warrior*5/Agent/NE68457/3/Centurk78 |
| CENTURK 78 | 1978 | C | NE | CItr17724 | Selection from Centurk |
| COLT | 1983 | C | NE | PI476975 | Agate sib ( NE69441 )// ( Tx65A1503-1 ) 391-56-D8 / Kaw |
| COUGAR | 2000 | C | NE | PI613098 | Warrior *5/ Agent // Kavkaz /4/ NE63218 / Kenya 58 /3/ Newthatch /2* CTMH // Ponca /* 2 Cheyenne (NE85707)/5/ Thunderbird ( CTMH = Cheyenne / Tenmarq / Mediterranean / Hope ) |
| CULVER | 1999 | C | NE | PI606726 | NE82419/Arapahoe |
| FREEMAN | 2013 | C | NE | PI 667038 | ABI86*3414/Jagger//Karl 92 (KS92-946-B-15-1)/3/ALLIANCE |
| GAGE | 1963 | C | NE | CItr13532 | Ponca /3/ Mediterranean / Hope // Pawnee |
| GOODSTREAK | 2002 | C | NE | PI632434 | Len // Butte / ND526 (ND604) /6/ (SD2971) Agent /3/ ND441 // Waldron / Bluebird /4/ Butte /5/ Len (SD3055) /7/ KS88H164 /8/ NE89646 |
| HALLAM | 2006 | C | NE | PI638790 | Brule / Bennett // Niobrara |
| HARRY | 2002 | C | NE | PI632435 | Brule /4/ Parker *4/ Agent // Beloterkovskaia 198 / Lancer /3/ Newton / Brule (NE90614) /5/ (NE87612) Newton // Warrior *5/ Agent /3/ Agate sib |
| HOMESTEAD | 1973 | C | NE | CI17264 | Scout /4/ Kenya / Newthatch // Cheyenne / Tenmarq / Mediterranean / Hope /3/ Pawnee / Cheyenne |
| INFINITY CL | 2006 | C | NE | PI639922 | Windstar//Millennium sib/Above sib |
| LANCER | 1963 | C | NE | CItr13547 | Turkey Red / Cheyenne // Hope /2* Cheyenne |
| MACE | 2007 | C | NE | PI651043 | Yuma//PI 372129/3/CO850034/4/4*Yuma/5/KS91H184/Arlin S//KS91HW29/3/NE89526 |
| MCGILL | 2010 | C | NE | PI659689 | Vona // Chisholm / PlainsmanV (OK83201)/3/Redland (NE92458 )/4/ Ike |
| MILLENNIUM | 2000 | C | NE | PI613099 | Arapahoe / Abilene /4/ Colt /3/ Warrior *5/ Agent // Kavkaz |
| NEKOTA | 1994 | C | NE | PI584997 | Bennett/TAM 107 |
| NIOBRARA | 1994 | C | NE | PI584996 | TAM 105*5/AMIGO//Brule |
| NUPLAINS | 1998 | C | NE | PI605741 | Abilene / KS831872 = Abilene /3/ Plainsman V // Newton / Arthur 71 |
| OVERLAND | 2007 | C | NE | PI647959 | Millennium sib//(ND8974) Seward/Archer |
| PANHANDLE | 2014 | C | NE | . | BRIGANTINA/2*ARAPAHOE (NE97426)//NE98574 |
| PRONGHORN | 1996 | C | NE | PI593047 | Centura/Dawn//Colt |
| RAWHIDE | 1990 | C | NE | PI543893 | Warrior *5/ Agent // Kavkaz /4/ Parker *4/ Agent // Belocerkovskaja 198 / Lancer /3/ Vona |
| REDLAND | 1986 | C | NE | PI502907 | Selection from Brule |
| ROBIDOUX | 2010 | C | NE | PI659690 | Odesskaya P / Cody // Pavon 76 /3* Scout 66 (NE96644)/3/ Wahoo sib |
| SCOUT 66 | 1967 | C | NE | CI13996 | composite of 85 selections from Scout, CItr 13546 (Scout = Nebred // Hope / Turkey /3/ Cheyenne / Ponca) |
| SETTLER CL | 2009 | C | NE | PI653833 | Wesley sib // Millennium sib / Above sib |
| SIOUXLAND | 1984 | C | NE | PI483469 | Warrior*5/Agent*2//Kavkaz |
| VISTA | 1992 | C | NE | PI562653 | Warrior // Atlas 66 / Comanche /3/ Comanche / Ottawa (NE68513)/5/(NE68457) Ponca /2* Cheyenne /3/ Illinois No. 1//2* Chinese Spring /T. timopheevii /4/ Cheyenne / Tenmarq // Mediterranean / Hope /3/ Sando 60 /6/ Centurk / Brule |
| WAHOO | 2000 | C | NE | PI619098 | Arapahoe *2/ Abilene |
| WARRIOR | 1960 | C | NE | CItr13190 | Pawnee / Cheyenne |
| WESLEY | 1998 | C | NE | PI605742 | KS831936-3 / NE86501 = Sumner sib ( Plainsman V / Odesskaya 51 )// Colt / Cody |
| WINDSTAR | 1996 | C | NE | PI597379 | TAM103 / Newton sib (TX79A2729)// Caldwell / Brule field sel .6/3/ Siouxland |
| CHEYENNE | 1933 | L | NE | CI8885 | selection from Crimean, CI 1435 |
| TURKEY | 1874 | L | NE | CI 12137 | The original Turkey (Nebr. No. 1) grown at Lincoln since 1897. From it were selected Nebr. 6, 60, etc. |
| OK02405 | . | B | OK | . | Tonkawa/GK50 |
| OK04111 | . | B | OK | . | 2174*2/Jagger |
| OK04415 | . | B | OK | . | N563/OK98G508W |
| OK04505 | . | B | OK | . | OK91724/2*Jagger |
| OK04507 | . | B | OK | . | OK95593/Jagger//2174 |
| OK04525 | . | B | OK | . | FFR525W/Hickok//Coronado |
| OK05108 | . | B | OK | . | Lut 13686/2174//Jagger |
| OK05122 | . | B | OK | . | KS94U337/NE93427 |
| OK05134 | . | B | OK | . | OK97411/TX91D6825 |
| OK05204 | . | B | OK | . | SWM866442/OK95548 |
| OK05303 | . | B | OK | . | OK95548/TXHBG0358 |
| OK05312 | . | B | OK | . | TX93V5919/WGRC40//OK94P549/WGRC34 |
| OK05511 | . | B | OK | . | TAM 110/2174 |
| OK05711W | . | B | OK | . | G1878/OK98G508W |
| OK05723W | . | B | OK | . | SWM866442/Betty |
| OK05830 | . | B | OK | . | OK93617/Jagger |
| OK06114 | . | B | OK | . | KS97P0630-4-5/CM95560//X920879-C15-1/3/X84WO63-9-18/U1324-25-1-4 |
| OK06210 | . | B | OK | . | KS90175-1-2/CMSW89Y271//K92/3/ABI 86*3414/X86035*-BB-34//HBC 302E |
| OK06318 | . | B | OK | . | HBG0358/2174//2145 |
| OK06319 | . | B | OK | . | Enhancer/2174 |
| OK06336 | . | B | OK | . | Magvars/2174//Enhancer |
| OK07231 | . | B | OK | . | OK92P577-RMH 3099/Duster |
| OK07S117 | . | B | OK | . | [ALTAR84/AE.SQ//OPATA]/OK98G508W |
| OK08328 | . | B | OK | . | GK Keve/Ok101//OK93P656-RMH3299 |
| OK09634 | . | B | OK | . | OK95616-98-6756/Overley |
| OK10119 | . | B | OK | . | JEI 110/Overley |
| OK1067071 | . | B | OK | . | TX98V9437/OK00316//Farmec |
| OK1067274 | . | B | OK | . | GA961912-8-4-5/OK02129//Kristi-K.K |
| OK1068002 | . | B | OK | . | EFECT/Jagalene//Deliver |
| OK1068009 | . | B | OK | . | LADA/Jagalene//G980122 |
| OK1068026 | . | B | OK | . | ERYTHROSPERMUM 270/TAM 111//OK99212 |
| OK1068112 | . | B | OK | . | Farmec/Jagalene |
| OK1070267 | . | B | OK | . | VI.9/Guymon//G980411W |
| OK1070275 | . | B | OK | . | KNJAZHNA/KS00HW175-4//OK00611W |
| 2174-05 | 1998 | C | OK | PI602595 | IL71-5662/PL145(Newton sib)//2165 |
| BILLINGS | 2009 | C | OK | PI656843 | N566/OK94P597 |
| CENTERFIELD | 2006 | C | OK | PI644017 | TXGH12588-105*4/FS4//2*2174 |
| CENTURY | 1986 | C | OK | PI502912 | Payne // TAM W-101 / Amigo |
| CHISHOLM | 1983 | C | OK | PI486219 | Sturdy sib / Nicoma |
| CUSTER | 1994 | C | OK | . | F-29-76/TAM-105//Chisholm |
| DELIVER | 2004 | C | OK | PI639232 | Yantar/2*Chisholm (OK91724)//Karl |
| DUSTER | 2006 | C | OK | PI644016 | W0405D/NE78488//W7469C/TX81V6187 |
| ENDURANCE | 2004 | C | OK | PI639233 | HBY756A/Siouxland//2180 |
| GALLAGHER | 2013 | C | OK | PI 667569 | OK99711/Duster |
| GARRISON | 2011 | C | OK | PI661992 | OK95616-1/Hickok//Betty |
| GUYMON | 2005 | C | OK | PI643133 | Intrada/Platte |
| INTRADA | 2000 | C | OK | PI631402 | Rio Blanco / TAM 200 |
| OK BULLET | 2005 | C | OK | PI642415 | KS96WGRC39/Jagger |
| OK RISING | 2009 | C | OK | PI656382 | KS96WGRC39/Jagger |
| OK101 | 2001 | C | OK | PI631493 | OK87W663/Mesa//2180 |
| OK102 | 2002 | C | OK | PI632635 | 2174/Cimarron |
| PETE | 2009 | C | OK | PI656844 | N40/OK94P455 |
| RUBY LEE | 2011 | C | OK | PI661991 | KS94U275/OK94P549 |
| TRIUMPH 64 | 1964 | C | OK | CItr13679 | Danne Beardless Blackhull /3/ Kanred / Blackhull // Florence /4/ Kanred / Blackhull // Triumph |
| SD01058 | . | B | SD | . | XH1877/NE967430 |
| SD01237 | . | B | SD | . | UNKNOWN |
| SD05118 | . | B | SD | . | Wesley/NE93613 |
| SD05210 | . | B | SD | . | SD98444/SD97060 |
| SD05W018 | . | B | SD | . | SD98W302/SD98W175 |
| ALICE | 2006 | C | SD | PI644223 | Abilene/Karl. |
| BRONZE | 1974 | C | SD | CItr14013 | Hume / Gage /4/ Hume /3/ NE61943 , Mida / Kenya 117A //2* Hope /2* Turkey Red |
| CRIMSON | 1997 | C | SD | PI601818 | TAM-105 / Winoka |
| DARRELL | 2006 | C | SD | PI644224 | 2076-W12-11/Karl92 |
| EXPEDITION | 2002 | C | SD | PI629060 | Tomahawk / Bennett |
| GENT | 1974 | C | SD | CItr17293 | Agent /4* Scout |
| HARDING | 1999 | C | SD | PI608049 | Brule // Bennett / Chisholm /3/ Arapahoe |
| HUME | 1965 | C | SD | CItr13526 | crosses involving: Minter, Kharkof, Wichita, Nebred, Cheyenne, and others |
| LYMAN | 2009 | C | SD | PI 658067 | KS93U134/Arapahoe |
| NELL | 1981 | C | SD | CItr17803 | Scout selection / Capitan |
| RITA | 1980 | C | SD | CItr17799 | Seu Seun / Denton 8 // Westmont /3/ (SD 6689) Ponca //3* Cheyenne / Kenya58 / Newthatch //2*( Cheyenne / Tenmarq // Mediterranean / Hope ) |
| ROSE | 1979 | C | SD | CItr17795 | Seu Seun / Denton 8 // Westmont /4/ Hume /3/ NE 63265 |
| TANDEM | 1997 | C | SD | PI601817 | Brule / Agate |
| WENDY | 2004 | C | SD | PI638521 | Gent/Siouxland (SD89333) // Abilene |
| WINOKA | 1969 | C | SD | CItr14000 | Selection from Winalta |
| TX00V1131 | . | B | TX | . | TX87V1613/KS91WGRC11 |
| TX01A5936 | . | B | TX | . | JAGGER/3/PSN 'S'/BOW 'S'//T200 |
| TX01M5009-28 | . | B | TX | . | MASON/JAGGER//PECOS |
| TX01V5134RC-3 | . | B | TX | . | TAM-200/JAGGER |
| TX03A0148 | . | B | TX | . | TX89A7137/TIPACNA |
| TX03A0563 | . | B | TX | . | X96V107/OGALLALA |
| TX04A001246 | . | B | TX | . | TX95V4339/TX94VT938-6 |
| TX04M410164 | . | B | TX | . | MIT/TX93V5722//W95-301 |
| TX04M410211 | . | B | TX | . | MASON/JAGGER//OGALLALA |
| TX04V075080 | . | B | TX | . | JAGGER/TX93V5722//TX95D8905 |
| TX05A001188 | . | B | TX | . | T107//TX98V3620/Ctk78/3/TX87V1233/4/N87V106//TX86V1540/T200 |
| TX05A001822 | . | B | TX | . | 2145/X940786-6-7 |
| TX05V7259 | . | B | TX | . | T107//TX78V3620/Ctk78/3/TX87V1233/4/Arap//TX86V1540/T200 |
| TX05V7269 | . | B | TX | . | HBG0358/4/T107//TX78V3620/Ctk78/3/TX87V1233 |
| TX06A001132 | . | B | TX | . | HBG0358/4/T107//TX78V3620/Ctk78/3/TX87V1233 |
| TX06A001263 | . | B | TX | . | UNKNOWN |
| TX06A001281 | . | B | TX | . | TX98VR8422/U3704A-7-7 |
| TX06A001386 | . | B | TX | . | TX99A6030/CUSTER |
| TX06V7266 | . | B | TX | . | TX99U8617/TX97U2001 |
| TX07A001279 | . | B | TX | . | X930332-4-1/TX97V2838 |
| TX07A001318 | . | B | TX | . | TX98VR8431/TX95A3091 |
| TX07A001420 | . | B | TX | . | U1254-1-5-2-1/TX81V6582//DESCONOCIDO |
| TX86A5606 | . | B | TX | . | TAM 105*4/AMI*4//LGO |
| TX86A6880 | . | B | TX | . | TAM 105*4/AMI*4//LGO |
| TX86A8072 | . | B | TX | . | TAM 105*4/AMI*4//LGO |
| TX96D1073 | . | B | TX | . | TX86D1310/Kavkaz//TX86D1308 (=WX87D144-10-99-12-18) |
| TX99A0153-1 | . | B | TX | . | OGALLALA/TAM-202 |
| TX99U8618 | . | B | TX | . | TX84V1237/TX71C8130R |
| CAPROCK | 1969 | C | TX | CItr14516 | Sinvalocho / Wichita // Hope / Cheyenne /3/ Wichita /4/ Seu Seun 27 |
| HG-9 | 2000 | C | TX | PI614118 | TAM 200 outcross selection |
| LOCKETT | 2001 | C | TX | PI604245 | TX86V1540 / TX78V2430-4 |
| MIT | 1980 | C | TX | CItr17896 | Sinvalocho / Wichita // Hope / Cheyenne /3/ Wichita /4/ Seu Seun 27 (TX391-56-D1 - 24)/6/T. dicoccoides / Aeg. speltoides , amphiploid //2* Austin /3/ Supremo (TX55C907)/4/ Bison /5/ Caddo/7/ Frontana / Westar |
| STURDY | 1966 | C | TX | CItr13684 | Sinvalocho / Wichita // Hope / Cheyenne /3/2* Wichita /4/ Seu Seun 27 |
| STURDY 2K | 2005 | C | TX | PI636307 | Sturdy Resel. |
| TAM 105 | 1979 | C | TX | CItr17826 | ' short wheat' / Sturdy composite bulk selection |
| TAM 107 | 1984 | C | TX | PI495594 | TAM 105 *4/ Amigo |
| TAM 109 | 1991 | C | TX | PI554606 | TAMW-101 *5/ CI9321 |
| TAM 110 | 1996 | C | TX | PI595757 | TAM 107*5/Largo |
| TAM 111 | 2002 | C | TX | PI631352 | TAM 107 // TX78V3630 / Centurk 78 /3/ TX87V1233 = TAM 107 /4/ Sturdy sib / Kaw // Centurk /3/ Centurk 78 /5/ Sturdy sub / Kaw // Centurk /3/ Jupetaco / Bluejay |
| TAM 112 | 2007 | C | TX | PI643143 | TAM 200/TA2460 (U1254-7-9-2-1)//(TXGH10440) TAM 107*5/Largo |
| TAM 113 | 2013 | C | TX | PI 666125 | TX90V6313/TX94V3724 |
| TAM 200 | 1986 | C | TX | PI578255 | Sturdy sib / Tascosa // Centurk *3/3/ Amigo |
| TAM 202 | 1992 | C | TX | PI561933 | Siouxland outcross |
| TAM 203 | 2009 | C | TX | PI655960 | TX89V4132/704 L I-2221 |
| TAM 302 | 1998 | C | TX | PI605910 | Probrand 812 / Caldwell // (TX86D1310) TAM300 sib |
| TAM 303 | 2006 | C | TX | . | TX89D1253*2/TTCC404 (=WX93D208-9-1-2) |
| TAM 304 | 2009 | C | TX | PI655234 | TX92U3060/TX91D6564 |
| TAM 400 | 2001 | C | TX | PI614876 | TAM-200//(TX82D5668) Era/TAMW-101 |
| TAM 401 | 2010 | C | TX | PI658500 | Mason/Jagger |
| TAM W-101 | 1971 | C | TX | CItr15324 | Norin 10 /3/ Nebraska 60 // Mediterranean / Hope /4/ Bison |
| TASCOSA | 1959 | C | TX | CItr13023 | Kanred / Hard Federation // Tenmarq /3/ Mediterranean / Hope /4/ Cimarron |
| KHARKOF | 1900 | L | Ukraine | PI5641 | KHARKOF |
