# Supplementary material for: Response of winter wheat genotypes to salinity stress under controlled environments
Source: Front Plant Sci. 2024 Jun 24;15:1396498. doi: 10.3389/fpls.2024.1396498 (PMC11228282; doi:10.3389/fpls.2024.1396498)
Supplement: Supplementary file 2 [file Table_2.docx]

Supplementary Table 2: The mean values for germination % (G%), germination index (GI), mean daily germination (MDG), and germination rate (GR) of 292 genotypes from the hard winter wheat association mapping panel (HWWAMP) exposed control treatment (0 mM NaCl) and different levels of salinity (60, and 120 mM NaCl). *standard deviation.

| Genotypes | Salinity level | G % | Std Dev* | GI | Std Dev | MDG | Std Dev | GR | Std Dev |
| --- | --- | --- | --- | --- | --- | --- | --- | --- | --- |
| TRIUMPH64 | 0 | 98.8 | 2.5 | 100 | 0 | 6.6 | 0.17 | 2.2 | 0.01 |
| CHISHOLM | 0 | 97.5 | 2.9 | 100 | 0 | 6.5 | 0.19 | 2.2 | 0.04 |
| CUSTER | 0 | 96.3 | 4.8 | 100 | 0 | 6.4 | 0.32 | 2.1 | 0.05 |
| 2174-05 | 0 | 98.8 | 2.5 | 100 | 0 | 7.4 | 1.42 | 2.1 | 0.05 |
| INTRADA | 0 | 98.8 | 2.5 | 100 | 0 | 6.6 | 0.17 | 2.2 | 0.03 |
| OK101 | 0 | 97.5 | 2.9 | 100 | 0 | 6.5 | 0.19 | 2.2 | 0.04 |
| OK102 | 0 | 98.8 | 2.5 | 100 | 0 | 6.6 | 0.17 | 2.2 | 0.04 |
| ENDURANCE | 0 | 96.3 | 4.8 | 100 | 0 | 6.4 | 0.32 | 2.1 | 0.03 |
| DELIVER | 0 | 97.5 | 5.0 | 100 | 0 | 6.5 | 0.33 | 2.1 | 0.03 |
| OK_BULLET | 0 | 100.0 | 0.0 | 100 | 0 | 6.7 | 0.00 | 2.1 | 0.04 |
| CENTERFIELD | 0 | 98.8 | 2.5 | 100 | 0 | 6.2 | 0.96 | 2.2 | 0.05 |
| GUYMON | 0 | 98.8 | 2.5 | 100 | 0 | 8.2 | 1.79 | 2.0 | 0.03 |
| DUSTER | 0 | 97.5 | 5.0 | 100 | 0 | 6.5 | 0.33 | 2.2 | 0.03 |
| OK_RISING | 0 | 100.0 | 0.0 | 100 | 0 | 6.7 | 0.00 | 2.1 | 0.03 |
| OK02405 | 0 | 98.8 | 2.5 | 100 | 0 | 6.6 | 0.17 | 2.2 | 0.05 |
| PETE | 0 | 98.8 | 2.5 | 100 | 0 | 6.6 | 0.17 | 2.1 | 0.03 |
| BILLINGS | 0 | 96.3 | 7.5 | 100 | 0 | 6.4 | 0.50 | 2.1 | 0.04 |
| OK04505 | 0 | 100.0 | 0.0 | 100 | 0 | 6.7 | 0.00 | 2.2 | 0.04 |
| OK04525 | 0 | 100.0 | 0.0 | 100 | 0 | 6.7 | 0.00 | 2.1 | 0.03 |
| OK04507 | 0 | 100.0 | 0.0 | 100 | 0 | 6.7 | 0.00 | 2.2 | 0.03 |
| OK05830 | 0 | 97.5 | 5.0 | 100 | 0 | 6.5 | 0.33 | 2.1 | 0.03 |
| OK04111 | 0 | 98.8 | 2.5 | 100 | 0 | 6.6 | 0.17 | 2.1 | 0.05 |
| OK04415 | 0 | 100.0 | 0.0 | 100 | 0 | 6.7 | 0.00 | 2.1 | 0.03 |
| OK05711W | 0 | 97.5 | 5.0 | 100 | 0 | 6.5 | 0.33 | 2.2 | 0.06 |
| OK05723W | 0 | 100.0 | 0.0 | 100 | 0 | 6.7 | 0.00 | 2.1 | 0.03 |
| OK05108 | 0 | 98.8 | 2.5 | 100 | 0 | 7.4 | 1.42 | 2.1 | 0.06 |
| OK05122 | 0 | 97.5 | 5.0 | 100 | 0 | 6.5 | 0.33 | 2.1 | 0.02 |
| OK05526 | 0 | 100.0 | 0.0 | 100 | 0 | 6.7 | 0.00 | 2.2 | 0.04 |
| OK05134 | 0 | 98.8 | 2.5 | 100 | 0 | 6.2 | 0.79 | 2.2 | 0.06 |
| OK05303 | 0 | 98.8 | 2.5 | 100 | 0 | 6.2 | 0.79 | 2.2 | 0.03 |
| OK05312 | 0 | 98.8 | 2.5 | 100 | 0 | 6.2 | 0.79 | 2.2 | 0.05 |
| OK05511 | 0 | 98.8 | 2.5 | 100 | 0 | 6.2 | 0.79 | 2.2 | 0.04 |
| OK05204 | 0 | 98.8 | 2.5 | 100 | 0 | 6.2 | 0.79 | 2.2 | 0.04 |
| GARRISON | 0 | 97.5 | 5.0 | 100 | 0 | 6.5 | 0.33 | 2.1 | 0.03 |
| OK06114 | 0 | 96.3 | 7.5 | 100 | 0 | 6.4 | 0.50 | 2.2 | 0.04 |
| OK06210 | 0 | 100.0 | 0.0 | 100 | 0 | 6.3 | 0.83 | 2.2 | 0.06 |
| OK06319 | 0 | 98.8 | 2.5 | 100 | 0 | 6.6 | 0.17 | 2.2 | 0.06 |
| OK06318 | 0 | 100.0 | 0.0 | 100 | 0 | 6.7 | 0.00 | 2.2 | 0.03 |
| OK06336 | 0 | 97.5 | 5.0 | 100 | 0 | 6.5 | 0.33 | 2.2 | 0.02 |
| AGATE | 0 | 98.8 | 2.5 | 100 | 0 | 6.6 | 0.17 | 2.2 | 0.00 |
| ALLIANCE | 0 | 98.8 | 2.5 | 100 | 0 | 6.2 | 0.79 | 2.2 | 0.04 |
| ANTELOPE | 0 | 98.8 | 2.5 | 100 | 0 | 6.2 | 0.79 | 2.2 | 0.05 |
| ARAPAHOE | 0 | 98.8 | 2.5 | 100 | 0 | 6.2 | 0.79 | 2.2 | 0.07 |
| BENNETT | 0 | 100.0 | 0.0 | 100 | 0 | 6.7 | 0.00 | 2.1 | 0.03 |
| BUCKSKIN | 0 | 100.0 | 0.0 | 100 | 0 | 6.7 | 0.00 | 2.2 | 0.05 |
| CENTURK78 | 0 | 98.8 | 2.5 | 100 | 0 | 6.6 | 0.17 | 2.2 | 0.05 |
| CHEYENNE | 0 | 97.5 | 5.0 | 100 | 0 | 7.3 | 1.17 | 2.1 | 0.04 |
| COLT | 0 | 100.0 | 0.0 | 100 | 0 | 6.3 | 0.83 | 2.2 | 0.04 |
| COUGAR | 0 | 98.8 | 2.5 | 100 | 0 | 7.4 | 1.73 | 2.1 | 0.05 |
| CULVER | 0 | 98.8 | 2.5 | 100 | 0 | 6.6 | 0.17 | 2.1 | 0.02 |
| GAGE | 0 | 98.8 | 2.5 | 100 | 0 | 8.2 | 1.79 | 2.0 | 0.03 |
| GOODSTREAK | 0 | 100.0 | 0.0 | 100 | 0 | 7.5 | 1.67 | 2.1 | 0.06 |
| HALLAM | 0 | 97.5 | 5.0 | 100 | 0 | 7.3 | 1.81 | 2.1 | 0.05 |
| HARRY | 0 | 98.8 | 2.5 | 100 | 0 | 6.2 | 0.79 | 2.2 | 0.04 |
| HOMESTEAD | 0 | 100.0 | 0.0 | 100 | 0 | 7.5 | 1.67 | 2.1 | 0.06 |
| INFINITY_CL | 0 | 98.8 | 2.5 | 100 | 0 | 6.6 | 0.17 | 2.1 | 0.06 |
| KHARKOF | 0 | 100.0 | 0.0 | 100 | 0 | 6.7 | 0.00 | 2.1 | 0.03 |
| MILLENNIUM | 0 | 100.0 | 0.0 | 100 | 0 | 7.5 | 1.67 | 2.1 | 0.06 |
| CAMELOT | 0 | 98.8 | 2.5 | 100 | 0 | 6.6 | 0.17 | 2.1 | 0.02 |
| OVERLAND | 0 | 98.8 | 2.5 | 100 | 0 | 6.6 | 0.17 | 2.1 | 0.03 |
| NE99495 | 0 | 100.0 | 0.0 | 100 | 0 | 8.3 | 1.92 | 2.0 | 0.05 |
| NIOBRARA | 0 | 98.8 | 2.5 | 100 | 0 | 6.6 | 0.17 | 2.1 | 0.04 |
| NUPLAINS | 0 | 98.8 | 2.5 | 100 | 0 | 6.6 | 0.17 | 2.2 | 0.02 |
| PRONGHORN | 0 | 98.8 | 2.5 | 100 | 0 | 6.6 | 0.17 | 2.2 | 0.03 |
| RAWHIDE | 0 | 98.8 | 2.5 | 100 | 0 | 6.6 | 0.17 | 2.1 | 0.02 |
| REDLAND | 0 | 98.8 | 2.5 | 100 | 0 | 6.6 | 0.17 | 2.1 | 0.03 |
| SCOUT66 | 0 | 100.0 | 0.0 | 100 | 0 | 6.7 | 0.00 | 2.1 | 0.04 |
| SIOUXLAND | 0 | 97.5 | 2.9 | 100 | 0 | 6.1 | 0.92 | 2.2 | 0.03 |
| TURKEY_NEBSEL | 0 | 97.5 | 2.9 | 100 | 0 | 6.5 | 0.19 | 2.2 | 0.00 |
| VISTA | 0 | 97.5 | 2.9 | 100 | 0 | 6.1 | 0.74 | 2.1 | 0.07 |
| WAHOO | 0 | 100.0 | 0.0 | 100 | 0 | 6.7 | 0.00 | 2.2 | 0.04 |
| WARRIOR | 0 | 100.0 | 0.0 | 100 | 0 | 8.3 | 1.92 | 2.1 | 0.09 |
| WESLEY | 0 | 98.8 | 2.5 | 100 | 0 | 6.6 | 0.17 | 2.1 | 0.03 |
| WICHITA | 0 | 100.0 | 0.0 | 100 | 0 | 6.7 | 0.00 | 2.2 | 0.04 |
| WINDSTAR | 0 | 98.8 | 2.5 | 100 | 0 | 6.2 | 0.79 | 2.2 | 0.05 |
| LANCER | 0 | 100.0 | 0.0 | 100 | 0 | 6.7 | 0.00 | 2.2 | 0.03 |
| ANTON | 0 | 97.5 | 5.0 | 100 | 0 | 7.3 | 1.81 | 2.1 | 0.06 |
| MACE | 0 | 98.8 | 2.5 | 100 | 0 | 5.8 | 1.04 | 2.2 | 0.03 |
| TAM107-R7 | 0 | 98.8 | 2.5 | 100 | 0 | 6.6 | 0.17 | 2.1 | 0.05 |
| ARLIN | 0 | 96.3 | 4.8 | 100 | 0 | 6.0 | 0.72 | 2.2 | 0.04 |
| ALICE | 0 | 100.0 | 0.0 | 100 | 0 | 6.3 | 0.83 | 2.2 | 0.07 |
| DARRELL | 0 | 98.8 | 2.5 | 100 | 0 | 6.6 | 0.17 | 2.2 | 0.03 |
| EXPEDITION | 0 | 97.5 | 2.9 | 100 | 0 | 6.5 | 0.19 | 2.1 | 0.00 |
| WENDY | 0 | 100.0 | 0.0 | 100 | 0 | 6.7 | 0.00 | 2.2 | 0.03 |
| SD00111-9 | 0 | 97.5 | 2.9 | 100 | 0 | 6.5 | 0.19 | 2.1 | 0.03 |
| SD01237 | 0 | 98.8 | 2.5 | 100 | 0 | 6.6 | 0.17 | 2.1 | 0.03 |
| SD01058 | 0 | 98.8 | 2.5 | 100 | 0 | 6.6 | 0.17 | 2.2 | 0.04 |
| SD05118 | 0 | 100.0 | 0.0 | 100 | 0 | 6.7 | 0.00 | 2.1 | 0.05 |
| SD05210 | 0 | 96.3 | 2.5 | 100 | 0 | 6.4 | 0.17 | 2.2 | 0.05 |
| SD05W018 | 0 | 97.5 | 2.9 | 100 | 0 | 6.5 | 0.19 | 2.1 | 0.02 |
| NEKOTA | 0 | 98.8 | 2.5 | 100 | 0 | 6.6 | 0.17 | 2.1 | 0.05 |
| TANDEM | 0 | 97.5 | 5.0 | 100 | 0 | 6.5 | 0.33 | 2.1 | 0.03 |
| CRIMSON | 0 | 100.0 | 0.0 | 100 | 0 | 6.7 | 0.00 | 2.2 | 0.03 |
| ROSE | 0 | 97.5 | 2.9 | 100 | 0 | 6.5 | 0.19 | 2.2 | 0.00 |
| DAWN | 0 | 97.5 | 5.0 | 100 | 0 | 6.5 | 0.33 | 2.1 | 0.06 |
| WINOKA | 0 | 97.5 | 2.9 | 100 | 0 | 6.5 | 0.19 | 2.3 | 0.02 |
| NELL | 0 | 98.8 | 2.5 | 100 | 0 | 6.6 | 0.17 | 2.2 | 0.02 |
| RITA | 0 | 98.8 | 2.5 | 100 | 0 | 6.6 | 0.17 | 2.2 | 0.02 |
| BRONZE | 0 | 98.8 | 2.5 | 100 | 0 | 6.6 | 0.17 | 2.3 | 0.02 |
| HUME | 0 | 97.5 | 5.0 | 100 | 0 | 6.5 | 0.33 | 2.2 | 0.03 |
| GENT | 0 | 96.3 | 4.8 | 100 | 0 | 6.4 | 0.32 | 2.1 | 0.05 |
| HARDING | 0 | 100.0 | 0.0 | 100 | 0 | 6.7 | 0.00 | 2.2 | 0.04 |
| HV9W03-1551WP | 0 | 100.0 | 0.0 | 100 | 0 | 6.7 | 0.00 | 2.2 | 0.05 |
| G1878 | 0 | 100.0 | 0.0 | 100 | 0 | 6.7 | 0.00 | 2.1 | 0.03 |
| HV9W03-1379R | 0 | 98.8 | 2.5 | 100 | 0 | 6.6 | 0.17 | 2.3 | 0.01 |
| HV9W03-1596R | 0 | 98.8 | 2.5 | 100 | 0 | 6.6 | 0.17 | 2.2 | 0.04 |
| HV9W05-1280R | 0 | 97.5 | 5.0 | 100 | 0 | 6.5 | 0.33 | 2.1 | 0.02 |
| HV9W06-504 | 0 | 98.8 | 2.5 | 100 | 0 | 6.6 | 0.17 | 2.2 | 0.02 |
| SPARTAN | 0 | 98.8 | 2.5 | 100 | 0 | 6.6 | 0.17 | 2.2 | 0.03 |
| HV906-865 | 0 | 100.0 | 0.0 | 100 | 0 | 6.7 | 0.00 | 2.2 | 0.03 |
| TARKIO | 0 | 98.8 | 2.5 | 100 | 0 | 6.6 | 0.17 | 2.2 | 0.05 |
| SMOKYHILL | 0 | 93.8 | 2.5 | 100 | 0 | 6.3 | 0.17 | 2.2 | 0.03 |
| SHOCKER | 0 | 98.8 | 2.5 | 100 | 0 | 6.6 | 0.17 | 2.2 | 0.02 |
| VONA | 0 | 100.0 | 0.0 | 100 | 0 | 6.7 | 0.00 | 2.2 | 0.04 |
| CO940610 | 0 | 100.0 | 0.0 | 100 | 0 | 6.7 | 0.00 | 2.2 | 0.03 |
| AVALANCHE | 0 | 98.8 | 2.5 | 100 | 0 | 7.4 | 1.42 | 2.1 | 0.05 |
| BOND_CL | 0 | 100.0 | 0.0 | 100 | 0 | 6.7 | 0.00 | 2.2 | 0.03 |
| PLATTE | 0 | 98.8 | 2.5 | 100 | 0 | 6.6 | 0.17 | 2.2 | 0.03 |
| LINDON | 0 | 98.8 | 2.5 | 100 | 0 | 6.6 | 0.17 | 2.2 | 0.03 |
| CO03W043 | 0 | 100.0 | 0.0 | 100 | 0 | 6.7 | 0.00 | 2.2 | 0.05 |
| SNOWMASS | 0 | 96.3 | 4.8 | 100 | 0 | 6.4 | 0.32 | 2.1 | 0.03 |
| THUNDER_CL | 0 | 97.5 | 5.0 | 100 | 0 | 6.5 | 0.33 | 2.1 | 0.05 |
| CO04025 | 0 | 97.5 | 2.9 | 100 | 0 | 6.1 | 0.74 | 2.3 | 0.04 |
| CO04393 | 0 | 97.5 | 5.0 | 100 | 0 | 6.5 | 0.33 | 2.1 | 0.03 |
| CO04499 | 0 | 100.0 | 0.0 | 100 | 0 | 6.7 | 0.00 | 2.2 | 0.03 |
| CO04W320 | 0 | 98.8 | 2.5 | 100 | 0 | 7.4 | 1.42 | 2.1 | 0.04 |
| LAMAR | 0 | 100.0 | 0.0 | 100 | 0 | 6.7 | 0.00 | 2.1 | 0.03 |
| CARSON | 0 | 98.8 | 2.5 | 100 | 0 | 7.4 | 1.42 | 2.1 | 0.05 |
| HAIL | 0 | 97.5 | 2.9 | 100 | 0 | 6.5 | 0.19 | 2.3 | 0.01 |
| SANDY | 0 | 100.0 | 0.0 | 100 | 0 | 6.7 | 0.00 | 2.1 | 0.03 |
| DUKE | 0 | 96.3 | 4.8 | 100 | 0 | 6.4 | 0.32 | 2.2 | 0.02 |
| HALT | 0 | 100.0 | 0.0 | 100 | 0 | 6.7 | 0.00 | 2.2 | 0.05 |
| HATCHER | 0 | 100.0 | 0.0 | 100 | 0 | 6.7 | 0.00 | 2.2 | 0.03 |
| PRAIRIE_RED | 0 | 97.5 | 5.0 | 100 | 0 | 6.5 | 0.33 | 2.2 | 0.02 |
| ABOVE | 0 | 100.0 | 0.0 | 100 | 0 | 6.7 | 0.00 | 2.2 | 0.03 |
| CO03064 | 0 | 97.5 | 2.9 | 100 | 0 | 6.5 | 0.19 | 2.2 | 0.03 |
| BILL_BROWN | 0 | 98.8 | 2.5 | 100 | 0 | 6.6 | 0.17 | 2.2 | 0.02 |
| RIPPER | 0 | 97.5 | 2.9 | 100 | 0 | 6.5 | 0.19 | 2.2 | 0.02 |
| PROWERS | 0 | 98.8 | 2.5 | 100 | 0 | 6.6 | 0.17 | 2.2 | 0.03 |
| AKRON | 0 | 100.0 | 0.0 | 100 | 0 | 6.7 | 0.00 | 2.2 | 0.03 |
| JULES | 0 | 100.0 | 0.0 | 100 | 0 | 6.7 | 0.00 | 2.1 | 0.04 |
| YUMA | 0 | 97.5 | 2.9 | 100 | 0 | 6.5 | 0.19 | 2.2 | 0.03 |
| TAMW-101 | 0 | 97.5 | 5.0 | 100 | 0 | 6.5 | 0.33 | 2.1 | 0.03 |
| TAM105 | 0 | 98.8 | 2.5 | 100 | 0 | 6.2 | 0.79 | 2.2 | 0.05 |
| TAM107 | 0 | 98.8 | 2.5 | 100 | 0 | 6.6 | 0.17 | 2.1 | 0.05 |
| TAM109 | 0 | 98.8 | 2.5 | 100 | 0 | 6.6 | 0.17 | 2.2 | 0.03 |
| TAM110 | 0 | 98.8 | 2.5 | 100 | 0 | 6.2 | 0.96 | 2.2 | 0.03 |
| TAM111 | 0 | 96.3 | 4.8 | 100 | 0 | 6.4 | 0.32 | 2.2 | 0.01 |
| TAM112 | 0 | 98.8 | 2.5 | 100 | 0 | 6.6 | 0.17 | 2.2 | 0.03 |
| TAM200 | 0 | 98.8 | 2.5 | 100 | 0 | 6.2 | 0.79 | 2.2 | 0.07 |
| TAM202 | 0 | 96.3 | 4.8 | 100 | 0 | 6.4 | 0.32 | 2.2 | 0.02 |
| TAM203 | 0 | 96.3 | 4.8 | 100 | 0 | 6.4 | 0.32 | 2.2 | 0.05 |
| TAM302 | 0 | 100.0 | 0.0 | 100 | 0 | 6.7 | 0.00 | 2.2 | 0.03 |
| TAM303 | 0 | 100.0 | 0.0 | 100 | 0 | 7.5 | 1.67 | 2.1 | 0.09 |
| TAM304 | 0 | 98.8 | 2.5 | 100 | 0 | 6.6 | 0.17 | 2.1 | 0.05 |
| TAM400 | 0 | 97.5 | 2.9 | 100 | 0 | 7.3 | 1.78 | 2.2 | 0.13 |
| LOCKETT | 0 | 100.0 | 0.0 | 100 | 0 | 7.5 | 1.67 | 2.1 | 0.06 |
| STURDY | 0 | 97.5 | 2.9 | 100 | 0 | 6.5 | 0.19 | 2.1 | 0.02 |
| STURDY_2K | 0 | 98.8 | 2.5 | 100 | 0 | 6.6 | 0.17 | 2.1 | 0.02 |
| MIT | 0 | 97.5 | 2.9 | 100 | 0 | 6.5 | 0.19 | 2.2 | 0.02 |
| CAPROCK | 0 | 97.5 | 5.0 | 100 | 0 | 6.5 | 0.33 | 2.1 | 0.05 |
| TX01A5936 | 0 | 100.0 | 0.0 | 100 | 0 | 6.7 | 0.00 | 2.2 | 0.03 |
| TAM401 | 0 | 100.0 | 0.0 | 100 | 0 | 6.3 | 0.83 | 2.1 | 0.07 |
| TX02A0252 | 0 | 97.5 | 2.9 | 100 | 0 | 6.5 | 0.19 | 2.3 | 0.04 |
| TX03A0148 | 0 | 100.0 | 0.0 | 100 | 0 | 6.7 | 0.00 | 2.1 | 0.03 |
| TX03A0563 | 0 | 98.8 | 2.5 | 100 | 0 | 7.4 | 1.42 | 2.1 | 0.05 |
| TX04A001246 | 0 | 95.0 | 4.1 | 100 | 0 | 6.3 | 0.27 | 2.1 | 0.03 |
| TX01V5134RC-3 | 0 | 100.0 | 0.0 | 100 | 0 | 6.7 | 0.00 | 2.1 | 0.03 |
| TX04M410164 | 0 | 98.8 | 2.5 | 100 | 0 | 6.6 | 0.17 | 2.2 | 0.05 |
| TX04M410211 | 0 | 98.8 | 2.5 | 100 | 0 | 7.4 | 1.42 | 2.1 | 0.05 |
| TX04V075080 | 0 | 97.5 | 2.9 | 100 | 0 | 6.5 | 0.19 | 2.1 | 0.03 |
| TX99A0153-1 | 0 | 100.0 | 0.0 | 100 | 0 | 6.7 | 0.00 | 2.1 | 0.03 |
| TX01M5009-28 | 0 | 98.8 | 2.5 | 100 | 0 | 6.2 | 0.79 | 2.2 | 0.07 |
| TX00V1131 | 0 | 96.3 | 2.5 | 100 | 0 | 8.0 | 1.98 | 2.0 | 0.05 |
| TX99U8618 | 0 | 100.0 | 0.0 | 100 | 0 | 7.5 | 1.67 | 2.1 | 0.04 |
| TX96D1073 | 0 | 98.8 | 2.5 | 100 | 0 | 7.4 | 1.42 | 2.1 | 0.05 |
| 2180 | 0 | 95.0 | 7.1 | 100 | 0 | 6.3 | 0.47 | 2.1 | 0.01 |
| HG-9 | 0 | 96.3 | 2.5 | 100 | 0 | 7.2 | 1.54 | 2.1 | 0.04 |
| TX86A5606 | 0 | 98.8 | 2.5 | 100 | 0 | 7.4 | 1.42 | 2.1 | 0.04 |
| TX86A8072 | 0 | 100.0 | 0.0 | 100 | 0 | 7.5 | 1.67 | 2.1 | 0.03 |
| CREST | 0 | 100.0 | 0.0 | 100 | 0 | 6.7 | 0.00 | 2.2 | 0.03 |
| ROSEBUD | 0 | 98.8 | 2.5 | 100 | 0 | 6.6 | 0.17 | 2.2 | 0.05 |
| JUDITH | 0 | 98.8 | 2.5 | 100 | 0 | 6.6 | 0.17 | 2.1 | 0.05 |
| MT85200 | 0 | 100.0 | 0.0 | 100 | 0 | 7.5 | 1.67 | 2.0 | 0.03 |
| NUSKY | 0 | 97.5 | 2.9 | 100 | 0 | 5.7 | 0.95 | 2.2 | 0.07 |
| MT9513 | 0 | 98.8 | 2.5 | 100 | 0 | 7.4 | 1.42 | 2.1 | 0.05 |
| MT9904 | 0 | 97.5 | 5.0 | 100 | 0 | 7.3 | 1.81 | 2.1 | 0.05 |
| NORRIS | 0 | 98.8 | 2.5 | 100 | 0 | 6.6 | 0.17 | 2.1 | 0.00 |
| YELLOWSTONE | 0 | 100.0 | 0.0 | 100 | 0 | 6.7 | 0.00 | 2.1 | 0.04 |
| MT0495 | 0 | 98.8 | 2.5 | 100 | 0 | 6.6 | 0.17 | 2.1 | 0.00 |
| MTS0531 | 0 | 98.8 | 2.5 | 100 | 0 | 8.2 | 1.79 | 2.1 | 0.05 |
| DECADE | 0 | 97.5 | 2.9 | 100 | 0 | 6.5 | 0.19 | 2.1 | 0.05 |
| MT06103 | 0 | 96.3 | 4.8 | 100 | 0 | 6.4 | 0.32 | 2.2 | 0.02 |
| JUDEE | 0 | 98.8 | 2.5 | 100 | 0 | 6.6 | 0.17 | 2.2 | 0.03 |
| LAKIN | 0 | 98.8 | 2.5 | 100 | 0 | 6.6 | 0.17 | 2.1 | 0.03 |
| STANTON | 0 | 98.8 | 2.5 | 100 | 0 | 6.6 | 0.17 | 2.1 | 0.03 |
| TREGO | 0 | 98.8 | 2.5 | 100 | 0 | 6.6 | 0.17 | 2.1 | 0.03 |
| KARL_92 | 0 | 98.8 | 2.5 | 100 | 0 | 6.6 | 0.17 | 2.1 | 0.02 |
| DODGE | 0 | 96.3 | 4.8 | 100 | 0 | 6.4 | 0.32 | 2.2 | 0.02 |
| NORKAN | 0 | 98.8 | 2.5 | 100 | 0 | 6.6 | 0.17 | 2.2 | 0.03 |
| CHENEY | 0 | 98.8 | 2.5 | 100 | 0 | 6.6 | 0.17 | 2.1 | 0.00 |
| NEWTON | 0 | 98.8 | 2.5 | 100 | 0 | 6.6 | 0.17 | 2.2 | 0.04 |
| LARNED | 0 | 98.8 | 2.5 | 100 | 0 | 6.6 | 0.17 | 2.1 | 0.03 |
| PARKER76 | 0 | 98.8 | 2.5 | 100 | 0 | 6.6 | 0.17 | 2.2 | 0.04 |
| KIRWIN | 0 | 98.8 | 2.5 | 100 | 0 | 6.6 | 0.17 | 2.1 | 0.02 |
| SAGE | 0 | 98.8 | 2.5 | 100 | 0 | 6.6 | 0.17 | 2.2 | 0.04 |
| TRISON | 0 | 96.3 | 4.8 | 100 | 0 | 6.4 | 0.32 | 2.1 | 0.04 |
| EAGLE | 0 | 97.5 | 2.9 | 100 | 0 | 6.5 | 0.19 | 2.2 | 0.04 |
| SHAWNEE | 0 | 98.8 | 2.5 | 100 | 0 | 6.6 | 0.17 | 2.1 | 0.03 |
| PARKER | 0 | 98.8 | 2.5 | 100 | 0 | 6.6 | 0.17 | 2.1 | 0.00 |
| KAW61 | 0 | 96.3 | 7.5 | 100 | 0 | 6.4 | 0.50 | 2.1 | 0.02 |
| TASCOSA | 0 | 97.5 | 2.9 | 100 | 0 | 8.1 | 1.64 | 2.0 | 0.03 |
| BISON | 0 | 97.5 | 2.9 | 100 | 0 | 6.5 | 0.19 | 2.2 | 0.01 |
| KIOWA | 0 | 98.8 | 2.5 | 100 | 0 | 6.6 | 0.17 | 2.1 | 0.03 |
| WICHITA | 0 | 98.8 | 2.5 | 100 | 0 | 6.6 | 0.17 | 2.1 | 0.02 |
| COMANCHE | 0 | 97.5 | 2.9 | 100 | 0 | 7.3 | 1.48 | 2.0 | 0.03 |
| BAKERS_WHITE | 0 | 95.0 | 7.1 | 100 | 0 | 6.3 | 0.47 | 2.1 | 0.03 |
| BURCHETT | 0 | 98.8 | 2.5 | 100 | 0 | 6.6 | 0.17 | 2.2 | 0.03 |
| CUTTER | 0 | 98.8 | 2.5 | 100 | 0 | 6.6 | 0.17 | 2.1 | 0.02 |
| DUMAS | 0 | 96.3 | 4.8 | 100 | 0 | 6.4 | 0.32 | 2.2 | 0.02 |
| HONDO | 0 | 98.8 | 2.5 | 100 | 0 | 6.6 | 0.17 | 2.2 | 0.02 |
| JAGALENE | 0 | 98.8 | 2.5 | 100 | 0 | 6.6 | 0.17 | 2.2 | 0.02 |
| LONGHORN | 0 | 98.8 | 2.5 | 100 | 0 | 6.6 | 0.17 | 2.1 | 0.03 |
| NEOSHO | 0 | 98.8 | 2.5 | 100 | 0 | 6.6 | 0.17 | 2.1 | 0.02 |
| OGALLALA | 0 | 98.8 | 2.5 | 100 | 0 | 6.6 | 0.17 | 2.1 | 0.05 |
| POSTROCK | 0 | 96.3 | 4.8 | 100 | 0 | 6.4 | 0.32 | 2.1 | 0.03 |
| THUNDERBOLT | 0 | 97.5 | 2.9 | 100 | 0 | 6.5 | 0.19 | 2.2 | 0.05 |
| W04-417 | 0 | 97.5 | 2.9 | 100 | 0 | 6.5 | 0.19 | 2.1 | 0.02 |
| NUFRONTIER | 0 | 98.8 | 2.5 | 100 | 0 | 6.6 | 0.17 | 2.1 | 0.03 |
| NUHORIZON | 0 | 98.8 | 2.5 | 100 | 0 | 6.6 | 0.17 | 2.1 | 0.03 |
| ONAGA | 0 | 98.8 | 2.5 | 100 | 0 | 7.4 | 1.42 | 2.1 | 0.04 |
| RONL | 0 | 98.8 | 2.5 | 100 | 0 | 6.6 | 0.17 | 2.2 | 0.00 |
| 2145 | 0 | 98.8 | 2.5 | 100 | 0 | 6.6 | 0.17 | 2.1 | 0.03 |
| HEYNE | 0 | 96.3 | 4.8 | 100 | 0 | 6.4 | 0.32 | 2.2 | 0.04 |
| KS00F5-20-3 | 0 | 96.3 | 4.8 | 100 | 0 | 6.4 | 0.32 | 2.1 | 0.05 |
| OVERLEY | 0 | 98.8 | 2.5 | 100 | 0 | 6.6 | 0.17 | 2.2 | 0.01 |
| FULLER | 0 | 97.5 | 2.9 | 100 | 0 | 6.5 | 0.19 | 2.1 | 0.04 |
| COSSACK | 0 | 96.3 | 4.8 | 100 | 0 | 6.4 | 0.32 | 2.1 | 0.03 |
| ENHANCER | 0 | 98.8 | 2.5 | 100 | 0 | 6.6 | 0.17 | 2.1 | 0.03 |
| SANTA_FE | 0 | 98.8 | 2.5 | 100 | 0 | 6.6 | 0.17 | 2.2 | 0.03 |
| VENANGO | 0 | 98.8 | 2.5 | 100 | 0 | 6.6 | 0.17 | 2.1 | 0.03 |
| WB411W | 0 | 100.0 | 0.0 | 100 | 0 | 6.7 | 0.00 | 2.1 | 0.05 |
| KEOTA | 0 | 97.5 | 2.9 | 100 | 0 | 6.5 | 0.19 | 2.2 | 0.05 |
| TX05A001822 | 0 | 96.3 | 4.8 | 100 | 0 | 6.4 | 0.32 | 2.2 | 0.05 |
| TX06A001263 | 0 | 98.8 | 2.5 | 100 | 0 | 6.6 | 0.17 | 2.2 | 0.01 |
| TX06A001132 | 0 | 98.8 | 2.5 | 100 | 0 | 6.6 | 0.17 | 2.2 | 0.03 |
| TX06A001281 | 0 | 97.5 | 5.0 | 100 | 0 | 6.5 | 0.33 | 2.2 | 0.04 |
| TX06A001386 | 0 | 98.8 | 2.5 | 100 | 0 | 6.6 | 0.17 | 2.2 | 0.03 |
| TX05V7259 | 0 | 98.8 | 2.5 | 100 | 0 | 6.6 | 0.17 | 2.2 | 0.05 |
| TX05V7269 | 0 | 98.8 | 2.5 | 100 | 0 | 6.6 | 0.17 | 2.2 | 0.02 |
| TX05A001188 | 0 | 98.8 | 2.5 | 100 | 0 | 6.6 | 0.17 | 2.2 | 0.05 |
| TX07A001279 | 0 | 97.5 | 5.0 | 100 | 0 | 6.5 | 0.33 | 2.2 | 0.04 |
| TX07A001318 | 0 | 98.8 | 2.5 | 100 | 0 | 6.6 | 0.17 | 2.1 | 0.05 |
| TX07A001420 | 0 | 100.0 | 0.0 | 100 | 0 | 6.7 | 0.00 | 2.1 | 0.04 |
| TX06V7266 | 0 | 98.8 | 2.5 | 100 | 0 | 6.6 | 0.17 | 2.2 | 0.00 |
| OK1067071 | 0 | 96.3 | 4.8 | 100 | 0 | 6.4 | 0.32 | 2.1 | 0.04 |
| OK1067274 | 0 | 100.0 | 0.0 | 100 | 0 | 6.7 | 0.00 | 2.2 | 0.04 |
| OK1068002 | 0 | 98.8 | 2.5 | 100 | 0 | 6.6 | 0.17 | 2.1 | 0.03 |
| OK1068009 | 0 | 100.0 | 0.0 | 100 | 0 | 6.7 | 0.00 | 2.2 | 0.03 |
| OK1068026 | 0 | 98.8 | 2.5 | 100 | 0 | 6.6 | 0.17 | 2.2 | 0.04 |
| OK1068112 | 0 | 98.8 | 2.5 | 100 | 0 | 6.6 | 0.17 | 2.2 | 0.04 |
| OK1070275 | 0 | 100.0 | 0.0 | 100 | 0 | 6.7 | 0.00 | 2.2 | 0.00 |
| OK1070267 | 0 | 98.8 | 2.5 | 100 | 0 | 6.6 | 0.17 | 2.1 | 0.03 |
| OK09634 | 0 | 97.5 | 2.9 | 100 | 0 | 6.5 | 0.19 | 2.2 | 0.03 |
| OK10119 | 0 | 97.5 | 2.9 | 100 | 0 | 6.5 | 0.19 | 2.2 | 0.04 |
| GALLAGHER | 0 | 100.0 | 0.0 | 100 | 0 | 6.7 | 0.00 | 2.1 | 0.03 |
| OK07231 | 0 | 98.8 | 2.5 | 100 | 0 | 6.6 | 0.17 | 2.1 | 0.04 |
| OK07S117 | 0 | 100.0 | 0.0 | 100 | 0 | 6.7 | 0.00 | 2.2 | 0.03 |
| OK08328 | 0 | 98.8 | 2.5 | 100 | 0 | 6.6 | 0.17 | 2.1 | 0.00 |
| BIG_SKY | 0 | 100.0 | 0.0 | 100 | 0 | 6.7 | 0.00 | 2.2 | 0.03 |
| DANBY | 0 | 98.8 | 2.5 | 100 | 0 | 6.6 | 0.17 | 2.2 | 0.05 |
| E2041 | 0 | 97.5 | 5.0 | 100 | 0 | 6.5 | 0.33 | 2.2 | 0.03 |
| DENALI | 0 | 96.3 | 4.8 | 100 | 0 | 6.4 | 0.32 | 2.1 | 0.02 |
| CO050337-2 | 0 | 96.3 | 7.5 | 100 | 0 | 6.4 | 0.50 | 2.1 | 0.05 |
| BYRD | 0 | 98.8 | 2.5 | 100 | 0 | 6.6 | 0.17 | 2.1 | 0.03 |
| CO07W245 | 0 | 100.0 | 0.0 | 100 | 0 | 6.7 | 0.00 | 2.2 | 0.03 |
| MCGILL | 0 | 96.3 | 4.8 | 100 | 0 | 6.4 | 0.32 | 2.2 | 0.05 |
| NE02558 | 0 | 97.5 | 5.0 | 100 | 0 | 6.5 | 0.33 | 2.1 | 0.02 |
| NW03666 | 0 | 98.8 | 2.5 | 100 | 0 | 6.6 | 0.17 | 2.2 | 0.00 |
| NE04490 | 0 | 98.8 | 2.5 | 100 | 0 | 6.6 | 0.17 | 2.2 | 0.03 |
| NE05430 | 0 | 100.0 | 0.0 | 100 | 0 | 6.3 | 0.83 | 2.2 | 0.08 |
| NE05496 | 0 | 98.8 | 2.5 | 100 | 0 | 6.6 | 0.17 | 2.2 | 0.03 |
| NE05548 | 0 | 98.8 | 2.5 | 100 | 0 | 6.6 | 0.17 | 2.1 | 0.05 |
| NE06545 | 0 | 98.8 | 2.5 | 100 | 0 | 6.6 | 0.17 | 2.1 | 0.05 |
| NE06607 | 0 | 98.8 | 2.5 | 100 | 0 | 6.6 | 0.17 | 2.1 | 0.03 |
| ROBIDOUX | 0 | 100.0 | 0.0 | 100 | 0 | 6.7 | 0.00 | 2.1 | 0.03 |
| NI06736 | 0 | 98.8 | 2.5 | 100 | 0 | 6.6 | 0.17 | 2.1 | 0.05 |
| NI06737 | 0 | 96.3 | 4.8 | 100 | 0 | 6.4 | 0.32 | 2.1 | 0.04 |
| NI07703 | 0 | 97.5 | 5.0 | 100 | 0 | 7.3 | 1.81 | 2.1 | 0.05 |
| NI08707 | 0 | 97.5 | 5.0 | 100 | 0 | 6.5 | 0.33 | 2.2 | 0.02 |
| NI08708 | 0 | 96.3 | 4.8 | 100 | 0 | 6.4 | 0.32 | 2.2 | 0.04 |
| EVEREST | 0 | 97.5 | 5.0 | 100 | 0 | 6.5 | 0.33 | 2.1 | 0.03 |
| TRIUMPH64 | 60 | 90.0 | 7.1 | 91.2 | 7.5 | 3.8 | 0.66 | 2.9 | 0.17 |
| CHISHOLM | 60 | 90.0 | 7.1 | 92.4 | 8.6 | 4.0 | 0.50 | 2.7 | 0.11 |
| CUSTER | 60 | 88.8 | 2.5 | 92.4 | 5.1 | 4.2 | 1.24 | 2.8 | 0.20 |
| 2174-05 | 60 | 87.5 | 6.5 | 88.6 | 5.0 | 3.9 | 0.46 | 2.7 | 0.18 |
| INTRADA | 60 | 91.3 | 2.5 | 92.4 | 2.8 | 3.9 | 0.43 | 2.9 | 0.13 |
| OK101 | 60 | 91.3 | 2.5 | 93.6 | 2.4 | 3.7 | 0.10 | 3.0 | 0.12 |
| OK102 | 60 | 92.5 | 5.0 | 93.6 | 2.8 | 3.7 | 0.20 | 3.0 | 0.11 |
| ENDURANCE | 60 | 93.8 | 2.5 | 97.6 | 6.7 | 4.5 | 0.45 | 2.5 | 0.15 |
| DELIVER | 60 | 93.8 | 2.5 | 96.4 | 6.5 | 3.8 | 0.10 | 3.0 | 0.09 |
| OK_BULLET | 60 | 92.5 | 2.9 | 92.5 | 2.9 | 3.9 | 0.55 | 3.0 | 0.14 |
| CENTERFIELD | 60 | 82.5 | 2.9 | 83.6 | 2.4 | 3.5 | 0.35 | 2.9 | 0.23 |
| GUYMON | 60 | 96.3 | 2.5 | 97.5 | 2.9 | 5.6 | 1.02 | 2.5 | 0.15 |
| DUSTER | 60 | 85.0 | 0.0 | 87.4 | 4.7 | 3.4 | 0.00 | 2.9 | 0.15 |
| OK_RISING | 60 | 88.8 | 2.5 | 88.8 | 2.5 | 3.8 | 0.49 | 2.8 | 0.12 |
| OK02405 | 60 | 92.5 | 2.9 | 93.7 | 2.5 | 3.7 | 0.12 | 2.9 | 0.08 |
| PETE | 60 | 93.8 | 2.5 | 94.9 | 0.1 | 4.0 | 0.52 | 3.0 | 0.21 |
| BILLINGS | 60 | 81.3 | 2.5 | 84.8 | 6.7 | 3.3 | 0.10 | 3.0 | 0.20 |
| OK04505 | 60 | 92.5 | 2.9 | 92.5 | 2.9 | 3.9 | 0.39 | 2.8 | 0.23 |
| OK04525 | 60 | 90.0 | 4.1 | 90.0 | 4.1 | 3.6 | 0.16 | 2.8 | 0.04 |
| OK04507 | 60 | 93.8 | 2.5 | 93.8 | 2.5 | 4.0 | 0.35 | 2.7 | 0.12 |
| OK05830 | 60 | 91.3 | 2.5 | 93.8 | 4.8 | 3.9 | 0.43 | 2.9 | 0.18 |
| OK04111 | 60 | 93.8 | 2.5 | 95.0 | 4.1 | 3.8 | 0.10 | 3.1 | 0.27 |
| OK04415 | 60 | 91.3 | 2.5 | 91.3 | 2.5 | 3.9 | 0.43 | 3.0 | 0.15 |
| OK05711W | 60 | 93.8 | 2.5 | 96.4 | 6.5 | 3.8 | 0.10 | 2.9 | 0.12 |
| OK05723W | 60 | 95.0 | 0.0 | 95.0 | 0.0 | 3.8 | 0.00 | 2.9 | 0.05 |
| OK05108 | 60 | 95.0 | 0.0 | 96.3 | 2.5 | 3.8 | 0.00 | 3.0 | 0.05 |
| OK05122 | 60 | 86.3 | 2.5 | 88.6 | 4.5 | 3.5 | 0.10 | 3.1 | 0.17 |
| OK05526 | 60 | 92.5 | 2.9 | 92.5 | 2.9 | 3.7 | 0.12 | 3.1 | 0.08 |
| OK05134 | 60 | 93.8 | 2.5 | 95.0 | 4.1 | 3.8 | 0.10 | 3.0 | 0.05 |
| OK05303 | 60 | 91.3 | 2.5 | 92.4 | 2.8 | 3.7 | 0.10 | 3.0 | 0.04 |
| OK05312 | 60 | 93.8 | 2.5 | 95.0 | 4.1 | 4.0 | 0.52 | 3.0 | 0.16 |
| OK05511 | 60 | 91.3 | 4.8 | 92.5 | 6.5 | 3.7 | 0.19 | 3.0 | 0.18 |
| OK05204 | 60 | 93.8 | 2.5 | 95.0 | 4.1 | 3.8 | 0.10 | 3.1 | 0.11 |
| GARRISON | 60 | 95.0 | 0.0 | 97.6 | 5.3 | 4.3 | 0.55 | 2.8 | 0.22 |
| OK06114 | 60 | 93.8 | 2.5 | 97.9 | 9.5 | 3.8 | 0.10 | 3.1 | 0.13 |
| OK06210 | 60 | 93.8 | 2.5 | 93.8 | 2.5 | 3.8 | 0.10 | 2.8 | 0.05 |
| OK06319 | 60 | 92.5 | 2.9 | 93.7 | 2.5 | 3.7 | 0.12 | 3.1 | 0.03 |
| OK06318 | 60 | 85.0 | 16.8 | 85.0 | 16.8 | 3.4 | 0.67 | 3.1 | 0.14 |
| OK06336 | 60 | 95.0 | 0.0 | 97.6 | 5.3 | 3.8 | 0.00 | 2.9 | 0.18 |
| AGATE | 60 | 93.8 | 2.5 | 94.9 | 0.1 | 4.0 | 0.52 | 2.9 | 0.10 |
| ALLIANCE | 60 | 91.3 | 7.5 | 92.5 | 8.7 | 3.7 | 0.30 | 2.9 | 0.04 |
| ANTELOPE | 60 | 95.0 | 0.0 | 96.3 | 2.5 | 3.8 | 0.00 | 3.1 | 0.05 |
| ARAPAHOE | 60 | 95.0 | 0.0 | 96.3 | 2.5 | 4.3 | 0.55 | 2.9 | 0.19 |
| BENNETT | 60 | 95.0 | 0.0 | 95.0 | 0.0 | 3.8 | 0.00 | 3.1 | 0.11 |
| BUCKSKIN | 60 | 92.5 | 2.9 | 92.5 | 2.9 | 3.7 | 0.12 | 2.8 | 0.05 |
| CENTURK78 | 60 | 95.0 | 0.0 | 96.3 | 2.5 | 4.0 | 0.48 | 2.9 | 0.18 |
| CHEYENNE | 60 | 95.0 | 0.0 | 97.6 | 5.3 | 4.0 | 0.48 | 2.9 | 0.25 |
| COLT | 60 | 92.5 | 2.9 | 92.5 | 2.9 | 3.7 | 0.12 | 2.9 | 0.12 |
| COUGAR | 60 | 93.8 | 4.8 | 94.9 | 4.1 | 4.0 | 0.54 | 2.8 | 0.15 |
| CULVER | 60 | 95.0 | 4.1 | 96.2 | 2.5 | 3.8 | 0.16 | 3.0 | 0.07 |
| GAGE | 60 | 96.3 | 2.5 | 97.5 | 2.9 | 4.8 | 0.13 | 2.6 | 0.02 |
| GOODSTREAK | 60 | 95.0 | 4.1 | 95.0 | 4.1 | 3.8 | 0.16 | 3.0 | 0.09 |
| HALLAM | 60 | 93.8 | 2.5 | 96.4 | 6.5 | 3.8 | 0.10 | 3.1 | 0.03 |
| HARRY | 60 | 95.0 | 0.0 | 96.3 | 2.5 | 3.8 | 0.00 | 3.1 | 0.12 |
| HOMESTEAD | 60 | 93.8 | 4.8 | 93.8 | 4.8 | 4.0 | 0.39 | 2.8 | 0.10 |
| INFINITY_CL | 60 | 93.8 | 2.5 | 94.9 | 0.1 | 3.8 | 0.10 | 2.9 | 0.12 |
| KHARKOF | 60 | 93.8 | 2.5 | 93.8 | 2.5 | 4.2 | 0.49 | 2.7 | 0.13 |
| MILLENNIUM | 60 | 92.5 | 2.9 | 92.5 | 2.9 | 3.7 | 0.12 | 3.0 | 0.04 |
| CAMELOT | 60 | 96.3 | 2.5 | 97.5 | 2.9 | 3.9 | 0.10 | 3.0 | 0.11 |
| OVERLAND | 60 | 95.0 | 0.0 | 96.3 | 2.5 | 3.8 | 0.00 | 3.0 | 0.09 |
| NE99495 | 60 | 96.3 | 2.5 | 96.3 | 2.5 | 3.9 | 0.10 | 2.9 | 0.09 |
| NIOBRARA | 60 | 92.5 | 2.9 | 93.8 | 4.8 | 3.9 | 0.55 | 2.8 | 0.21 |
| NUPLAINS | 60 | 93.8 | 4.8 | 95.0 | 5.8 | 4.0 | 0.54 | 2.7 | 0.04 |
| PRONGHORN | 60 | 93.8 | 2.5 | 94.9 | 0.1 | 4.0 | 0.35 | 2.9 | 0.17 |
| RAWHIDE | 60 | 92.5 | 2.9 | 93.8 | 4.8 | 3.7 | 0.12 | 2.9 | 0.13 |
| REDLAND | 60 | 95.0 | 4.1 | 96.3 | 6.4 | 3.8 | 0.16 | 3.0 | 0.15 |
| SCOUT66 | 60 | 92.5 | 2.9 | 92.5 | 2.9 | 3.7 | 0.12 | 3.0 | 0.05 |
| SIOUXLAND | 60 | 83.8 | 2.5 | 85.9 | 2.4 | 3.6 | 0.30 | 2.8 | 0.19 |
| TURKEY_NEBSEL | 60 | 95.0 | 0.0 | 97.5 | 2.9 | 3.8 | 0.00 | 3.1 | 0.04 |
| VISTA | 60 | 91.3 | 2.5 | 93.6 | 2.4 | 3.9 | 0.43 | 2.8 | 0.19 |
| WAHOO | 60 | 93.8 | 4.8 | 93.8 | 4.8 | 3.8 | 0.19 | 3.1 | 0.17 |
| WARRIOR | 60 | 95.0 | 4.1 | 95.0 | 4.1 | 4.0 | 0.50 | 2.8 | 0.20 |
| WESLEY | 60 | 93.8 | 2.5 | 95.0 | 4.1 | 4.0 | 0.52 | 2.9 | 0.09 |
| WICHITA | 60 | 93.8 | 6.3 | 93.8 | 6.3 | 3.8 | 0.25 | 2.9 | 0.06 |
| WINDSTAR | 60 | 93.8 | 6.3 | 94.9 | 4.3 | 3.8 | 0.25 | 3.1 | 0.17 |
| LANCER | 60 | 92.5 | 2.9 | 92.5 | 2.9 | 3.7 | 0.12 | 3.0 | 0.12 |
| ANTON | 60 | 91.3 | 2.5 | 93.8 | 4.8 | 3.7 | 0.10 | 2.9 | 0.21 |
| MACE | 60 | 93.8 | 4.8 | 95.1 | 7.2 | 3.8 | 0.19 | 2.9 | 0.09 |
| TAM107-R7 | 60 | 92.5 | 2.9 | 93.7 | 2.5 | 3.7 | 0.12 | 2.9 | 0.03 |
| ARLIN | 60 | 93.8 | 2.5 | 97.5 | 2.9 | 3.8 | 0.10 | 3.0 | 0.14 |
| ALICE | 60 | 96.3 | 2.5 | 96.3 | 2.5 | 3.9 | 0.10 | 2.9 | 0.18 |
| DARRELL | 60 | 95.0 | 0.0 | 96.3 | 2.5 | 4.0 | 0.48 | 2.9 | 0.23 |
| EXPEDITION | 60 | 92.5 | 2.9 | 94.9 | 4.1 | 3.7 | 0.12 | 3.0 | 0.08 |
| WENDY | 60 | 96.3 | 2.5 | 96.3 | 2.5 | 3.9 | 0.10 | 3.0 | 0.03 |
| SD00111-9 | 60 | 93.8 | 2.5 | 96.3 | 4.8 | 3.8 | 0.10 | 3.0 | 0.07 |
| SD01237 | 60 | 93.8 | 2.5 | 94.9 | 0.1 | 4.0 | 0.52 | 2.9 | 0.20 |
| SD01058 | 60 | 96.3 | 2.5 | 97.5 | 2.9 | 4.1 | 0.45 | 2.9 | 0.23 |
| SD05118 | 60 | 95.0 | 0.0 | 95.0 | 0.0 | 4.7 | 1.20 | 2.7 | 0.22 |
| SD05210 | 60 | 91.3 | 7.5 | 94.8 | 7.4 | 4.3 | 1.40 | 2.8 | 0.20 |
| SD05W018 | 60 | 93.8 | 2.5 | 96.3 | 4.8 | 3.8 | 0.10 | 3.0 | 0.11 |
| NEKOTA | 60 | 95.0 | 0.0 | 96.3 | 2.5 | 4.0 | 0.48 | 2.9 | 0.17 |
| TANDEM | 60 | 95.0 | 0.0 | 97.6 | 5.3 | 3.8 | 0.00 | 3.0 | 0.06 |
| CRIMSON | 60 | 96.3 | 2.5 | 96.3 | 2.5 | 3.9 | 0.10 | 3.0 | 0.11 |
| ROSE | 60 | 91.3 | 4.8 | 93.7 | 6.3 | 4.1 | 0.51 | 2.8 | 0.18 |
| DAWN | 60 | 96.3 | 2.5 | 98.9 | 5.0 | 3.9 | 0.10 | 3.0 | 0.07 |
| WINOKA | 60 | 93.8 | 2.5 | 96.3 | 4.8 | 3.8 | 0.10 | 2.9 | 0.09 |
| NELL | 60 | 93.8 | 2.5 | 95.0 | 4.1 | 4.2 | 0.61 | 2.8 | 0.17 |
| RITA | 60 | 93.8 | 4.8 | 95.1 | 7.2 | 3.8 | 0.19 | 2.9 | 0.18 |
| BRONZE | 60 | 95.0 | 4.1 | 96.3 | 4.8 | 3.8 | 0.16 | 3.0 | 0.12 |
| HUME | 60 | 93.8 | 6.3 | 96.1 | 2.6 | 3.8 | 0.25 | 3.0 | 0.12 |
| GENT | 60 | 93.8 | 2.5 | 97.6 | 5.3 | 3.8 | 0.10 | 2.9 | 0.15 |
| HARDING | 60 | 93.8 | 4.8 | 93.8 | 4.8 | 3.8 | 0.19 | 3.0 | 0.09 |
| HV9W03-1551WP | 60 | 95.0 | 0.0 | 95.0 | 0.0 | 3.8 | 0.00 | 2.9 | 0.04 |
| G1878 | 60 | 93.8 | 2.5 | 93.8 | 2.5 | 3.8 | 0.10 | 2.9 | 0.13 |
| HV9W03-1379R | 60 | 95.0 | 4.1 | 96.2 | 2.5 | 3.8 | 0.16 | 3.0 | 0.07 |
| HV9W03-1596R | 60 | 93.8 | 2.5 | 95.0 | 4.1 | 3.8 | 0.10 | 2.9 | 0.06 |
| HV9W05-1280R | 60 | 93.8 | 2.5 | 96.3 | 2.5 | 3.8 | 0.10 | 3.0 | 0.12 |
| HV9W06-504 | 60 | 95.0 | 0.0 | 96.3 | 2.5 | 3.8 | 0.00 | 3.1 | 0.11 |
| SPARTAN | 60 | 93.8 | 2.5 | 94.9 | 0.1 | 3.8 | 0.10 | 3.0 | 0.08 |
| HV906-865 | 60 | 95.0 | 0.0 | 95.0 | 0.0 | 3.8 | 0.00 | 3.0 | 0.13 |
| TARKIO | 60 | 93.8 | 2.5 | 95.0 | 4.1 | 3.8 | 0.10 | 2.9 | 0.03 |
| SMOKYHILL | 60 | 85.0 | 0.0 | 90.7 | 2.5 | 3.6 | 0.43 | 2.9 | 0.13 |
| SHOCKER | 60 | 93.8 | 2.5 | 94.9 | 0.1 | 4.0 | 0.52 | 2.8 | 0.14 |
| VONA | 60 | 93.8 | 4.8 | 93.8 | 4.8 | 3.8 | 0.19 | 3.0 | 0.14 |
| CO940610 | 60 | 93.8 | 2.5 | 93.8 | 2.5 | 3.8 | 0.10 | 3.0 | 0.10 |
| AVALANCHE | 60 | 97.5 | 2.9 | 98.8 | 2.5 | 4.8 | 1.10 | 2.8 | 0.29 |
| BOND_CL | 60 | 93.8 | 2.5 | 93.8 | 2.5 | 3.8 | 0.10 | 2.9 | 0.07 |
| PLATTE | 60 | 93.8 | 2.5 | 95.0 | 4.1 | 3.8 | 0.10 | 3.0 | 0.07 |
| LINDON | 60 | 95.0 | 4.1 | 96.3 | 4.8 | 3.8 | 0.16 | 3.0 | 0.11 |
| CO03W043 | 60 | 96.3 | 2.5 | 96.3 | 2.5 | 3.9 | 0.10 | 3.0 | 0.08 |
| SNOWMASS | 60 | 92.5 | 2.9 | 96.2 | 2.5 | 3.9 | 0.39 | 2.8 | 0.20 |
| THUNDER_CL | 60 | 93.8 | 2.5 | 96.4 | 6.5 | 3.8 | 0.10 | 2.9 | 0.09 |
| CO04025 | 60 | 95.0 | 4.1 | 97.4 | 3.0 | 3.8 | 0.16 | 3.0 | 0.10 |
| CO04393 | 60 | 92.5 | 2.9 | 95.1 | 7.3 | 3.9 | 0.55 | 2.8 | 0.15 |
| CO04499 | 60 | 95.0 | 0.0 | 95.0 | 0.0 | 3.8 | 0.00 | 3.1 | 0.18 |
| CO04W320 | 60 | 77.5 | 5.0 | 78.6 | 6.0 | 4.5 | 0.64 | 2.6 | 0.01 |
| LAMAR | 60 | 93.8 | 4.8 | 93.8 | 4.8 | 4.2 | 0.64 | 2.7 | 0.11 |
| CARSON | 60 | 86.3 | 2.5 | 87.4 | 2.7 | 3.9 | 0.44 | 3.0 | 0.33 |
| HAIL | 60 | 88.8 | 2.5 | 91.1 | 4.7 | 3.6 | 0.10 | 2.9 | 0.11 |
| SANDY | 60 | 93.8 | 2.5 | 93.8 | 2.5 | 3.8 | 0.10 | 2.9 | 0.09 |
| DUKE | 60 | 95.0 | 0.0 | 98.9 | 5.0 | 3.8 | 0.00 | 3.0 | 0.06 |
| HALT | 60 | 93.8 | 2.5 | 93.8 | 2.5 | 3.8 | 0.10 | 3.0 | 0.07 |
| HATCHER | 60 | 82.5 | 2.9 | 82.5 | 2.9 | 3.3 | 0.12 | 3.3 | 0.25 |
| PRAIRIE_RED | 60 | 93.8 | 2.5 | 96.4 | 6.5 | 3.8 | 0.10 | 3.0 | 0.11 |
| ABOVE | 60 | 96.3 | 2.5 | 96.3 | 2.5 | 3.9 | 0.10 | 3.0 | 0.03 |
| CO03064 | 60 | 93.8 | 2.5 | 96.3 | 4.8 | 3.8 | 0.10 | 3.0 | 0.08 |
| BILL_BROWN | 60 | 88.8 | 2.5 | 89.9 | 4.0 | 3.6 | 0.10 | 3.2 | 0.27 |
| RIPPER | 60 | 91.3 | 2.5 | 93.6 | 2.4 | 3.7 | 0.10 | 3.0 | 0.12 |
| PROWERS | 60 | 93.8 | 2.5 | 95.0 | 4.1 | 3.8 | 0.10 | 3.0 | 0.15 |
| AKRON | 60 | 92.5 | 2.9 | 92.5 | 2.9 | 3.7 | 0.12 | 3.0 | 0.13 |
| JULES | 60 | 93.8 | 4.8 | 93.8 | 4.8 | 3.8 | 0.19 | 2.9 | 0.15 |
| YUMA | 60 | 93.8 | 2.5 | 96.2 | 2.5 | 3.8 | 0.10 | 3.1 | 0.15 |
| TAMW-101 | 60 | 93.8 | 2.5 | 96.4 | 6.5 | 3.8 | 0.10 | 3.1 | 0.07 |
| TAM105 | 60 | 95.0 | 0.0 | 96.3 | 2.5 | 3.8 | 0.00 | 3.0 | 0.07 |
| TAM107 | 60 | 91.3 | 2.5 | 92.5 | 5.0 | 3.7 | 0.10 | 2.9 | 0.13 |
| TAM109 | 60 | 96.3 | 2.5 | 97.6 | 5.1 | 3.9 | 0.10 | 3.0 | 0.07 |
| TAM110 | 60 | 93.8 | 2.5 | 95.0 | 4.1 | 3.8 | 0.10 | 2.9 | 0.15 |
| TAM111 | 60 | 95.0 | 0.0 | 98.9 | 5.0 | 3.8 | 0.00 | 3.0 | 0.13 |
| TAM112 | 60 | 93.8 | 2.5 | 95.0 | 4.1 | 3.8 | 0.10 | 3.0 | 0.13 |
| TAM200 | 60 | 95.0 | 4.1 | 96.2 | 2.5 | 4.0 | 0.50 | 2.8 | 0.15 |
| TAM202 | 60 | 78.8 | 4.8 | 81.8 | 2.8 | 3.3 | 0.32 | 2.9 | 0.13 |
| TAM203 | 60 | 95.0 | 0.0 | 98.9 | 5.0 | 4.0 | 0.48 | 3.0 | 0.26 |
| TAM302 | 60 | 95.0 | 0.0 | 95.0 | 0.0 | 3.8 | 0.00 | 2.9 | 0.05 |
| TAM303 | 60 | 87.5 | 2.9 | 87.5 | 2.9 | 3.5 | 0.12 | 3.0 | 0.07 |
| TAM304 | 60 | 93.8 | 2.5 | 95.0 | 4.1 | 4.0 | 0.52 | 2.9 | 0.16 |
| TAM400 | 60 | 80.0 | 0.0 | 82.1 | 2.4 | 3.2 | 0.00 | 3.2 | 0.06 |
| LOCKETT | 60 | 93.8 | 2.5 | 93.8 | 2.5 | 3.8 | 0.10 | 3.2 | 0.23 |
| STURDY | 60 | 85.0 | 4.1 | 87.2 | 4.8 | 3.8 | 0.38 | 2.9 | 0.25 |
| STURDY_2K | 60 | 93.8 | 6.3 | 95.0 | 7.1 | 3.8 | 0.25 | 3.2 | 0.23 |
| MIT | 60 | 87.5 | 6.5 | 89.7 | 4.4 | 4.6 | 1.05 | 2.8 | 0.32 |
| CAPROCK | 60 | 92.5 | 2.9 | 95.0 | 4.1 | 3.7 | 0.12 | 2.9 | 0.12 |
| TX01A5936 | 60 | 95.0 | 4.1 | 95.0 | 4.1 | 3.8 | 0.16 | 3.0 | 0.16 |
| TAM401 | 60 | 95.0 | 4.1 | 95.0 | 4.1 | 4.0 | 0.50 | 2.9 | 0.26 |
| TX02A0252 | 60 | 95.0 | 0.0 | 97.5 | 2.9 | 3.8 | 0.00 | 2.9 | 0.10 |
| TX03A0148 | 60 | 88.8 | 2.5 | 88.8 | 2.5 | 3.6 | 0.10 | 3.1 | 0.05 |
| TX03A0563 | 60 | 87.5 | 2.9 | 88.6 | 2.4 | 3.5 | 0.12 | 3.0 | 0.14 |
| TX04A001246 | 60 | 91.3 | 4.8 | 96.1 | 5.0 | 4.1 | 0.35 | 2.8 | 0.27 |
| TX01V5134RC-3 | 60 | 97.5 | 2.9 | 97.5 | 2.9 | 3.9 | 0.12 | 2.9 | 0.09 |
| TX04M410164 | 60 | 90.0 | 4.1 | 91.2 | 4.7 | 3.6 | 0.16 | 3.0 | 0.23 |
| TX04M410211 | 60 | 95.0 | 0.0 | 96.3 | 2.5 | 4.0 | 0.48 | 2.8 | 0.14 |
| TX04V075080 | 60 | 85.0 | 0.0 | 87.2 | 2.6 | 3.4 | 0.00 | 3.1 | 0.07 |
| TX99A0153-1 | 60 | 87.5 | 6.5 | 87.5 | 6.5 | 4.2 | 0.68 | 2.6 | 0.13 |
| TX01M5009-28 | 60 | 87.5 | 5.0 | 88.6 | 4.7 | 3.5 | 0.20 | 2.9 | 0.05 |
| TX00V1131 | 60 | 92.5 | 2.9 | 96.1 | 2.6 | 3.7 | 0.12 | 3.0 | 0.07 |
| TX99U8618 | 60 | 93.8 | 4.8 | 93.8 | 4.8 | 3.8 | 0.19 | 2.9 | 0.13 |
| TX96D1073 | 60 | 91.3 | 4.8 | 92.5 | 6.5 | 4.3 | 0.41 | 2.7 | 0.27 |
| 2180 | 60 | 95.0 | 4.1 | 100.5 | 9.8 | 3.8 | 0.16 | 2.9 | 0.15 |
| HG-9 | 60 | 88.8 | 4.8 | 92.3 | 6.5 | 3.6 | 0.19 | 3.0 | 0.12 |
| TX86A5606 | 60 | 95.0 | 4.1 | 96.3 | 4.8 | 4.0 | 0.33 | 2.8 | 0.12 |
| TX86A8072 | 60 | 90.0 | 4.1 | 90.0 | 4.1 | 4.5 | 0.20 | 2.6 | 0.04 |
| CREST | 60 | 96.3 | 7.5 | 96.3 | 7.5 | 4.1 | 0.13 | 2.9 | 0.23 |
| ROSEBUD | 60 | 95.0 | 0.0 | 96.3 | 2.5 | 3.8 | 0.00 | 2.9 | 0.04 |
| JUDITH | 60 | 91.3 | 4.8 | 92.5 | 6.5 | 4.1 | 0.51 | 2.8 | 0.22 |
| MT85200 | 60 | 92.5 | 5.0 | 92.5 | 5.0 | 4.4 | 0.46 | 2.6 | 0.11 |
| NUSKY | 60 | 91.3 | 6.3 | 93.7 | 7.7 | 3.7 | 0.25 | 2.9 | 0.10 |
| MT9513 | 60 | 91.3 | 4.8 | 92.4 | 3.0 | 4.1 | 0.73 | 2.7 | 0.12 |
| MT9904 | 60 | 93.8 | 2.5 | 96.4 | 6.5 | 4.0 | 0.35 | 2.8 | 0.16 |
| NORRIS | 60 | 91.3 | 4.8 | 92.4 | 5.0 | 3.7 | 0.19 | 2.9 | 0.12 |
| YELLOWSTONE | 60 | 95.0 | 4.1 | 95.0 | 4.1 | 3.8 | 0.16 | 2.9 | 0.14 |
| MT0495 | 60 | 92.5 | 2.9 | 93.7 | 2.5 | 3.7 | 0.12 | 3.0 | 0.09 |
| MTS0531 | 60 | 98.8 | 2.5 | 100.1 | 4.2 | 4.9 | 1.26 | 2.7 | 0.19 |
| DECADE | 60 | 95.0 | 4.1 | 97.6 | 6.6 | 3.8 | 0.16 | 3.0 | 0.05 |
| MT06103 | 60 | 88.8 | 2.5 | 92.4 | 6.4 | 4.0 | 0.46 | 2.7 | 0.14 |
| JUDEE | 60 | 88.8 | 2.5 | 89.9 | 4.0 | 3.6 | 0.10 | 3.0 | 0.14 |
| LAKIN | 60 | 91.3 | 2.5 | 92.4 | 2.8 | 3.7 | 0.10 | 3.1 | 0.05 |
| STANTON | 60 | 92.5 | 2.9 | 93.8 | 4.8 | 3.7 | 0.12 | 3.0 | 0.14 |
| TREGO | 60 | 90.0 | 4.1 | 91.2 | 4.7 | 4.3 | 0.33 | 2.6 | 0.15 |
| KARL_92 | 60 | 92.5 | 5.0 | 93.8 | 6.3 | 3.7 | 0.20 | 2.9 | 0.05 |
| DODGE | 60 | 93.8 | 4.8 | 97.6 | 6.6 | 3.8 | 0.19 | 2.9 | 0.13 |
| NORKAN | 60 | 93.8 | 2.5 | 95.0 | 4.1 | 3.8 | 0.10 | 2.9 | 0.07 |
| CHENEY | 60 | 91.3 | 7.5 | 92.4 | 6.5 | 3.7 | 0.30 | 2.9 | 0.04 |
| NEWTON | 60 | 91.3 | 4.8 | 92.5 | 6.5 | 3.9 | 0.28 | 2.8 | 0.13 |
| LARNED | 60 | 91.3 | 7.5 | 92.5 | 8.7 | 3.9 | 0.36 | 2.8 | 0.08 |
| PARKER76 | 60 | 92.5 | 2.9 | 93.8 | 4.8 | 3.7 | 0.12 | 3.0 | 0.11 |
| KIRWIN | 60 | 95.0 | 4.1 | 96.3 | 4.8 | 3.8 | 0.16 | 3.0 | 0.04 |
| SAGE | 60 | 95.0 | 4.1 | 96.3 | 4.8 | 3.8 | 0.16 | 2.8 | 0.08 |
| TRISON | 60 | 93.8 | 2.5 | 97.6 | 6.7 | 3.8 | 0.10 | 2.9 | 0.11 |
| EAGLE | 60 | 90.0 | 5.8 | 92.4 | 6.5 | 3.6 | 0.23 | 2.8 | 0.08 |
| SHAWNEE | 60 | 95.0 | 4.1 | 96.2 | 2.5 | 3.8 | 0.16 | 3.1 | 0.07 |
| PARKER | 60 | 93.8 | 6.3 | 95.0 | 7.1 | 4.0 | 0.21 | 2.8 | 0.14 |
| KAW61 | 60 | 93.8 | 2.5 | 97.9 | 9.5 | 4.2 | 0.61 | 2.6 | 0.17 |
| TASCOSA | 60 | 95.0 | 4.1 | 97.6 | 6.6 | 5.1 | 0.60 | 2.5 | 0.07 |
| BISON | 60 | 91.3 | 4.8 | 93.6 | 4.9 | 3.7 | 0.19 | 2.9 | 0.13 |
| KIOWA | 60 | 96.3 | 4.8 | 97.5 | 5.0 | 3.9 | 0.19 | 2.9 | 0.15 |
| WICHITA | 60 | 91.3 | 2.5 | 92.5 | 5.0 | 4.1 | 0.47 | 2.8 | 0.16 |
| COMANCHE | 60 | 91.3 | 4.8 | 93.7 | 6.3 | 3.7 | 0.19 | 2.8 | 0.08 |
| BAKERS_WHITE | 60 | 91.3 | 4.8 | 96.3 | 6.9 | 3.7 | 0.19 | 2.9 | 0.03 |
| BURCHETT | 60 | 93.8 | 2.5 | 94.9 | 0.1 | 3.8 | 0.10 | 3.0 | 0.09 |
| CUTTER | 60 | 93.8 | 6.3 | 95.0 | 7.1 | 4.0 | 0.21 | 2.8 | 0.21 |
| DUMAS | 60 | 92.5 | 2.9 | 96.2 | 2.5 | 3.9 | 0.55 | 2.8 | 0.21 |
| HONDO | 60 | 93.8 | 6.3 | 95.0 | 7.1 | 4.7 | 0.31 | 2.6 | 0.04 |
| JAGALENE | 60 | 92.5 | 2.9 | 93.7 | 2.5 | 3.7 | 0.12 | 3.0 | 0.06 |
| LONGHORN | 60 | 92.5 | 5.0 | 93.8 | 6.3 | 3.7 | 0.20 | 3.0 | 0.08 |
| NEOSHO | 60 | 91.3 | 2.5 | 92.4 | 2.8 | 3.7 | 0.10 | 3.0 | 0.14 |
| OGALLALA | 60 | 93.8 | 4.8 | 94.9 | 4.1 | 3.8 | 0.19 | 3.0 | 0.11 |
| POSTROCK | 60 | 93.8 | 2.5 | 97.5 | 2.9 | 3.8 | 0.10 | 3.0 | 0.08 |
| THUNDERBOLT | 60 | 91.3 | 6.3 | 93.8 | 8.6 | 3.9 | 0.32 | 2.8 | 0.17 |
| W04-417 | 60 | 96.3 | 2.5 | 98.8 | 2.5 | 3.9 | 0.10 | 3.0 | 0.08 |
| NUFRONTIER | 60 | 92.5 | 2.9 | 93.8 | 4.8 | 3.7 | 0.12 | 3.0 | 0.11 |
| NUHORIZON | 60 | 91.3 | 2.5 | 92.4 | 2.8 | 3.9 | 0.43 | 2.9 | 0.16 |
| ONAGA | 60 | 96.3 | 4.8 | 97.6 | 6.6 | 4.1 | 0.48 | 2.9 | 0.26 |
| RONL | 60 | 92.5 | 6.5 | 93.8 | 7.5 | 3.7 | 0.26 | 3.0 | 0.04 |
| 2145 | 60 | 90.0 | 0.0 | 91.2 | 2.4 | 3.8 | 0.45 | 2.8 | 0.13 |
| HEYNE | 60 | 91.3 | 4.8 | 95.1 | 9.3 | 3.7 | 0.19 | 2.9 | 0.11 |
| KS00F5-20-3 | 60 | 95.0 | 4.1 | 98.8 | 4.9 | 4.0 | 0.33 | 2.9 | 0.15 |
| OVERLEY | 60 | 95.0 | 4.1 | 96.3 | 6.4 | 3.8 | 0.16 | 3.0 | 0.05 |
| FULLER | 60 | 91.3 | 2.5 | 93.6 | 2.4 | 3.9 | 0.43 | 2.8 | 0.12 |
| COSSACK | 60 | 91.3 | 6.3 | 94.9 | 5.9 | 3.7 | 0.25 | 2.9 | 0.07 |
| ENHANCER | 60 | 90.0 | 7.1 | 91.1 | 5.1 | 3.6 | 0.28 | 2.9 | 0.11 |
| SANTA_FE | 60 | 95.0 | 4.1 | 96.3 | 6.4 | 3.8 | 0.16 | 3.0 | 0.08 |
| VENANGO | 60 | 91.3 | 4.8 | 92.4 | 3.0 | 3.7 | 0.19 | 2.9 | 0.03 |
| WB411W | 60 | 88.8 | 6.3 | 88.8 | 6.3 | 4.0 | 0.39 | 2.7 | 0.20 |
| KEOTA | 60 | 95.0 | 4.1 | 97.4 | 3.0 | 3.8 | 0.16 | 3.1 | 0.07 |
| TX05A001822 | 60 | 90.0 | 4.1 | 93.8 | 7.5 | 3.8 | 0.62 | 2.8 | 0.18 |
| TX06A001263 | 60 | 92.5 | 6.5 | 93.7 | 6.3 | 3.7 | 0.26 | 3.0 | 0.11 |
| TX06A001132 | 60 | 90.0 | 4.1 | 91.1 | 2.6 | 4.1 | 0.66 | 2.7 | 0.23 |
| TX06A001281 | 60 | 92.5 | 2.9 | 95.0 | 4.1 | 3.7 | 0.12 | 2.9 | 0.07 |
| TX06A001386 | 60 | 95.0 | 7.1 | 96.3 | 8.6 | 4.0 | 0.18 | 2.8 | 0.17 |
| TX05V7259 | 60 | 90.0 | 7.1 | 91.1 | 5.1 | 4.3 | 0.44 | 2.7 | 0.08 |
| TX05V7269 | 60 | 91.3 | 7.5 | 92.5 | 8.7 | 3.9 | 0.10 | 2.9 | 0.16 |
| TX05A001188 | 60 | 92.5 | 2.9 | 93.8 | 4.8 | 3.9 | 0.55 | 2.9 | 0.18 |
| TX07A001279 | 60 | 93.8 | 4.8 | 96.3 | 4.8 | 3.8 | 0.19 | 3.0 | 0.05 |
| TX07A001318 | 60 | 90.0 | 9.1 | 91.1 | 7.7 | 3.8 | 0.45 | 2.8 | 0.13 |
| TX07A001420 | 60 | 88.8 | 7.5 | 88.8 | 7.5 | 3.8 | 0.43 | 2.8 | 0.23 |
| TX06V7266 | 60 | 93.8 | 6.3 | 95.0 | 7.1 | 4.0 | 0.57 | 2.8 | 0.09 |
| OK1067071 | 60 | 91.3 | 4.8 | 94.9 | 4.3 | 3.9 | 0.60 | 2.8 | 0.22 |
| OK1067274 | 60 | 91.3 | 7.5 | 91.3 | 7.5 | 3.9 | 0.76 | 2.9 | 0.16 |
| OK1068002 | 60 | 86.3 | 10.3 | 87.2 | 9.0 | 4.2 | 0.57 | 2.8 | 0.34 |
| OK1068009 | 60 | 93.8 | 6.3 | 93.8 | 6.3 | 3.8 | 0.25 | 3.0 | 0.05 |
| OK1068026 | 60 | 93.8 | 6.3 | 95.0 | 7.1 | 3.8 | 0.25 | 3.0 | 0.08 |
| OK1068112 | 60 | 90.0 | 7.1 | 91.2 | 7.5 | 3.6 | 0.28 | 2.9 | 0.07 |
| OK1070275 | 60 | 97.5 | 2.9 | 97.5 | 2.9 | 3.9 | 0.12 | 3.0 | 0.06 |
| OK1070267 | 60 | 88.8 | 4.8 | 89.9 | 4.1 | 3.8 | 0.52 | 2.7 | 0.17 |
| OK09634 | 60 | 92.5 | 6.5 | 94.9 | 7.1 | 3.9 | 0.28 | 2.9 | 0.16 |
| OK10119 | 60 | 92.5 | 2.9 | 94.9 | 4.1 | 4.2 | 0.55 | 2.8 | 0.12 |
| GALLAGHER | 60 | 91.3 | 6.3 | 91.3 | 6.3 | 3.9 | 0.49 | 2.8 | 0.10 |
| OK07231 | 60 | 87.5 | 8.7 | 88.8 | 10.3 | 3.7 | 0.73 | 2.7 | 0.16 |
| OK07S117 | 60 | 91.3 | 4.8 | 91.3 | 4.8 | 3.7 | 0.19 | 2.9 | 0.03 |
| OK08328 | 60 | 87.5 | 6.5 | 88.8 | 8.5 | 3.5 | 0.26 | 2.9 | 0.09 |
| BIG_SKY | 60 | 93.8 | 2.5 | 93.8 | 2.5 | 3.8 | 0.10 | 2.9 | 0.08 |
| DANBY | 60 | 93.8 | 6.3 | 95.0 | 7.1 | 4.0 | 0.21 | 2.8 | 0.15 |
| E2041 | 60 | 95.0 | 4.1 | 97.8 | 9.2 | 3.8 | 0.16 | 3.0 | 0.03 |
| DENALI | 60 | 87.5 | 6.5 | 91.1 | 8.5 | 3.9 | 0.60 | 2.7 | 0.18 |
| CO050337-2 | 60 | 88.8 | 4.8 | 92.9 | 12.8 | 4.0 | 0.62 | 2.7 | 0.17 |
| BYRD | 60 | 93.8 | 6.3 | 95.0 | 7.1 | 3.8 | 0.25 | 2.9 | 0.07 |
| CO07W245 | 60 | 90.0 | 9.1 | 90.0 | 9.1 | 3.8 | 0.28 | 2.8 | 0.14 |
| MCGILL | 60 | 93.8 | 6.3 | 97.6 | 8.8 | 3.8 | 0.25 | 2.9 | 0.07 |
| NE02558 | 60 | 90.0 | 4.1 | 92.5 | 6.5 | 3.6 | 0.16 | 2.9 | 0.06 |
| NW03666 | 60 | 92.5 | 2.9 | 93.7 | 2.5 | 3.7 | 0.12 | 2.9 | 0.00 |
| NE04490 | 60 | 90.0 | 4.1 | 91.2 | 4.7 | 3.6 | 0.16 | 2.8 | 0.06 |
| NE05430 | 60 | 91.3 | 4.8 | 91.3 | 4.8 | 3.9 | 0.46 | 2.8 | 0.15 |
| NE05496 | 60 | 91.3 | 7.5 | 92.5 | 8.7 | 3.7 | 0.30 | 2.9 | 0.15 |
| NE05548 | 60 | 91.3 | 4.8 | 92.5 | 6.5 | 4.1 | 0.51 | 2.8 | 0.15 |
| NE06545 | 60 | 88.8 | 4.8 | 89.9 | 4.1 | 3.8 | 0.36 | 2.8 | 0.17 |
| NE06607 | 60 | 91.3 | 7.5 | 92.6 | 9.7 | 3.9 | 0.36 | 2.8 | 0.20 |
| ROBIDOUX | 60 | 88.8 | 4.8 | 88.8 | 4.8 | 4.0 | 0.62 | 2.7 | 0.17 |
| NI06736 | 60 | 93.8 | 6.3 | 94.9 | 4.3 | 4.0 | 0.21 | 2.8 | 0.15 |
| NI06737 | 60 | 91.3 | 4.8 | 95.1 | 8.4 | 3.7 | 0.19 | 2.9 | 0.09 |
| NI07703 | 60 | 92.5 | 5.0 | 94.9 | 0.3 | 3.7 | 0.20 | 2.9 | 0.07 |
| NI08707 | 60 | 91.3 | 7.5 | 93.6 | 6.3 | 3.9 | 0.36 | 3.0 | 0.26 |
| NI08708 | 60 | 92.5 | 5.0 | 96.4 | 8.7 | 3.7 | 0.20 | 2.9 | 0.09 |
| EVEREST | 60 | 91.3 | 7.5 | 93.9 | 10.5 | 3.7 | 0.30 | 2.9 | 0.07 |
| TRIUMPH64 | 120 | 65.0 | 5.8 | 65.9 | 7.0 | 2.4 | 0.04 | 3.5 | 0.29 |
| CHISHOLM | 120 | 86.3 | 4.8 | 88.4 | 2.8 | 3.2 | 0.19 | 3.1 | 0.20 |
| CUSTER | 120 | 38.8 | 7.5 | 40.2 | 6.7 | 1.6 | 0.18 | 3.4 | 0.44 |
| 2174-05 | 120 | 28.8 | 2.5 | 29.1 | 2.9 | 1.1 | 0.13 | 3.7 | 0.45 |
| INTRADA | 120 | 45.0 | 5.8 | 45.5 | 5.2 | 1.7 | 0.27 | 3.6 | 0.49 |
| OK101 | 120 | 41.3 | 6.3 | 42.3 | 6.1 | 1.7 | 0.07 | 3.6 | 0.49 |
| OK102 | 120 | 42.5 | 5.0 | 43.0 | 4.8 | 1.6 | 0.15 | 3.8 | 0.53 |
| ENDURANCE | 120 | 81.3 | 4.8 | 84.5 | 3.9 | 2.8 | 0.29 | 3.4 | 0.27 |
| DELIVER | 120 | 42.5 | 8.7 | 44.0 | 11.6 | 1.8 | 0.37 | 3.4 | 0.19 |
| OK_BULLET | 120 | 42.5 | 9.6 | 42.5 | 9.6 | 1.5 | 0.43 | 3.7 | 0.34 |
| CENTERFIELD | 120 | 60.0 | 7.1 | 60.9 | 8.0 | 1.9 | 0.21 | 4.0 | 0.11 |
| GUYMON | 120 | 86.3 | 2.5 | 87.4 | 2.7 | 4.3 | 0.13 | 2.5 | 0.06 |
| DUSTER | 120 | 60.0 | 4.1 | 61.8 | 7.3 | 2.1 | 0.11 | 3.8 | 0.16 |
| OK_RISING | 120 | 51.3 | 4.8 | 51.3 | 4.8 | 1.9 | 0.30 | 3.7 | 0.43 |
| OK02405 | 120 | 52.5 | 2.9 | 53.2 | 2.4 | 1.8 | 0.10 | 3.9 | 0.34 |
| PETE | 120 | 51.3 | 2.5 | 51.9 | 2.4 | 1.7 | 0.08 | 4.0 | 0.14 |
| BILLINGS | 120 | 56.3 | 2.5 | 58.7 | 4.7 | 1.9 | 0.08 | 4.0 | 0.11 |
| OK04505 | 120 | 68.8 | 4.8 | 68.8 | 4.8 | 2.2 | 0.09 | 3.9 | 0.48 |
| OK04525 | 120 | 47.5 | 10.4 | 47.5 | 10.4 | 2.0 | 0.52 | 3.3 | 0.15 |
| OK04507 | 120 | 87.5 | 2.9 | 87.5 | 2.9 | 2.8 | 0.27 | 3.8 | 0.17 |
| OK05830 | 120 | 53.8 | 2.5 | 55.3 | 4.5 | 1.7 | 0.20 | 4.1 | 0.24 |
| OK04111 | 120 | 41.3 | 10.3 | 41.7 | 10.0 | 1.5 | 0.23 | 3.8 | 0.49 |
| OK04415 | 120 | 45.0 | 9.1 | 45.0 | 9.1 | 1.8 | 0.14 | 3.5 | 0.33 |
| OK05711W | 120 | 68.8 | 4.8 | 70.7 | 6.7 | 2.4 | 0.19 | 3.6 | 0.18 |
| OK05723W | 120 | 70.0 | 4.1 | 70.0 | 4.1 | 2.5 | 0.27 | 3.5 | 0.31 |
| OK05108 | 120 | 60.0 | 4.1 | 60.9 | 5.6 | 2.7 | 0.40 | 3.2 | 0.32 |
| OK05122 | 120 | 53.8 | 4.8 | 55.3 | 6.1 | 2.0 | 0.36 | 3.8 | 0.47 |
| OK05526 | 120 | 48.8 | 2.5 | 48.8 | 2.5 | 1.6 | 0.08 | 4.1 | 0.13 |
| OK05134 | 120 | 46.3 | 13.1 | 46.8 | 13.1 | 1.5 | 0.44 | 4.1 | 0.06 |
| OK05303 | 120 | 47.5 | 5.0 | 48.1 | 4.7 | 1.6 | 0.17 | 4.1 | 0.29 |
| OK05312 | 120 | 55.0 | 4.1 | 55.7 | 3.1 | 1.8 | 0.14 | 4.2 | 0.14 |
| OK05511 | 120 | 47.5 | 6.5 | 48.2 | 6.9 | 1.6 | 0.22 | 3.7 | 0.17 |
| OK05204 | 120 | 48.8 | 6.3 | 49.4 | 6.6 | 1.6 | 0.21 | 4.1 | 0.16 |
| GARRISON | 120 | 70.0 | 0.0 | 71.9 | 3.9 | 2.3 | 0.00 | 4.1 | 0.06 |
| OK06114 | 120 | 47.5 | 2.9 | 49.7 | 6.5 | 1.6 | 0.10 | 4.4 | 0.16 |
| OK06210 | 120 | 82.5 | 6.5 | 82.5 | 6.5 | 2.8 | 0.22 | 3.7 | 0.27 |
| OK06319 | 120 | 50.0 | 4.1 | 50.7 | 4.3 | 1.7 | 0.14 | 4.2 | 0.15 |
| OK06318 | 120 | 58.8 | 2.5 | 58.8 | 2.5 | 2.1 | 0.24 | 4.0 | 0.39 |
| OK06336 | 120 | 51.3 | 2.5 | 52.6 | 3.1 | 1.7 | 0.08 | 4.0 | 0.21 |
| AGATE | 120 | 41.3 | 6.3 | 41.7 | 5.7 | 1.5 | 0.37 | 3.8 | 0.44 |
| ALLIANCE | 120 | 51.3 | 2.5 | 51.9 | 2.4 | 1.6 | 0.17 | 4.4 | 0.28 |
| ANTELOPE | 120 | 42.5 | 8.7 | 43.1 | 9.0 | 1.4 | 0.29 | 4.0 | 0.14 |
| ARAPAHOE | 120 | 38.8 | 8.5 | 39.4 | 9.7 | 1.4 | 0.27 | 3.9 | 0.32 |
| BENNETT | 120 | 50.0 | 0.0 | 50.0 | 0.0 | 2.1 | 0.83 | 3.6 | 0.60 |
| BUCKSKIN | 120 | 40.0 | 4.1 | 40.0 | 4.1 | 1.5 | 0.52 | 4.1 | 0.78 |
| CENTURK78 | 120 | 46.3 | 9.5 | 46.8 | 9.1 | 1.8 | 0.31 | 3.5 | 0.59 |
| CHEYENNE | 120 | 72.5 | 5.0 | 74.6 | 7.5 | 2.5 | 0.34 | 3.8 | 0.24 |
| COLT | 120 | 48.8 | 2.5 | 48.8 | 2.5 | 1.8 | 0.25 | 3.7 | 0.31 |
| COUGAR | 120 | 75.0 | 4.1 | 76.0 | 4.5 | 2.5 | 0.14 | 4.1 | 0.06 |
| CULVER | 120 | 57.5 | 2.9 | 58.3 | 4.0 | 2.0 | 0.27 | 3.9 | 0.30 |
| GAGE | 120 | 85.0 | 0.0 | 86.1 | 2.2 | 4.4 | 0.94 | 2.6 | 0.12 |
| GOODSTREAK | 120 | 86.3 | 2.5 | 86.3 | 2.5 | 2.9 | 0.08 | 3.8 | 0.08 |
| HALLAM | 120 | 86.3 | 2.5 | 88.8 | 7.5 | 2.8 | 0.13 | 3.8 | 0.30 |
| HARRY | 120 | 38.8 | 2.5 | 39.3 | 3.0 | 1.7 | 0.40 | 3.4 | 0.49 |
| HOMESTEAD | 120 | 73.8 | 2.5 | 73.8 | 2.5 | 2.5 | 0.08 | 3.9 | 0.09 |
| INFINITY_CL | 120 | 50.0 | 8.2 | 50.7 | 8.3 | 1.7 | 0.27 | 3.8 | 0.07 |
| KHARKOF | 120 | 68.8 | 4.8 | 68.8 | 4.8 | 2.3 | 0.16 | 4.0 | 0.14 |
| MILLENNIUM | 120 | 45.0 | 8.2 | 45.0 | 8.2 | 1.8 | 0.33 | 3.5 | 0.37 |
| CAMELOT | 120 | 42.5 | 9.6 | 42.9 | 8.9 | 1.6 | 0.33 | 3.8 | 0.48 |
| OVERLAND | 120 | 48.8 | 8.5 | 49.3 | 8.3 | 1.6 | 0.28 | 3.8 | 0.14 |
| NE99495 | 120 | 83.8 | 4.8 | 83.8 | 4.8 | 2.7 | 0.31 | 3.8 | 0.11 |
| NIOBRARA | 120 | 83.8 | 6.3 | 84.7 | 4.5 | 2.8 | 0.21 | 3.8 | 0.14 |
| NUPLAINS | 120 | 71.3 | 2.5 | 72.2 | 2.6 | 2.5 | 0.22 | 4.0 | 0.41 |
| PRONGHORN | 120 | 48.8 | 2.5 | 49.4 | 3.2 | 1.6 | 0.08 | 4.1 | 0.36 |
| RAWHIDE | 120 | 58.8 | 2.5 | 59.5 | 3.4 | 2.0 | 0.08 | 4.0 | 0.34 |
| REDLAND | 120 | 53.8 | 4.8 | 54.5 | 5.2 | 2.0 | 0.22 | 3.6 | 0.28 |
| SCOUT66 | 120 | 71.3 | 4.8 | 71.3 | 4.8 | 2.4 | 0.16 | 3.9 | 0.19 |
| SIOUXLAND | 120 | 56.3 | 8.5 | 57.6 | 7.4 | 2.2 | 0.48 | 3.6 | 0.28 |
| TURKEY_NEBSEL | 120 | 67.5 | 2.9 | 69.3 | 3.6 | 2.2 | 0.22 | 3.9 | 0.16 |
| VISTA | 120 | 58.8 | 4.8 | 60.4 | 6.6 | 2.4 | 0.19 | 3.4 | 0.02 |
| WAHOO | 120 | 56.3 | 2.5 | 56.3 | 2.5 | 1.9 | 0.08 | 3.9 | 0.29 |
| WARRIOR | 120 | 67.5 | 6.5 | 67.5 | 6.5 | 2.3 | 0.22 | 3.9 | 0.09 |
| WESLEY | 120 | 85.0 | 4.1 | 86.2 | 6.2 | 2.8 | 0.14 | 4.0 | 0.15 |
| WICHITA | 120 | 67.5 | 2.9 | 67.5 | 2.9 | 2.3 | 0.10 | 3.9 | 0.11 |
| WINDSTAR | 120 | 57.5 | 6.5 | 58.3 | 7.0 | 1.9 | 0.28 | 3.8 | 0.17 |
| LANCER | 120 | 63.8 | 2.5 | 63.8 | 2.5 | 2.0 | 0.15 | 4.3 | 0.23 |
| ANTON | 120 | 53.8 | 2.5 | 55.1 | 0.3 | 1.9 | 0.23 | 3.9 | 0.30 |
| MACE | 120 | 46.3 | 10.3 | 46.7 | 9.7 | 1.7 | 0.49 | 3.7 | 0.50 |
| TAM107-R7 | 120 | 46.3 | 2.5 | 46.8 | 2.4 | 1.6 | 0.15 | 3.8 | 0.28 |
| ARLIN | 120 | 51.3 | 6.3 | 53.6 | 9.3 | 1.7 | 0.21 | 4.0 | 0.21 |
| ALICE | 120 | 55.0 | 4.1 | 55.0 | 4.1 | 1.9 | 0.23 | 4.0 | 0.24 |
| DARRELL | 120 | 72.5 | 2.9 | 73.5 | 4.3 | 2.4 | 0.10 | 3.9 | 0.11 |
| EXPEDITION | 120 | 66.3 | 2.5 | 68.0 | 2.1 | 2.2 | 0.08 | 3.9 | 0.15 |
| WENDY | 120 | 85.0 | 7.1 | 85.0 | 7.1 | 2.6 | 0.37 | 4.0 | 0.34 |
| SD00111-9 | 120 | 52.5 | 8.7 | 53.9 | 9.7 | 2.0 | 0.46 | 3.9 | 0.68 |
| SD01237 | 120 | 72.5 | 2.9 | 73.4 | 2.4 | 2.4 | 0.10 | 3.9 | 0.20 |
| SD01058 | 120 | 48.8 | 2.5 | 49.4 | 3.2 | 1.9 | 0.28 | 3.6 | 0.60 |
| SD05118 | 120 | 77.5 | 6.5 | 77.5 | 6.5 | 2.6 | 0.22 | 4.2 | 0.08 |
| SD05210 | 120 | 55.0 | 4.1 | 57.2 | 5.4 | 2.2 | 0.16 | 3.5 | 0.06 |
| SD05W018 | 120 | 72.5 | 5.0 | 74.3 | 4.4 | 2.4 | 0.20 | 4.1 | 0.43 |
| NEKOTA | 120 | 62.5 | 2.9 | 63.3 | 2.4 | 2.1 | 0.10 | 4.1 | 0.18 |
| TANDEM | 120 | 72.5 | 2.9 | 74.4 | 3.2 | 2.4 | 0.10 | 3.9 | 0.20 |
| CRIMSON | 120 | 58.8 | 2.5 | 58.8 | 2.5 | 2.0 | 0.08 | 4.0 | 0.16 |
| ROSE | 120 | 36.3 | 6.3 | 37.3 | 7.3 | 1.5 | 0.26 | 3.5 | 0.41 |
| DAWN | 120 | 72.5 | 5.0 | 74.6 | 7.5 | 2.4 | 0.17 | 3.9 | 0.18 |
| WINOKA | 120 | 36.3 | 9.5 | 37.1 | 9.1 | 1.4 | 0.22 | 3.9 | 0.13 |
| NELL | 120 | 73.8 | 7.5 | 74.7 | 7.1 | 2.5 | 0.25 | 4.0 | 0.13 |
| RITA | 120 | 72.5 | 2.9 | 73.5 | 4.3 | 2.4 | 0.10 | 4.0 | 0.09 |
| BRONZE | 120 | 51.3 | 2.5 | 52.0 | 3.9 | 1.7 | 0.08 | 4.1 | 0.21 |
| HUME | 120 | 48.8 | 6.3 | 50.3 | 8.6 | 1.8 | 0.16 | 3.7 | 0.45 |
| GENT | 120 | 51.3 | 2.5 | 53.3 | 2.5 | 2.1 | 0.10 | 3.1 | 0.05 |
| HARDING | 120 | 56.3 | 4.8 | 56.3 | 4.8 | 1.9 | 0.16 | 4.1 | 0.29 |
| HV9W03-1551WP | 120 | 43.8 | 4.8 | 43.8 | 4.8 | 1.5 | 0.14 | 3.8 | 0.41 |
| G1878 | 120 | 58.8 | 2.5 | 58.8 | 2.5 | 2.0 | 0.08 | 4.0 | 0.20 |
| HV9W03-1379R | 120 | 47.5 | 5.0 | 48.2 | 5.6 | 1.6 | 0.17 | 4.1 | 0.21 |
| HV9W03-1596R | 120 | 72.5 | 2.9 | 73.5 | 4.3 | 2.4 | 0.10 | 3.9 | 0.22 |
| HV9W05-1280R | 120 | 47.5 | 5.0 | 48.8 | 4.8 | 1.6 | 0.17 | 4.1 | 0.19 |
| HV9W06-504 | 120 | 71.3 | 2.5 | 72.2 | 4.5 | 2.7 | 0.44 | 3.5 | 0.19 |
| SPARTAN | 120 | 63.8 | 2.5 | 64.6 | 3.5 | 2.0 | 0.15 | 3.9 | 0.15 |
| HV906-865 | 120 | 66.3 | 2.5 | 66.3 | 2.5 | 2.0 | 0.15 | 4.0 | 0.14 |
| TARKIO | 120 | 50.0 | 7.1 | 50.6 | 6.6 | 1.7 | 0.24 | 3.9 | 0.18 |
| SMOKYHILL | 120 | 56.3 | 2.5 | 60.1 | 4.4 | 1.9 | 0.08 | 4.1 | 0.13 |
| SHOCKER | 120 | 68.8 | 4.8 | 69.7 | 5.4 | 2.3 | 0.16 | 4.2 | 0.16 |
| VONA | 120 | 53.8 | 4.8 | 53.8 | 4.8 | 1.8 | 0.16 | 4.1 | 0.21 |
| CO940610 | 120 | 63.8 | 4.8 | 63.8 | 4.8 | 2.1 | 0.16 | 3.8 | 0.08 |
| AVALANCHE | 120 | 61.3 | 8.5 | 62.2 | 9.9 | 3.2 | 1.01 | 3.0 | 0.18 |
| BOND_CL | 120 | 58.8 | 2.5 | 58.8 | 2.5 | 2.0 | 0.08 | 3.8 | 0.13 |
| PLATTE | 120 | 51.3 | 6.3 | 51.8 | 5.6 | 1.7 | 0.21 | 4.0 | 0.19 |
| LINDON | 120 | 47.5 | 5.0 | 48.2 | 6.4 | 1.6 | 0.26 | 3.9 | 0.49 |
| CO03W043 | 120 | 72.5 | 5.0 | 72.5 | 5.0 | 2.6 | 0.49 | 3.6 | 0.44 |
| SNOWMASS | 120 | 75.0 | 4.1 | 78.1 | 7.2 | 2.5 | 0.14 | 3.9 | 0.20 |
| THUNDER_CL | 120 | 72.5 | 2.9 | 74.6 | 6.3 | 2.4 | 0.10 | 3.9 | 0.10 |
| CO04025 | 120 | 67.5 | 2.9 | 69.2 | 0.9 | 2.3 | 0.10 | 3.9 | 0.07 |
| CO04393 | 120 | 73.8 | 2.5 | 75.8 | 5.5 | 2.5 | 0.08 | 3.9 | 0.15 |
| CO04499 | 120 | 71.3 | 4.8 | 71.3 | 4.8 | 2.4 | 0.16 | 4.1 | 0.06 |
| CO04W320 | 120 | 36.3 | 2.5 | 36.7 | 2.4 | 1.4 | 0.25 | 3.4 | 0.50 |
| LAMAR | 120 | 77.5 | 5.0 | 77.5 | 5.0 | 2.5 | 0.04 | 4.0 | 0.06 |
| CARSON | 120 | 36.3 | 2.5 | 36.8 | 3.6 | 1.5 | 0.19 | 3.3 | 0.44 |
| HAIL | 120 | 37.5 | 2.9 | 38.5 | 3.2 | 1.3 | 0.10 | 3.7 | 0.15 |
| SANDY | 120 | 51.3 | 4.8 | 51.3 | 4.8 | 1.7 | 0.16 | 4.0 | 0.23 |
| DUKE | 120 | 48.8 | 2.5 | 50.8 | 4.5 | 1.6 | 0.08 | 4.1 | 0.16 |
| HALT | 120 | 57.5 | 2.9 | 57.5 | 2.9 | 1.9 | 0.10 | 4.0 | 0.21 |
| HATCHER | 120 | 36.3 | 6.3 | 36.3 | 6.3 | 1.4 | 0.16 | 3.6 | 0.51 |
| PRAIRIE_RED | 120 | 63.8 | 10.3 | 65.6 | 11.2 | 2.1 | 0.34 | 3.8 | 0.21 |
| ABOVE | 120 | 71.3 | 4.8 | 71.3 | 4.8 | 2.4 | 0.16 | 3.9 | 0.16 |
| CO03064 | 120 | 57.5 | 2.9 | 59.1 | 4.7 | 2.1 | 0.25 | 3.5 | 0.29 |
| BILL_BROWN | 120 | 36.3 | 2.5 | 36.8 | 3.6 | 1.4 | 0.25 | 3.7 | 0.68 |
| RIPPER | 120 | 35.0 | 0.0 | 35.9 | 1.1 | 1.4 | 0.28 | 3.5 | 0.58 |
| PROWERS | 120 | 78.8 | 2.5 | 79.7 | 0.5 | 2.6 | 0.08 | 3.9 | 0.15 |
| AKRON | 120 | 47.5 | 2.9 | 47.5 | 2.9 | 1.6 | 0.10 | 4.1 | 0.23 |
| JULES | 120 | 61.3 | 4.8 | 61.3 | 4.8 | 2.0 | 0.15 | 3.9 | 0.15 |
| YUMA | 120 | 62.5 | 5.0 | 64.1 | 4.4 | 2.1 | 0.17 | 4.2 | 0.10 |
| TAMW-101 | 120 | 72.5 | 2.9 | 74.6 | 6.3 | 2.4 | 0.10 | 4.1 | 0.11 |
| TAM105 | 120 | 66.3 | 2.5 | 67.2 | 4.3 | 2.2 | 0.08 | 4.0 | 0.07 |
| TAM107 | 120 | 50.0 | 4.1 | 50.6 | 3.2 | 1.9 | 0.57 | 4.1 | 0.83 |
| TAM109 | 120 | 75.0 | 4.1 | 76.0 | 4.5 | 2.4 | 0.11 | 4.0 | 0.27 |
| TAM110 | 120 | 75.0 | 0.0 | 76.0 | 2.0 | 2.5 | 0.00 | 3.8 | 0.18 |
| TAM111 | 120 | 57.5 | 2.9 | 59.9 | 5.0 | 1.9 | 0.10 | 4.0 | 0.21 |
| TAM112 | 120 | 76.3 | 2.5 | 77.2 | 2.6 | 2.5 | 0.08 | 4.0 | 0.14 |
| TAM200 | 120 | 57.5 | 2.9 | 58.2 | 2.4 | 2.0 | 0.27 | 3.9 | 0.22 |
| TAM202 | 120 | 28.8 | 2.5 | 30.0 | 3.6 | 1.6 | 0.48 | 3.0 | 0.71 |
| TAM203 | 120 | 48.8 | 2.5 | 50.7 | 3.4 | 1.6 | 0.08 | 3.8 | 0.16 |
| TAM302 | 120 | 82.5 | 6.5 | 82.5 | 6.5 | 2.6 | 0.25 | 3.8 | 0.15 |
| TAM303 | 120 | 37.5 | 2.9 | 37.5 | 2.9 | 2.1 | 0.74 | 2.9 | 0.65 |
| TAM304 | 120 | 66.3 | 6.3 | 67.2 | 8.2 | 2.2 | 0.21 | 4.0 | 0.17 |
| TAM400 | 120 | 35.0 | 4.1 | 35.9 | 4.4 | 1.4 | 0.39 | 3.6 | 0.55 |
| LOCKETT | 120 | 57.5 | 2.9 | 57.5 | 2.9 | 2.0 | 0.15 | 3.8 | 0.27 |
| STURDY | 120 | 30.0 | 0.0 | 30.8 | 0.9 | 1.5 | 0.41 | 3.2 | 0.27 |
| STURDY_2K | 120 | 53.8 | 2.5 | 54.4 | 1.2 | 1.8 | 0.08 | 3.7 | 0.20 |
| MIT | 120 | 47.5 | 2.9 | 48.8 | 3.3 | 1.9 | 0.44 | 3.7 | 0.44 |
| CAPROCK | 120 | 71.3 | 2.5 | 73.2 | 3.9 | 2.4 | 0.08 | 3.8 | 0.23 |
| TX01A5936 | 120 | 70.0 | 4.1 | 70.0 | 4.1 | 2.3 | 0.14 | 4.1 | 0.18 |
| TAM401 | 120 | 75.0 | 4.1 | 75.0 | 4.1 | 2.5 | 0.14 | 4.1 | 0.14 |
| TX02A0252 | 120 | 36.3 | 9.5 | 37.1 | 9.1 | 1.5 | 0.34 | 3.4 | 0.73 |
| TX03A0148 | 120 | 35.0 | 0.0 | 35.0 | 0.0 | 1.5 | 0.29 | 3.3 | 0.21 |
| TX03A0563 | 120 | 37.5 | 2.9 | 38.0 | 2.5 | 1.5 | 0.36 | 3.7 | 0.51 |
| TX04A001246 | 120 | 75.0 | 4.1 | 79.0 | 4.0 | 2.7 | 0.21 | 3.5 | 0.26 |
| TX01V5134RC-3 | 120 | 86.3 | 4.8 | 86.3 | 4.8 | 2.9 | 0.16 | 3.7 | 0.09 |
| TX04M410164 | 120 | 57.5 | 2.9 | 58.2 | 2.4 | 1.9 | 0.10 | 3.9 | 0.16 |
| TX04M410211 | 120 | 38.8 | 2.5 | 39.2 | 1.6 | 1.4 | 0.13 | 3.3 | 0.25 |
| TX04V075080 | 120 | 36.3 | 4.8 | 37.2 | 4.8 | 1.4 | 0.50 | 3.7 | 0.78 |
| TX99A0153-1 | 120 | 47.5 | 2.9 | 47.5 | 2.9 | 1.6 | 0.10 | 4.1 | 0.38 |
| TX01M5009-28 | 120 | 52.5 | 2.9 | 53.2 | 2.4 | 2.1 | 0.48 | 3.4 | 0.28 |
| TX00V1131 | 120 | 48.8 | 2.5 | 50.7 | 2.5 | 1.8 | 0.25 | 3.8 | 0.48 |
| TX99U8618 | 120 | 83.8 | 6.3 | 83.8 | 6.3 | 2.8 | 0.21 | 3.8 | 0.15 |
| TX96D1073 | 120 | 62.5 | 2.9 | 63.4 | 4.1 | 2.1 | 0.10 | 4.0 | 0.11 |
| 2180 | 120 | 47.5 | 2.9 | 50.3 | 6.0 | 1.7 | 0.12 | 3.9 | 0.38 |
| HG-9 | 120 | 37.5 | 6.5 | 38.9 | 6.6 | 1.4 | 0.16 | 3.9 | 0.60 |
| TX86A5606 | 120 | 58.8 | 4.8 | 59.5 | 4.2 | 2.5 | 0.24 | 3.3 | 0.23 |
| TX86A8072 | 120 | 66.3 | 4.8 | 66.3 | 4.8 | 2.1 | 0.16 | 4.0 | 0.29 |
| CREST | 120 | 52.5 | 2.9 | 52.5 | 2.9 | 1.8 | 0.10 | 3.9 | 0.05 |
| ROSEBUD | 120 | 58.8 | 4.8 | 59.6 | 6.3 | 2.0 | 0.16 | 3.8 | 0.04 |
| JUDITH | 120 | 63.8 | 2.5 | 64.6 | 3.5 | 2.1 | 0.08 | 4.0 | 0.14 |
| MT85200 | 120 | 65.0 | 8.2 | 65.0 | 8.2 | 2.2 | 0.27 | 3.6 | 0.17 |
| NUSKY | 120 | 38.8 | 4.8 | 39.8 | 5.5 | 1.7 | 0.27 | 3.3 | 0.42 |
| MT9513 | 120 | 77.5 | 2.9 | 78.6 | 4.4 | 2.6 | 0.10 | 3.7 | 0.11 |
| MT9904 | 120 | 72.5 | 5.0 | 74.4 | 5.2 | 2.4 | 0.17 | 3.7 | 0.31 |
| NORRIS | 120 | 72.5 | 2.9 | 73.4 | 2.4 | 2.4 | 0.10 | 3.8 | 0.19 |
| YELLOWSTONE | 120 | 58.8 | 2.5 | 58.8 | 2.5 | 2.1 | 0.10 | 3.8 | 0.31 |
| MT0495 | 120 | 40.0 | 7.1 | 40.4 | 6.3 | 1.5 | 0.41 | 3.7 | 0.47 |
| MTS0531 | 120 | 87.5 | 6.5 | 88.6 | 6.3 | 4.8 | 1.06 | 2.5 | 0.08 |
| DECADE | 120 | 71.3 | 8.5 | 73.0 | 8.0 | 2.9 | 0.76 | 3.6 | 0.59 |
| MT06103 | 120 | 68.8 | 2.5 | 71.5 | 1.8 | 2.4 | 0.27 | 3.8 | 0.40 |
| JUDEE | 120 | 47.5 | 2.9 | 48.1 | 2.4 | 1.5 | 0.16 | 4.2 | 0.24 |
| LAKIN | 120 | 67.5 | 6.5 | 68.3 | 5.3 | 2.2 | 0.14 | 4.1 | 0.06 |
| STANTON | 120 | 60.0 | 4.1 | 60.7 | 3.0 | 2.0 | 0.14 | 4.1 | 0.16 |
| TREGO | 120 | 73.8 | 2.5 | 74.7 | 3.7 | 3.0 | 0.10 | 3.3 | 0.11 |
| KARL_92 | 120 | 73.8 | 6.3 | 74.6 | 4.7 | 2.5 | 0.21 | 4.0 | 0.15 |
| DODGE | 120 | 76.3 | 2.5 | 79.5 | 6.6 | 2.5 | 0.08 | 4.0 | 0.14 |
| NORKAN | 120 | 56.3 | 2.5 | 57.0 | 2.4 | 2.1 | 0.64 | 3.9 | 0.75 |
| CHENEY | 120 | 36.3 | 4.8 | 36.6 | 4.1 | 1.5 | 0.36 | 3.5 | 0.77 |
| NEWTON | 120 | 63.8 | 4.8 | 64.5 | 4.2 | 2.1 | 0.16 | 4.0 | 0.13 |
| LARNED | 120 | 75.0 | 4.1 | 76.0 | 4.5 | 2.5 | 0.14 | 4.1 | 0.13 |
| PARKER76 | 120 | 70.0 | 4.1 | 71.0 | 5.8 | 2.3 | 0.14 | 4.1 | 0.17 |
| KIRWIN | 120 | 77.5 | 2.9 | 78.6 | 4.4 | 2.6 | 0.10 | 3.9 | 0.14 |
| SAGE | 120 | 65.0 | 4.1 | 65.9 | 5.7 | 2.2 | 0.14 | 4.2 | 0.10 |
| TRISON | 120 | 57.5 | 5.0 | 60.0 | 8.2 | 1.9 | 0.17 | 4.0 | 0.23 |
| EAGLE | 120 | 52.5 | 5.0 | 53.8 | 4.3 | 2.0 | 0.39 | 3.7 | 0.57 |
| SHAWNEE | 120 | 57.5 | 2.9 | 58.3 | 4.0 | 2.1 | 0.41 | 3.8 | 0.72 |
| PARKER | 120 | 72.5 | 6.5 | 73.4 | 5.2 | 2.4 | 0.22 | 3.8 | 0.17 |
| KAW61 | 120 | 72.5 | 2.9 | 75.6 | 5.1 | 2.9 | 0.12 | 3.4 | 0.13 |
| TASCOSA | 120 | 86.3 | 2.5 | 88.6 | 4.6 | 4.1 | 0.33 | 2.6 | 0.24 |
| BISON | 120 | 75.0 | 4.1 | 76.9 | 3.0 | 2.5 | 0.14 | 4.0 | 0.25 |
| KIOWA | 120 | 73.8 | 2.5 | 74.7 | 3.7 | 2.5 | 0.08 | 4.2 | 0.07 |
| WICHITA | 120 | 66.3 | 2.5 | 67.2 | 4.3 | 2.2 | 0.08 | 4.1 | 0.20 |
| COMANCHE | 120 | 72.5 | 6.5 | 74.3 | 4.8 | 2.4 | 0.22 | 3.9 | 0.17 |
| BAKERS_WHITE | 120 | 48.8 | 4.8 | 51.5 | 6.5 | 1.6 | 0.16 | 4.1 | 0.24 |
| BURCHETT | 120 | 36.3 | 4.8 | 36.7 | 4.7 | 1.5 | 0.36 | 3.5 | 0.60 |
| CUTTER | 120 | 72.5 | 2.9 | 73.5 | 4.3 | 2.9 | 0.12 | 3.6 | 0.17 |
| DUMAS | 120 | 57.5 | 5.0 | 59.9 | 6.4 | 1.9 | 0.24 | 4.2 | 0.42 |
| HONDO | 120 | 41.3 | 2.5 | 41.8 | 2.4 | 2.0 | 0.55 | 3.2 | 0.58 |
| JAGALENE | 120 | 66.3 | 4.8 | 67.1 | 4.8 | 2.0 | 0.20 | 4.2 | 0.11 |
| LONGHORN | 120 | 57.5 | 2.9 | 58.3 | 4.0 | 1.9 | 0.10 | 4.0 | 0.40 |
| NEOSHO | 120 | 73.8 | 2.5 | 74.7 | 3.7 | 2.5 | 0.08 | 3.9 | 0.08 |
| OGALLALA | 120 | 53.8 | 4.8 | 54.4 | 4.3 | 1.7 | 0.19 | 4.5 | 0.71 |
| POSTROCK | 120 | 40.0 | 4.1 | 41.6 | 3.9 | 1.7 | 0.32 | 3.3 | 0.59 |
| THUNDERBOLT | 120 | 75.0 | 0.0 | 77.0 | 2.3 | 2.5 | 0.00 | 4.1 | 0.08 |
| W04-417 | 120 | 36.3 | 6.3 | 37.1 | 5.7 | 1.7 | 0.64 | 3.3 | 0.59 |
| NUFRONTIER | 120 | 55.0 | 8.2 | 55.7 | 8.3 | 1.8 | 0.25 | 4.1 | 0.45 |
| NUHORIZON | 120 | 66.3 | 4.8 | 67.1 | 4.8 | 2.2 | 0.16 | 3.9 | 0.10 |
| ONAGA | 120 | 60.0 | 9.1 | 60.7 | 8.2 | 2.2 | 0.39 | 3.7 | 0.43 |
| RONL | 120 | 62.5 | 2.9 | 63.4 | 4.1 | 2.1 | 0.10 | 4.3 | 0.15 |
| 2145 | 120 | 48.8 | 2.5 | 49.4 | 3.2 | 1.9 | 0.25 | 3.7 | 0.49 |
| HEYNE | 120 | 60.0 | 4.1 | 62.6 | 7.2 | 2.0 | 0.22 | 4.1 | 0.59 |
| KS00F5-20-3 | 120 | 43.8 | 6.3 | 45.3 | 4.7 | 1.8 | 0.32 | 3.4 | 0.72 |
| OVERLEY | 120 | 42.5 | 8.7 | 43.0 | 8.5 | 1.6 | 0.22 | 3.7 | 0.50 |
| FULLER | 120 | 72.5 | 5.0 | 74.3 | 4.4 | 2.5 | 0.34 | 3.9 | 0.22 |
| COSSACK | 120 | 75.0 | 0.0 | 78.1 | 4.0 | 2.5 | 0.00 | 3.8 | 0.14 |
| ENHANCER | 120 | 75.0 | 4.1 | 75.9 | 2.8 | 2.5 | 0.14 | 4.0 | 0.20 |
| SANTA_FE | 120 | 62.5 | 2.9 | 63.3 | 2.4 | 2.1 | 0.10 | 4.1 | 0.13 |
| VENANGO | 120 | 71.3 | 2.5 | 72.2 | 2.6 | 2.4 | 0.08 | 4.1 | 0.19 |
| WB411W | 120 | 72.5 | 2.9 | 72.5 | 2.9 | 2.3 | 0.15 | 4.0 | 0.20 |
| KEOTA | 120 | 67.5 | 6.5 | 69.3 | 7.8 | 2.2 | 0.30 | 4.0 | 0.13 |
| TX05A001822 | 120 | 43.8 | 4.8 | 45.7 | 6.6 | 1.8 | 0.60 | 3.6 | 0.75 |
| TX06A001263 | 120 | 50.0 | 4.1 | 50.6 | 3.2 | 1.7 | 0.14 | 4.1 | 0.05 |
| TX06A001132 | 120 | 57.5 | 5.0 | 58.2 | 4.7 | 2.0 | 0.20 | 3.9 | 0.27 |
| TX06A001281 | 120 | 67.5 | 6.5 | 69.3 | 6.5 | 2.2 | 0.34 | 4.2 | 0.11 |
| TX06A001386 | 120 | 61.3 | 6.3 | 62.0 | 5.4 | 2.0 | 0.21 | 4.0 | 0.14 |
| TX05V7259 | 120 | 70.0 | 4.1 | 71.0 | 5.8 | 2.3 | 0.22 | 4.2 | 0.23 |
| TX05V7269 | 120 | 43.8 | 4.8 | 44.3 | 4.3 | 1.4 | 0.22 | 4.2 | 0.37 |
| TX05A001188 | 120 | 43.8 | 2.5 | 44.3 | 3.1 | 1.5 | 0.19 | 4.1 | 0.45 |
| TX07A001279 | 120 | 63.8 | 7.5 | 65.4 | 7.1 | 2.1 | 0.25 | 4.0 | 0.18 |
| TX07A001318 | 120 | 72.5 | 2.9 | 73.4 | 2.4 | 2.4 | 0.10 | 3.9 | 0.19 |
| TX07A001420 | 120 | 72.5 | 2.9 | 72.5 | 2.9 | 2.4 | 0.10 | 4.0 | 0.07 |
| TX06V7266 | 120 | 71.3 | 2.5 | 72.2 | 2.6 | 2.4 | 0.08 | 4.1 | 0.11 |
| OK1067071 | 120 | 50.0 | 4.1 | 52.1 | 6.1 | 1.6 | 0.08 | 4.0 | 0.18 |
| OK1067274 | 120 | 70.0 | 4.1 | 70.0 | 4.1 | 2.3 | 0.14 | 3.9 | 0.16 |
| OK1068002 | 120 | 76.3 | 8.5 | 77.1 | 7.1 | 2.6 | 0.23 | 3.8 | 0.43 |
| OK1068009 | 120 | 71.3 | 4.8 | 71.3 | 4.8 | 2.4 | 0.16 | 4.1 | 0.09 |
| OK1068026 | 120 | 48.8 | 4.8 | 49.3 | 4.3 | 2.3 | 0.31 | 3.1 | 0.05 |
| OK1068112 | 120 | 45.0 | 4.1 | 45.5 | 3.3 | 1.7 | 0.40 | 3.6 | 0.64 |
| OK1070275 | 120 | 42.5 | 5.0 | 42.5 | 5.0 | 1.6 | 0.32 | 3.6 | 0.45 |
| OK1070267 | 120 | 72.5 | 2.9 | 73.5 | 4.3 | 2.5 | 0.32 | 3.8 | 0.17 |
| OK09634 | 120 | 48.8 | 2.5 | 50.0 | 2.1 | 1.8 | 0.45 | 3.9 | 0.67 |
| OK10119 | 120 | 42.5 | 6.5 | 43.5 | 5.5 | 1.6 | 0.48 | 3.6 | 0.65 |
| GALLAGHER | 120 | 73.8 | 4.8 | 73.8 | 4.8 | 2.4 | 0.28 | 3.9 | 0.47 |
| OK07231 | 120 | 71.3 | 4.8 | 72.1 | 3.4 | 2.4 | 0.16 | 4.0 | 0.09 |
| OK07S117 | 120 | 66.3 | 4.8 | 66.3 | 4.8 | 2.2 | 0.16 | 4.0 | 0.16 |
| OK08328 | 120 | 62.5 | 6.5 | 63.4 | 8.0 | 2.1 | 0.51 | 3.9 | 0.45 |
| BIG_SKY | 120 | 71.3 | 2.5 | 71.3 | 2.5 | 2.4 | 0.08 | 3.8 | 0.14 |
| DANBY | 120 | 68.8 | 6.3 | 69.5 | 4.9 | 2.3 | 0.21 | 3.9 | 0.13 |
| E2041 | 120 | 46.3 | 6.3 | 47.5 | 6.5 | 1.9 | 0.55 | 3.5 | 0.53 |
| DENALI | 120 | 73.8 | 6.3 | 76.5 | 3.6 | 2.4 | 0.17 | 3.9 | 0.26 |
| CO050337-2 | 120 | 75.0 | 0.0 | 78.3 | 6.6 | 2.5 | 0.00 | 3.9 | 0.14 |
| BYRD | 120 | 70.0 | 0.0 | 70.9 | 1.8 | 2.3 | 0.17 | 4.1 | 0.60 |
| CO07W245 | 120 | 71.3 | 4.8 | 71.3 | 4.8 | 2.4 | 0.16 | 3.8 | 0.12 |
| MCGILL | 120 | 52.5 | 5.0 | 54.5 | 4.3 | 1.8 | 0.17 | 3.7 | 0.16 |
| NE02558 | 120 | 57.5 | 5.0 | 59.0 | 4.9 | 2.1 | 0.37 | 3.5 | 0.35 |
| NW03666 | 120 | 66.3 | 2.5 | 67.2 | 4.3 | 2.2 | 0.08 | 4.0 | 0.22 |
| NE04490 | 120 | 73.8 | 2.5 | 74.7 | 0.7 | 2.5 | 0.08 | 3.9 | 0.19 |
| NE05430 | 120 | 48.8 | 6.3 | 48.8 | 6.3 | 1.9 | 0.62 | 3.8 | 0.70 |
| NE05496 | 120 | 67.5 | 2.9 | 68.4 | 4.2 | 2.3 | 0.10 | 4.0 | 0.19 |
| NE05548 | 120 | 57.5 | 2.9 | 58.3 | 4.0 | 1.9 | 0.20 | 4.4 | 0.56 |
| NE06545 | 120 | 52.5 | 2.9 | 53.2 | 2.4 | 2.0 | 0.37 | 3.7 | 0.52 |
| NE06607 | 120 | 67.5 | 2.9 | 68.4 | 4.2 | 2.3 | 0.10 | 4.0 | 0.17 |
| ROBIDOUX | 120 | 51.3 | 6.3 | 51.3 | 6.3 | 2.0 | 0.24 | 3.7 | 0.58 |
| NI06736 | 120 | 45.0 | 4.1 | 45.7 | 5.2 | 1.6 | 0.20 | 3.8 | 0.36 |
| NI06737 | 120 | 62.5 | 5.0 | 65.0 | 4.3 | 2.0 | 0.25 | 4.1 | 0.58 |
| NI07703 | 120 | 73.8 | 2.5 | 75.8 | 5.5 | 2.5 | 0.08 | 3.9 | 0.22 |
| NI08707 | 120 | 63.8 | 4.8 | 65.7 | 8.4 | 2.1 | 0.16 | 4.1 | 0.19 |
| NI08708 | 120 | 71.3 | 4.8 | 74.2 | 6.7 | 2.3 | 0.17 | 3.9 | 0.28 |
| EVEREST | 120 | 70.0 | 5.8 | 72.1 | 8.9 | 2.3 | 0.19 | 4.0 | 0.30 |
