# Supplementary material for: Response of winter wheat genotypes to salinity stress under controlled environments
Source: Front Plant Sci. 2024 Jun 24;15:1396498. doi: 10.3389/fpls.2024.1396498 (PMC11228282; doi:10.3389/fpls.2024.1396498)
Supplement: Supplementary file 3 [file Table_3.docx]

#### Supplementary Table 3: The mean values for shoot length (cm), root length (cm), seedling fresh weight (g), seedling dry weight (g), salinity tolerance index (STI) and seedling vigor index (STI) of 292 genotypes from the hard winter wheat association mapping panel (HWWAMP) treated with three level of salinity (0 [control], 60 and 120 mM NaCl). *standard deviation.

| Genotype name | salinity level | Shoot length | Std Dev* | Root length | Std Dev | Seedling fresh weight | Std Dev | Seedling dry weight | Std Dev | Salinity Tolerance | Std Dev | SVI | Std Dev |
| --- | --- | --- | --- | --- | --- | --- | --- | --- | --- | --- | --- | --- | --- |
| TRIUMPH64 | 0 | 8 | 0.4 | 6 | 0.3 | 0.15 | 0.01 | 0.06 | 0.01 | 100 | 0 | 14 | 0.5 |
| CHISHOLM | 0 | 8 | 0.5 | 6 | 0.5 | 0.15 | 0.02 | 0.06 | 0.01 | 100 | 0 | 14 | 0.9 |
| CUSTER | 0 | 8 | 0.2 | 6 | 0.3 | 0.15 | 0.01 | 0.06 | 0.01 | 100 | 0 | 14 | 0.6 |
| 2174-05 | 0 | 8 | 0.6 | 7 | 0.6 | 0.16 | 0.01 | 0.07 | 0.01 | 100 | 0 | 15 | 0.2 |
| INTRADA | 0 | 8 | 0.8 | 6 | 0.6 | 0.15 | 0.01 | 0.06 | 0.00 | 100 | 0 | 14 | 0.5 |
| OK101 | 0 | 8 | 0.3 | 6 | 0.3 | 0.16 | 0.01 | 0.05 | 0.00 | 100 | 0 | 14 | 0.4 |
| OK102 | 0 | 8 | 0.3 | 6 | 0.4 | 0.15 | 0.01 | 0.05 | 0.01 | 100 | 0 | 14 | 0.4 |
| ENDURANCE | 0 | 8 | 0.4 | 6 | 0.4 | 0.16 | 0.01 | 0.06 | 0.01 | 100 | 0 | 13 | 0.5 |
| DELIVER | 0 | 8 | 0.7 | 6 | 0.3 | 0.15 | 0.01 | 0.05 | 0.00 | 100 | 0 | 14 | 0.8 |
| OK_BULLET | 0 | 8 | 0.4 | 6 | 0.4 | 0.16 | 0.01 | 0.06 | 0.01 | 100 | 0 | 14 | 0.3 |
| CENTERFIELD | 0 | 8 | 0.5 | 6 | 0.3 | 0.15 | 0.01 | 0.06 | 0.00 | 100 | 0 | 14 | 0.6 |
| GUYMON | 0 | 8 | 0.4 | 7 | 0.5 | 0.16 | 0.01 | 0.06 | 0.01 | 100 | 0 | 15 | 0.1 |
| DUSTER | 0 | 7 | 0.6 | 6 | 0.4 | 0.15 | 0.01 | 0.05 | 0.01 | 100 | 0 | 12 | 0.7 |
| OK_RISING | 0 | 8 | 0.5 | 6 | 0.3 | 0.15 | 0.01 | 0.06 | 0.01 | 100 | 0 | 14 | 0.2 |
| OK02405 | 0 | 8 | 0.4 | 6 | 0.3 | 0.16 | 0.01 | 0.06 | 0.01 | 100 | 0 | 14 | 0.4 |
| PETE | 0 | 8 | 0.5 | 6 | 0.3 | 0.16 | 0.01 | 0.06 | 0.01 | 100 | 0 | 14 | 0.6 |
| BILLINGS | 0 | 7 | 0.3 | 6 | 0.3 | 0.16 | 0.01 | 0.05 | 0.01 | 100 | 0 | 13 | 1.1 |
| OK04505 | 0 | 7 | 0.6 | 6 | 0.5 | 0.16 | 0.01 | 0.06 | 0.01 | 100 | 0 | 14 | 0.7 |
| OK04525 | 0 | 7 | 0.2 | 6 | 0.3 | 0.16 | 0.01 | 0.06 | 0.01 | 100 | 0 | 13 | 0.2 |
| OK04507 | 0 | 8 | 0.6 | 7 | 0.5 | 0.16 | 0.01 | 0.06 | 0.01 | 100 | 0 | 15 | 0.5 |
| OK05830 | 0 | 7 | 0.3 | 6 | 0.3 | 0.15 | 0.01 | 0.06 | 0.01 | 100 | 0 | 13 | 0.6 |
| OK04111 | 0 | 7 | 0.4 | 6 | 0.4 | 0.16 | 0.01 | 0.06 | 0.01 | 100 | 0 | 13 | 0.2 |
| OK04415 | 0 | 8 | 0.4 | 6 | 0.4 | 0.15 | 0.01 | 0.06 | 0.01 | 100 | 0 | 14 | 0.3 |
| OK05711W | 0 | 8 | 0.6 | 6 | 0.6 | 0.16 | 0.01 | 0.06 | 0.01 | 100 | 0 | 14 | 1.1 |
| OK05723W | 0 | 8 | 0.8 | 6 | 0.4 | 0.16 | 0.01 | 0.06 | 0.01 | 100 | 0 | 14 | 0.3 |
| OK05108 | 0 | 8 | 0.6 | 7 | 0.5 | 0.16 | 0.01 | 0.07 | 0.01 | 100 | 0 | 15 | 0.3 |
| OK05122 | 0 | 8 | 0.6 | 6 | 0.5 | 0.16 | 0.01 | 0.06 | 0.01 | 100 | 0 | 14 | 0.5 |
| OK05526 | 0 | 8 | 0.7 | 7 | 0.5 | 0.16 | 0.01 | 0.06 | 0.01 | 100 | 0 | 15 | 0.3 |
| OK05134 | 0 | 7 | 0.5 | 6 | 0.3 | 0.16 | 0.01 | 0.06 | 0.01 | 100 | 0 | 13 | 0.4 |
| OK05303 | 0 | 7 | 0.4 | 6 | 0.3 | 0.15 | 0.01 | 0.05 | 0.01 | 100 | 0 | 13 | 0.3 |
| OK05312 | 0 | 7 | 0.4 | 6 | 0.3 | 0.16 | 0.01 | 0.06 | 0.01 | 100 | 0 | 13 | 0.2 |
| OK05511 | 0 | 7 | 0.4 | 6 | 0.4 | 0.15 | 0.01 | 0.06 | 0.01 | 100 | 0 | 13 | 0.5 |
| OK05204 | 0 | 7 | 0.5 | 6 | 0.4 | 0.16 | 0.01 | 0.06 | 0.01 | 100 | 0 | 13 | 0.5 |
| GARRISON | 0 | 8 | 0.5 | 7 | 0.6 | 0.16 | 0.01 | 0.06 | 0.01 | 100 | 0 | 14 | 1.0 |
| OK06114 | 0 | 8 | 0.5 | 6 | 0.3 | 0.16 | 0.01 | 0.06 | 0.01 | 100 | 0 | 13 | 1.4 |
| OK06210 | 0 | 8 | 0.7 | 7 | 0.5 | 0.16 | 0.01 | 0.06 | 0.01 | 100 | 0 | 15 | 0.7 |
| OK06319 | 0 | 8 | 0.8 | 6 | 0.5 | 0.16 | 0.01 | 0.06 | 0.01 | 100 | 0 | 14 | 1.2 |
| OK06318 | 0 | 8 | 0.5 | 7 | 0.4 | 0.16 | 0.01 | 0.06 | 0.01 | 100 | 0 | 15 | 0.3 |
| OK06336 | 0 | 8 | 0.8 | 6 | 0.5 | 0.16 | 0.01 | 0.06 | 0.01 | 100 | 0 | 14 | 0.9 |
| AGATE | 0 | 8 | 0.5 | 7 | 0.4 | 0.15 | 0.01 | 0.06 | 0.01 | 100 | 0 | 14 | 0.4 |
| ALLIANCE | 0 | 8 | 0.5 | 6 | 0.4 | 0.16 | 0.01 | 0.06 | 0.01 | 100 | 0 | 14 | 0.5 |
| ANTELOPE | 0 | 7 | 0.4 | 6 | 0.3 | 0.16 | 0.01 | 0.06 | 0.01 | 100 | 0 | 13 | 0.3 |
| ARAPAHOE | 0 | 7 | 0.4 | 6 | 0.2 | 0.16 | 0.01 | 0.06 | 0.01 | 100 | 0 | 13 | 0.4 |
| BENNETT | 0 | 8 | 0.4 | 7 | 0.5 | 0.16 | 0.01 | 0.06 | 0.01 | 100 | 0 | 14 | 0.7 |
| BUCKSKIN | 0 | 8 | 0.5 | 6 | 0.4 | 0.16 | 0.01 | 0.06 | 0.01 | 100 | 0 | 14 | 0.5 |
| CENTURK78 | 0 | 8 | 0.5 | 6 | 0.3 | 0.16 | 0.01 | 0.06 | 0.01 | 100 | 0 | 14 | 0.3 |
| CHEYENNE | 0 | 7 | 0.5 | 6 | 0.5 | 0.15 | 0.01 | 0.06 | 0.01 | 100 | 0 | 13 | 0.8 |
| COLT | 0 | 8 | 0.7 | 7 | 0.4 | 0.15 | 0.01 | 0.06 | 0.01 | 100 | 0 | 14 | 0.3 |
| COUGAR | 0 | 8 | 0.7 | 7 | 0.4 | 0.16 | 0.01 | 0.06 | 0.01 | 100 | 0 | 14 | 0.5 |
| CULVER | 0 | 8 | 0.5 | 7 | 0.4 | 0.16 | 0.01 | 0.06 | 0.01 | 100 | 0 | 15 | 0.3 |
| GAGE | 0 | 8 | 0.5 | 7 | 0.5 | 0.16 | 0.01 | 0.05 | 0.01 | 100 | 0 | 15 | 0.4 |
| GOODSTREAK | 0 | 8 | 0.6 | 7 | 0.4 | 0.16 | 0.01 | 0.06 | 0.01 | 100 | 0 | 14 | 0.4 |
| HALLAM | 0 | 7 | 0.5 | 7 | 0.4 | 0.16 | 0.01 | 0.06 | 0.01 | 100 | 0 | 14 | 1.0 |
| HARRY | 0 | 7 | 0.6 | 6 | 0.4 | 0.15 | 0.01 | 0.06 | 0.00 | 100 | 0 | 13 | 0.3 |
| HOMESTEAD | 0 | 8 | 0.6 | 6 | 0.5 | 0.16 | 0.01 | 0.06 | 0.01 | 100 | 0 | 14 | 0.5 |
| INFINITY_CL | 0 | 8 | 0.5 | 6 | 0.3 | 0.15 | 0.01 | 0.06 | 0.01 | 100 | 0 | 14 | 0.2 |
| KHARKOF | 0 | 8 | 0.7 | 6 | 0.5 | 0.16 | 0.01 | 0.06 | 0.01 | 100 | 0 | 14 | 0.3 |
| MILLENNIUM | 0 | 7 | 0.9 | 7 | 0.7 | 0.16 | 0.01 | 0.06 | 0.01 | 100 | 0 | 14 | 1.2 |
| CAMELOT | 0 | 7 | 0.5 | 6 | 0.3 | 0.16 | 0.01 | 0.06 | 0.00 | 100 | 0 | 13 | 0.6 |
| OVERLAND | 0 | 7 | 0.6 | 7 | 0.7 | 0.15 | 0.01 | 0.06 | 0.01 | 100 | 0 | 14 | 0.3 |
| NE99495 | 0 | 8 | 0.3 | 7 | 0.5 | 0.16 | 0.01 | 0.06 | 0.01 | 100 | 0 | 15 | 0.4 |
| NIOBRARA | 0 | 8 | 0.5 | 6 | 0.3 | 0.16 | 0.01 | 0.06 | 0.01 | 100 | 0 | 14 | 0.4 |
| NUPLAINS | 0 | 8 | 0.3 | 6 | 0.2 | 0.16 | 0.00 | 0.06 | 0.01 | 100 | 0 | 14 | 0.5 |
| PRONGHORN | 0 | 7 | 0.5 | 6 | 0.3 | 0.16 | 0.01 | 0.06 | 0.01 | 100 | 0 | 13 | 0.5 |
| RAWHIDE | 0 | 7 | 0.4 | 6 | 0.6 | 0.15 | 0.01 | 0.06 | 0.01 | 100 | 0 | 14 | 0.3 |
| REDLAND | 0 | 7 | 0.4 | 6 | 0.3 | 0.16 | 0.01 | 0.06 | 0.01 | 100 | 0 | 13 | 0.6 |
| SCOUT66 | 0 | 8 | 0.7 | 7 | 0.6 | 0.16 | 0.01 | 0.06 | 0.01 | 100 | 0 | 14 | 0.8 |
| SIOUXLAND | 0 | 7 | 0.4 | 6 | 0.4 | 0.16 | 0.01 | 0.06 | 0.01 | 100 | 0 | 13 | 0.6 |
| TURKEY_NEBSEL | 0 | 7 | 0.4 | 6 | 0.2 | 0.16 | 0.00 | 0.06 | 0.01 | 100 | 0 | 13 | 0.7 |
| VISTA | 0 | 8 | 0.5 | 6 | 0.2 | 0.16 | 0.01 | 0.06 | 0.01 | 100 | 0 | 13 | 0.5 |
| WAHOO | 0 | 8 | 0.8 | 7 | 0.6 | 0.16 | 0.01 | 0.06 | 0.01 | 100 | 0 | 15 | 0.5 |
| WARRIOR | 0 | 7 | 0.6 | 6 | 0.5 | 0.16 | 0.01 | 0.06 | 0.00 | 100 | 0 | 14 | 0.5 |
| WESLEY | 0 | 8 | 0.4 | 6 | 0.4 | 0.16 | 0.01 | 0.06 | 0.01 | 100 | 0 | 14 | 0.0 |
| WICHITA | 0 | 8 | 0.5 | 7 | 0.2 | 0.16 | 0.01 | 0.06 | 0.01 | 100 | 0 | 14 | 0.1 |
| WINDSTAR | 0 | 7 | 0.5 | 6 | 0.4 | 0.16 | 0.01 | 0.06 | 0.01 | 100 | 0 | 13 | 0.7 |
| LANCER | 0 | 8 | 0.5 | 7 | 0.3 | 0.16 | 0.01 | 0.06 | 0.01 | 100 | 0 | 15 | 0.2 |
| ANTON | 0 | 7 | 0.3 | 7 | 0.4 | 0.16 | 0.01 | 0.06 | 0.01 | 100 | 0 | 14 | 0.8 |
| MACE | 0 | 7 | 0.3 | 6 | 0.4 | 0.16 | 0.01 | 0.06 | 0.01 | 100 | 0 | 13 | 0.3 |
| TAM107-R7 | 0 | 7 | 0.4 | 6 | 0.5 | 0.16 | 0.01 | 0.06 | 0.01 | 100 | 0 | 13 | 0.5 |
| ARLIN | 0 | 8 | 0.5 | 6 | 0.5 | 0.16 | 0.01 | 0.06 | 0.01 | 100 | 0 | 13 | 0.9 |
| ALICE | 0 | 8 | 0.4 | 7 | 0.5 | 0.15 | 0.01 | 0.06 | 0.01 | 100 | 0 | 15 | 0.5 |
| DARRELL | 0 | 8 | 0.5 | 6 | 0.3 | 0.16 | 0.01 | 0.06 | 0.01 | 100 | 0 | 14 | 0.4 |
| EXPEDITION | 0 | 8 | 0.5 | 6 | 0.4 | 0.16 | 0.01 | 0.06 | 0.01 | 100 | 0 | 13 | 0.2 |
| WENDY | 0 | 8 | 0.5 | 7 | 0.3 | 0.16 | 0.01 | 0.06 | 0.01 | 100 | 0 | 15 | 0.4 |
| SD00111-9 | 0 | 8 | 0.5 | 7 | 0.4 | 0.16 | 0.01 | 0.06 | 0.01 | 100 | 0 | 14 | 0.7 |
| SD01237 | 0 | 7 | 0.4 | 7 | 0.4 | 0.15 | 0.01 | 0.06 | 0.01 | 100 | 0 | 14 | 0.3 |
| SD01058 | 0 | 7 | 0.5 | 6 | 0.4 | 0.16 | 0.01 | 0.06 | 0.01 | 100 | 0 | 13 | 0.6 |
| SD05118 | 0 | 8 | 0.5 | 7 | 0.4 | 0.16 | 0.01 | 0.06 | 0.01 | 100 | 0 | 15 | 0.5 |
| SD05210 | 0 | 8 | 0.7 | 6 | 0.4 | 0.16 | 0.01 | 0.06 | 0.01 | 100 | 0 | 13 | 0.5 |
| SD05W018 | 0 | 7 | 0.4 | 6 | 0.5 | 0.16 | 0.01 | 0.06 | 0.01 | 100 | 0 | 13 | 0.2 |
| NEKOTA | 0 | 8 | 0.6 | 7 | 0.5 | 0.16 | 0.01 | 0.06 | 0.01 | 100 | 0 | 15 | 0.8 |
| TANDEM | 0 | 8 | 0.3 | 7 | 0.5 | 0.16 | 0.01 | 0.06 | 0.01 | 100 | 0 | 14 | 1.0 |
| CRIMSON | 0 | 7 | 0.3 | 6 | 0.5 | 0.16 | 0.01 | 0.06 | 0.01 | 100 | 0 | 14 | 0.2 |
| ROSE | 0 | 7 | 0.4 | 6 | 0.5 | 0.16 | 0.01 | 0.06 | 0.01 | 100 | 0 | 13 | 0.5 |
| DAWN | 0 | 8 | 0.4 | 7 | 0.5 | 0.16 | 0.01 | 0.06 | 0.01 | 100 | 0 | 14 | 0.8 |
| WINOKA | 0 | 7 | 0.5 | 6 | 0.2 | 0.15 | 0.01 | 0.06 | 0.01 | 100 | 0 | 12 | 0.2 |
| NELL | 0 | 8 | 0.6 | 7 | 0.5 | 0.16 | 0.01 | 0.06 | 0.01 | 100 | 0 | 15 | 0.4 |
| RITA | 0 | 7 | 0.4 | 6 | 0.3 | 0.15 | 0.01 | 0.06 | 0.01 | 100 | 0 | 13 | 0.2 |
| BRONZE | 0 | 7 | 0.3 | 6 | 0.4 | 0.16 | 0.01 | 0.06 | 0.01 | 100 | 0 | 13 | 0.4 |
| HUME | 0 | 8 | 0.6 | 7 | 0.5 | 0.16 | 0.01 | 0.06 | 0.01 | 100 | 0 | 14 | 0.6 |
| GENT | 0 | 7 | 0.5 | 6 | 0.3 | 0.15 | 0.01 | 0.06 | 0.01 | 100 | 0 | 13 | 0.6 |
| HARDING | 0 | 7 | 0.4 | 6 | 0.3 | 0.15 | 0.01 | 0.06 | 0.01 | 100 | 0 | 13 | 0.2 |
| HV9W03-1551WP | 0 | 8 | 0.5 | 7 | 0.5 | 0.16 | 0.01 | 0.06 | 0.01 | 100 | 0 | 14 | 0.6 |
| G1878 | 0 | 8 | 0.6 | 7 | 0.4 | 0.17 | 0.01 | 0.06 | 0.01 | 100 | 0 | 15 | 0.3 |
| HV9W03-1379R | 0 | 8 | 0.5 | 7 | 0.5 | 0.16 | 0.01 | 0.06 | 0.01 | 100 | 0 | 14 | 0.1 |
| HV9W03-1596R | 0 | 7 | 0.4 | 6 | 0.3 | 0.16 | 0.01 | 0.06 | 0.01 | 100 | 0 | 13 | 0.5 |
| HV9W05-1280R | 0 | 7 | 0.4 | 6 | 0.2 | 0.15 | 0.01 | 0.05 | 0.01 | 100 | 0 | 12 | 0.5 |
| HV9W06-504 | 0 | 7 | 0.2 | 6 | 0.4 | 0.16 | 0.01 | 0.06 | 0.01 | 100 | 0 | 13 | 0.5 |
| SPARTAN | 0 | 7 | 0.6 | 7 | 0.4 | 0.16 | 0.01 | 0.06 | 0.01 | 100 | 0 | 14 | 0.3 |
| HV906-865 | 0 | 8 | 0.9 | 7 | 0.8 | 0.16 | 0.01 | 0.07 | 0.01 | 100 | 0 | 15 | 0.5 |
| TARKIO | 0 | 7 | 0.3 | 6 | 0.4 | 0.16 | 0.01 | 0.06 | 0.01 | 100 | 0 | 13 | 0.5 |
| SMOKYHILL | 0 | 7 | 0.5 | 6 | 0.6 | 0.16 | 0.01 | 0.06 | 0.01 | 100 | 0 | 13 | 0.6 |
| SHOCKER | 0 | 7 | 0.5 | 7 | 0.7 | 0.16 | 0.01 | 0.06 | 0.01 | 100 | 0 | 13 | 0.6 |
| VONA | 0 | 7 | 0.4 | 6 | 0.4 | 0.16 | 0.01 | 0.06 | 0.01 | 100 | 0 | 13 | 0.3 |
| CO940610 | 0 | 7 | 0.5 | 6 | 0.5 | 0.16 | 0.01 | 0.06 | 0.01 | 100 | 0 | 14 | 0.3 |
| AVALANCHE | 0 | 8 | 0.5 | 7 | 0.4 | 0.16 | 0.01 | 0.06 | 0.01 | 100 | 0 | 15 | 0.4 |
| BOND_CL | 0 | 7 | 0.5 | 6 | 0.5 | 0.16 | 0.01 | 0.06 | 0.01 | 100 | 0 | 13 | 0.4 |
| PLATTE | 0 | 7 | 0.4 | 6 | 0.3 | 0.16 | 0.01 | 0.05 | 0.01 | 100 | 0 | 13 | 0.4 |
| LINDON | 0 | 7 | 0.5 | 7 | 0.5 | 0.16 | 0.01 | 0.06 | 0.01 | 100 | 0 | 14 | 0.7 |
| CO03W043 | 0 | 7 | 0.6 | 6 | 0.5 | 0.16 | 0.01 | 0.06 | 0.01 | 100 | 0 | 14 | 0.5 |
| SNOWMASS | 0 | 7 | 0.6 | 6 | 0.5 | 0.16 | 0.01 | 0.06 | 0.01 | 100 | 0 | 13 | 1.1 |
| THUNDER_CL | 0 | 8 | 0.5 | 7 | 0.6 | 0.16 | 0.01 | 0.06 | 0.01 | 100 | 0 | 14 | 0.7 |
| CO04025 | 0 | 7 | 0.3 | 7 | 0.5 | 0.16 | 0.01 | 0.06 | 0.01 | 100 | 0 | 13 | 0.2 |
| CO04393 | 0 | 8 | 0.5 | 7 | 0.5 | 0.16 | 0.01 | 0.06 | 0.01 | 100 | 0 | 14 | 1.1 |
| CO04499 | 0 | 7 | 0.5 | 6 | 0.5 | 0.16 | 0.01 | 0.06 | 0.01 | 100 | 0 | 14 | 0.3 |
| CO04W320 | 0 | 9 | 0.9 | 7 | 0.5 | 0.16 | 0.01 | 0.07 | 0.01 | 100 | 0 | 16 | 0.5 |
| LAMAR | 0 | 8 | 0.6 | 7 | 0.5 | 0.15 | 0.01 | 0.06 | 0.01 | 100 | 0 | 14 | 0.5 |
| CARSON | 0 | 9 | 0.9 | 8 | 0.7 | 0.17 | 0.01 | 0.07 | 0.01 | 100 | 0 | 16 | 0.5 |
| HAIL | 0 | 8 | 0.7 | 7 | 0.5 | 0.16 | 0.01 | 0.06 | 0.01 | 100 | 0 | 15 | 0.4 |
| SANDY | 0 | 7 | 0.3 | 6 | 0.5 | 0.16 | 0.01 | 0.06 | 0.01 | 100 | 0 | 14 | 0.4 |
| DUKE | 0 | 7 | 0.5 | 7 | 0.5 | 0.16 | 0.01 | 0.06 | 0.01 | 100 | 0 | 13 | 0.9 |
| HALT | 0 | 8 | 0.7 | 7 | 0.5 | 0.16 | 0.01 | 0.06 | 0.01 | 100 | 0 | 15 | 0.6 |
| HATCHER | 0 | 7 | 0.3 | 6 | 0.2 | 0.15 | 0.01 | 0.06 | 0.01 | 100 | 0 | 13 | 0.1 |
| PRAIRIE_RED | 0 | 8 | 0.5 | 7 | 0.5 | 0.16 | 0.01 | 0.06 | 0.01 | 100 | 0 | 14 | 1.0 |
| ABOVE | 0 | 7 | 0.4 | 6 | 0.5 | 0.16 | 0.01 | 0.06 | 0.00 | 100 | 0 | 13 | 0.3 |
| CO03064 | 0 | 7 | 0.3 | 6 | 0.5 | 0.15 | 0.01 | 0.06 | 0.01 | 100 | 0 | 13 | 0.6 |
| BILL_BROWN | 0 | 8 | 0.6 | 7 | 0.4 | 0.16 | 0.01 | 0.06 | 0.01 | 100 | 0 | 14 | 0.4 |
| RIPPER | 0 | 7 | 0.5 | 7 | 0.6 | 0.16 | 0.01 | 0.06 | 0.01 | 100 | 0 | 14 | 1.1 |
| PROWERS | 0 | 8 | 0.8 | 7 | 0.6 | 0.16 | 0.01 | 0.06 | 0.01 | 100 | 0 | 14 | 0.8 |
| AKRON | 0 | 8 | 0.5 | 7 | 0.6 | 0.16 | 0.01 | 0.06 | 0.01 | 100 | 0 | 14 | 0.3 |
| JULES | 0 | 8 | 0.5 | 7 | 0.5 | 0.16 | 0.01 | 0.06 | 0.01 | 100 | 0 | 15 | 0.6 |
| YUMA | 0 | 8 | 0.6 | 7 | 0.4 | 0.16 | 0.01 | 0.06 | 0.01 | 100 | 0 | 15 | 0.7 |
| TAMW-101 | 0 | 8 | 0.6 | 7 | 0.4 | 0.16 | 0.01 | 0.06 | 0.01 | 100 | 0 | 15 | 1.2 |
| TAM105 | 0 | 8 | 0.6 | 7 | 0.4 | 0.16 | 0.01 | 0.06 | 0.01 | 100 | 0 | 15 | 0.6 |
| TAM107 | 0 | 7 | 0.3 | 6 | 0.5 | 0.16 | 0.01 | 0.05 | 0.01 | 100 | 0 | 13 | 0.3 |
| TAM109 | 0 | 7 | 0.4 | 6 | 0.2 | 0.15 | 0.01 | 0.05 | 0.01 | 100 | 0 | 13 | 0.1 |
| TAM110 | 0 | 8 | 0.5 | 7 | 0.6 | 0.16 | 0.01 | 0.05 | 0.00 | 100 | 0 | 14 | 0.9 |
| TAM111 | 0 | 8 | 0.6 | 7 | 0.6 | 0.16 | 0.01 | 0.05 | 0.00 | 100 | 0 | 14 | 1.0 |
| TAM112 | 0 | 8 | 0.4 | 7 | 0.4 | 0.16 | 0.01 | 0.06 | 0.01 | 100 | 0 | 15 | 0.6 |
| TAM200 | 0 | 7 | 0.5 | 6 | 0.5 | 0.16 | 0.00 | 0.05 | 0.01 | 100 | 0 | 13 | 0.4 |
| TAM202 | 0 | 7 | 0.5 | 7 | 0.4 | 0.16 | 0.01 | 0.06 | 0.01 | 100 | 0 | 14 | 1.0 |
| TAM203 | 0 | 7 | 0.4 | 7 | 0.6 | 0.16 | 0.00 | 0.05 | 0.00 | 100 | 0 | 13 | 0.9 |
| TAM302 | 0 | 8 | 0.7 | 7 | 0.3 | 0.16 | 0.01 | 0.06 | 0.01 | 100 | 0 | 15 | 0.5 |
| TAM303 | 0 | 8 | 0.4 | 7 | 0.8 | 0.15 | 0.01 | 0.05 | 0.01 | 100 | 0 | 14 | 0.8 |
| TAM304 | 0 | 7 | 0.3 | 6 | 0.6 | 0.16 | 0.01 | 0.05 | 0.00 | 100 | 0 | 14 | 0.7 |
| TAM400 | 0 | 8 | 0.6 | 7 | 0.4 | 0.16 | 0.01 | 0.05 | 0.00 | 100 | 0 | 14 | 0.6 |
| LOCKETT | 0 | 8 | 0.5 | 7 | 0.7 | 0.16 | 0.01 | 0.06 | 0.01 | 100 | 0 | 14 | 0.6 |
| STURDY | 0 | 7 | 0.5 | 7 | 0.4 | 0.16 | 0.01 | 0.05 | 0.00 | 100 | 0 | 13 | 0.6 |
| STURDY_2K | 0 | 7 | 0.3 | 6 | 0.3 | 0.15 | 0.01 | 0.06 | 0.01 | 100 | 0 | 13 | 0.4 |
| MIT | 0 | 7 | 0.3 | 6 | 0.4 | 0.16 | 0.01 | 0.05 | 0.00 | 100 | 0 | 13 | 0.3 |
| CAPROCK | 0 | 8 | 0.5 | 7 | 0.7 | 0.16 | 0.01 | 0.05 | 0.00 | 100 | 0 | 14 | 0.8 |
| TX01A5936 | 0 | 7 | 0.5 | 6 | 0.4 | 0.16 | 0.01 | 0.06 | 0.01 | 100 | 0 | 13 | 0.5 |
| TAM401 | 0 | 8 | 0.7 | 7 | 0.6 | 0.16 | 0.01 | 0.05 | 0.00 | 100 | 0 | 15 | 0.7 |
| TX02A0252 | 0 | 6 | 0.5 | 6 | 0.2 | 0.16 | 0.01 | 0.05 | 0.00 | 100 | 0 | 12 | 0.7 |
| TX03A0148 | 0 | 7 | 0.5 | 6 | 0.3 | 0.15 | 0.01 | 0.05 | 0.01 | 100 | 0 | 13 | 0.3 |
| TX03A0563 | 0 | 7 | 0.5 | 6 | 0.2 | 0.15 | 0.01 | 0.06 | 0.01 | 100 | 0 | 13 | 0.6 |
| TX04A001246 | 0 | 8 | 0.6 | 7 | 0.6 | 0.17 | 0.01 | 0.06 | 0.01 | 100 | 0 | 13 | 0.6 |
| TX01V5134RC-3 | 0 | 8 | 0.5 | 7 | 0.3 | 0.16 | 0.01 | 0.05 | 0.00 | 100 | 0 | 15 | 0.2 |
| TX04M410164 | 0 | 6 | 0.6 | 6 | 0.3 | 0.15 | 0.01 | 0.06 | 0.01 | 100 | 0 | 12 | 0.2 |
| TX04M410211 | 0 | 9 | 0.8 | 8 | 0.5 | 0.17 | 0.01 | 0.07 | 0.01 | 100 | 0 | 16 | 0.4 |
| TX04V075080 | 0 | 8 | 0.3 | 7 | 0.3 | 0.16 | 0.01 | 0.05 | 0.01 | 100 | 0 | 15 | 0.6 |
| TX99A0153-1 | 0 | 7 | 0.5 | 6 | 0.2 | 0.15 | 0.01 | 0.06 | 0.00 | 100 | 0 | 13 | 0.4 |
| TX01M5009-28 | 0 | 8 | 0.5 | 7 | 0.5 | 0.16 | 0.01 | 0.05 | 0.00 | 100 | 0 | 15 | 0.5 |
| TX00V1131 | 0 | 7 | 0.5 | 7 | 0.5 | 0.15 | 0.01 | 0.06 | 0.01 | 100 | 0 | 13 | 0.9 |
| TX99U8618 | 0 | 7 | 0.4 | 7 | 0.6 | 0.16 | 0.01 | 0.06 | 0.01 | 100 | 0 | 14 | 0.6 |
| TX96D1073 | 0 | 7 | 0.6 | 7 | 0.6 | 0.16 | 0.01 | 0.05 | 0.00 | 100 | 0 | 14 | 0.8 |
| 2180 | 0 | 7 | 0.5 | 6 | 0.3 | 0.16 | 0.01 | 0.05 | 0.01 | 100 | 0 | 13 | 1.2 |
| HG-9 | 0 | 7 | 0.5 | 6 | 0.3 | 0.15 | 0.01 | 0.05 | 0.01 | 100 | 0 | 12 | 0.7 |
| TX86A5606 | 0 | 9 | 0.7 | 8 | 0.5 | 0.17 | 0.01 | 0.07 | 0.01 | 100 | 0 | 16 | 0.4 |
| TX86A8072 | 0 | 8 | 0.7 | 7 | 0.6 | 0.16 | 0.01 | 0.06 | 0.01 | 100 | 0 | 15 | 0.4 |
| CREST | 0 | 7 | 0.3 | 6 | 0.4 | 0.16 | 0.01 | 0.05 | 0.00 | 100 | 0 | 13 | 0.5 |
| ROSEBUD | 0 | 8 | 0.7 | 7 | 0.6 | 0.16 | 0.01 | 0.05 | 0.01 | 100 | 0 | 15 | 0.2 |
| JUDITH | 0 | 7 | 0.5 | 6 | 0.4 | 0.15 | 0.01 | 0.05 | 0.01 | 100 | 0 | 13 | 0.7 |
| MT85200 | 0 | 8 | 0.4 | 7 | 0.4 | 0.16 | 0.01 | 0.06 | 0.01 | 100 | 0 | 15 | 0.2 |
| NUSKY | 0 | 8 | 0.6 | 7 | 0.5 | 0.16 | 0.01 | 0.06 | 0.01 | 100 | 0 | 15 | 1.2 |
| MT9513 | 0 | 8 | 0.5 | 7 | 0.5 | 0.16 | 0.01 | 0.06 | 0.01 | 100 | 0 | 14 | 0.4 |
| MT9904 | 0 | 8 | 0.3 | 7 | 0.5 | 0.16 | 0.01 | 0.06 | 0.01 | 100 | 0 | 14 | 0.9 |
| NORRIS | 0 | 8 | 0.4 | 7 | 0.6 | 0.16 | 0.01 | 0.06 | 0.01 | 100 | 0 | 15 | 0.5 |
| YELLOWSTONE | 0 | 8 | 0.4 | 7 | 0.6 | 0.16 | 0.01 | 0.06 | 0.01 | 100 | 0 | 15 | 0.4 |
| MT0495 | 0 | 7 | 0.7 | 7 | 0.7 | 0.16 | 0.01 | 0.05 | 0.01 | 100 | 0 | 14 | 1.0 |
| MTS0531 | 0 | 8 | 0.4 | 7 | 0.4 | 0.16 | 0.01 | 0.05 | 0.01 | 100 | 0 | 15 | 0.3 |
| DECADE | 0 | 8 | 0.5 | 6 | 0.3 | 0.15 | 0.01 | 0.06 | 0.01 | 100 | 0 | 14 | 0.7 |
| MT06103 | 0 | 8 | 0.7 | 7 | 0.5 | 0.16 | 0.01 | 0.06 | 0.01 | 100 | 0 | 14 | 1.0 |
| JUDEE | 0 | 8 | 0.5 | 6 | 0.3 | 0.15 | 0.01 | 0.06 | 0.01 | 100 | 0 | 14 | 0.4 |
| LAKIN | 0 | 7 | 0.7 | 6 | 0.5 | 0.15 | 0.01 | 0.06 | 0.01 | 100 | 0 | 14 | 0.8 |
| STANTON | 0 | 7 | 0.6 | 7 | 0.4 | 0.15 | 0.01 | 0.06 | 0.01 | 100 | 0 | 14 | 0.6 |
| TREGO | 0 | 8 | 0.5 | 7 | 0.6 | 0.16 | 0.01 | 0.05 | 0.01 | 100 | 0 | 15 | 0.4 |
| KARL_92 | 0 | 7 | 0.5 | 6 | 0.4 | 0.15 | 0.01 | 0.05 | 0.01 | 100 | 0 | 13 | 0.5 |
| DODGE | 0 | 8 | 0.6 | 7 | 0.5 | 0.15 | 0.01 | 0.06 | 0.01 | 100 | 0 | 14 | 1.1 |
| NORKAN | 0 | 7 | 0.6 | 6 | 0.5 | 0.15 | 0.01 | 0.06 | 0.01 | 100 | 0 | 14 | 0.4 |
| CHENEY | 0 | 7 | 0.6 | 6 | 0.4 | 0.15 | 0.01 | 0.05 | 0.01 | 100 | 0 | 13 | 0.6 |
| NEWTON | 0 | 8 | 0.8 | 7 | 0.6 | 0.15 | 0.01 | 0.06 | 0.01 | 100 | 0 | 15 | 0.6 |
| LARNED | 0 | 8 | 0.5 | 6 | 0.5 | 0.16 | 0.00 | 0.06 | 0.01 | 100 | 0 | 14 | 0.7 |
| PARKER76 | 0 | 7 | 0.7 | 6 | 0.3 | 0.15 | 0.01 | 0.05 | 0.01 | 100 | 0 | 13 | 0.9 |
| KIRWIN | 0 | 8 | 1.0 | 7 | 0.6 | 0.15 | 0.01 | 0.05 | 0.01 | 100 | 0 | 14 | 1.2 |
| SAGE | 0 | 8 | 0.6 | 6 | 0.5 | 0.15 | 0.01 | 0.06 | 0.01 | 100 | 0 | 14 | 1.1 |
| TRISON | 0 | 7 | 0.8 | 7 | 0.4 | 0.15 | 0.01 | 0.05 | 0.01 | 100 | 0 | 14 | 0.7 |
| EAGLE | 0 | 7 | 0.4 | 6 | 0.2 | 0.15 | 0.01 | 0.05 | 0.01 | 100 | 0 | 13 | 0.4 |
| SHAWNEE | 0 | 8 | 0.4 | 7 | 0.4 | 0.14 | 0.02 | 0.05 | 0.01 | 100 | 0 | 14 | 0.5 |
| PARKER | 0 | 8 | 0.5 | 7 | 0.5 | 0.15 | 0.01 | 0.06 | 0.01 | 100 | 0 | 14 | 0.4 |
| KAW61 | 0 | 8 | 0.4 | 7 | 0.5 | 0.15 | 0.01 | 0.06 | 0.00 | 100 | 0 | 14 | 1.1 |
| TASCOSA | 0 | 8 | 0.4 | 7 | 0.3 | 0.16 | 0.01 | 0.06 | 0.01 | 100 | 0 | 15 | 0.5 |
| BISON | 0 | 8 | 0.4 | 7 | 0.5 | 0.15 | 0.01 | 0.06 | 0.01 | 100 | 0 | 14 | 0.5 |
| KIOWA | 0 | 8 | 0.5 | 6 | 0.4 | 0.15 | 0.01 | 0.06 | 0.01 | 100 | 0 | 14 | 0.6 |
| WICHITA | 0 | 7 | 0.5 | 6 | 0.2 | 0.15 | 0.01 | 0.06 | 0.00 | 100 | 0 | 13 | 0.6 |
| COMANCHE | 0 | 8 | 0.6 | 7 | 0.5 | 0.16 | 0.01 | 0.06 | 0.01 | 100 | 0 | 15 | 0.7 |
| BAKERS_WHITE | 0 | 8 | 0.6 | 7 | 0.4 | 0.15 | 0.00 | 0.06 | 0.01 | 100 | 0 | 13 | 1.4 |
| BURCHETT | 0 | 7 | 0.7 | 6 | 0.2 | 0.15 | 0.01 | 0.06 | 0.01 | 100 | 0 | 13 | 0.4 |
| CUTTER | 0 | 8 | 0.5 | 7 | 0.5 | 0.16 | 0.01 | 0.06 | 0.01 | 100 | 0 | 14 | 0.5 |
| DUMAS | 0 | 7 | 0.7 | 6 | 0.3 | 0.15 | 0.01 | 0.05 | 0.00 | 100 | 0 | 13 | 0.3 |
| HONDO | 0 | 7 | 0.6 | 6 | 0.2 | 0.15 | 0.01 | 0.06 | 0.01 | 100 | 0 | 13 | 0.6 |
| JAGALENE | 0 | 7 | 0.5 | 6 | 0.3 | 0.15 | 0.01 | 0.05 | 0.01 | 100 | 0 | 13 | 0.6 |
| LONGHORN | 0 | 7 | 0.4 | 6 | 0.2 | 0.15 | 0.01 | 0.05 | 0.00 | 100 | 0 | 13 | 0.3 |
| NEOSHO | 0 | 8 | 0.5 | 7 | 0.5 | 0.16 | 0.01 | 0.06 | 0.01 | 100 | 0 | 14 | 0.6 |
| OGALLALA | 0 | 7 | 0.6 | 6 | 0.3 | 0.15 | 0.01 | 0.05 | 0.00 | 100 | 0 | 13 | 0.7 |
| POSTROCK | 0 | 7 | 0.4 | 6 | 0.2 | 0.15 | 0.01 | 0.06 | 0.01 | 100 | 0 | 13 | 0.9 |
| THUNDERBOLT | 0 | 8 | 0.3 | 7 | 0.3 | 0.16 | 0.01 | 0.06 | 0.01 | 100 | 0 | 15 | 0.3 |
| W04-417 | 0 | 7 | 0.5 | 6 | 0.4 | 0.15 | 0.01 | 0.05 | 0.01 | 100 | 0 | 13 | 0.6 |
| NUFRONTIER | 0 | 8 | 0.5 | 7 | 0.4 | 0.15 | 0.01 | 0.06 | 0.01 | 100 | 0 | 14 | 0.3 |
| NUHORIZON | 0 | 7 | 0.5 | 6 | 0.4 | 0.15 | 0.00 | 0.05 | 0.01 | 100 | 0 | 13 | 0.5 |
| ONAGA | 0 | 9 | 0.9 | 8 | 0.7 | 0.16 | 0.01 | 0.06 | 0.01 | 100 | 0 | 17 | 0.9 |
| RONL | 0 | 7 | 0.5 | 6 | 0.6 | 0.15 | 0.01 | 0.06 | 0.00 | 100 | 0 | 14 | 0.4 |
| 2145 | 0 | 8 | 0.5 | 6 | 0.4 | 0.15 | 0.01 | 0.06 | 0.01 | 100 | 0 | 14 | 0.5 |
| HEYNE | 0 | 7 | 0.5 | 6 | 0.5 | 0.15 | 0.01 | 0.06 | 0.00 | 100 | 0 | 13 | 0.4 |
| KS00F5-20-3 | 0 | 7 | 0.4 | 6 | 0.5 | 0.15 | 0.01 | 0.06 | 0.01 | 100 | 0 | 13 | 0.7 |
| OVERLEY | 0 | 7 | 0.6 | 7 | 0.5 | 0.15 | 0.01 | 0.06 | 0.01 | 100 | 0 | 14 | 0.7 |
| FULLER | 0 | 8 | 0.1 | 7 | 0.4 | 0.16 | 0.01 | 0.06 | 0.01 | 100 | 0 | 15 | 0.4 |
| COSSACK | 0 | 8 | 0.3 | 7 | 0.5 | 0.16 | 0.01 | 0.06 | 0.01 | 100 | 0 | 15 | 0.8 |
| ENHANCER | 0 | 8 | 0.0 | 7 | 0.3 | 0.16 | 0.01 | 0.06 | 0.01 | 100 | 0 | 15 | 0.3 |
| SANTA_FE | 0 | 8 | 0.0 | 7 | 0.3 | 0.15 | 0.01 | 0.05 | 0.01 | 100 | 0 | 15 | 0.3 |
| VENANGO | 0 | 8 | 0.4 | 7 | 0.4 | 0.15 | 0.01 | 0.05 | 0.01 | 100 | 0 | 14 | 0.5 |
| WB411W | 0 | 8 | 0.4 | 7 | 0.4 | 0.16 | 0.01 | 0.06 | 0.01 | 100 | 0 | 15 | 0.2 |
| KEOTA | 0 | 8 | 0.5 | 7 | 0.5 | 0.16 | 0.01 | 0.06 | 0.01 | 100 | 0 | 14 | 0.5 |
| TX05A001822 | 0 | 7 | 0.5 | 7 | 0.5 | 0.15 | 0.01 | 0.06 | 0.00 | 100 | 0 | 13 | 0.7 |
| TX06A001263 | 0 | 7 | 0.5 | 6 | 0.4 | 0.15 | 0.01 | 0.06 | 0.01 | 100 | 0 | 13 | 0.7 |
| TX06A001132 | 0 | 7 | 0.6 | 7 | 0.5 | 0.15 | 0.01 | 0.06 | 0.01 | 100 | 0 | 14 | 0.9 |
| TX06A001281 | 0 | 8 | 0.4 | 6 | 0.4 | 0.15 | 0.01 | 0.05 | 0.01 | 100 | 0 | 14 | 0.9 |
| TX06A001386 | 0 | 7 | 0.4 | 6 | 0.4 | 0.15 | 0.01 | 0.06 | 0.00 | 100 | 0 | 13 | 0.5 |
| TX05V7259 | 0 | 8 | 0.4 | 7 | 0.4 | 0.15 | 0.01 | 0.06 | 0.01 | 100 | 0 | 14 | 0.5 |
| TX05V7269 | 0 | 8 | 0.4 | 7 | 0.5 | 0.15 | 0.01 | 0.06 | 0.01 | 100 | 0 | 14 | 0.6 |
| TX05A001188 | 0 | 8 | 0.3 | 6 | 0.5 | 0.16 | 0.01 | 0.06 | 0.01 | 100 | 0 | 14 | 0.4 |
| TX07A001279 | 0 | 7 | 0.4 | 7 | 0.5 | 0.16 | 0.01 | 0.06 | 0.01 | 100 | 0 | 14 | 0.5 |
| TX07A001318 | 0 | 8 | 0.5 | 7 | 0.6 | 0.16 | 0.01 | 0.06 | 0.01 | 100 | 0 | 14 | 0.4 |
| TX07A001420 | 0 | 8 | 0.5 | 7 | 0.5 | 0.16 | 0.01 | 0.06 | 0.01 | 100 | 0 | 15 | 0.2 |
| TX06V7266 | 0 | 8 | 0.4 | 7 | 0.6 | 0.16 | 0.01 | 0.06 | 0.01 | 100 | 0 | 14 | 0.8 |
| OK1067071 | 0 | 8 | 0.4 | 7 | 0.5 | 0.16 | 0.01 | 0.06 | 0.01 | 100 | 0 | 14 | 0.9 |
| OK1067274 | 0 | 8 | 0.4 | 7 | 0.4 | 0.16 | 0.01 | 0.06 | 0.01 | 100 | 0 | 15 | 0.2 |
| OK1068002 | 0 | 8 | 0.6 | 7 | 0.5 | 0.16 | 0.01 | 0.06 | 0.01 | 100 | 0 | 15 | 0.4 |
| OK1068009 | 0 | 8 | 0.8 | 7 | 0.5 | 0.16 | 0.01 | 0.05 | 0.00 | 100 | 0 | 15 | 0.3 |
| OK1068026 | 0 | 8 | 0.7 | 7 | 0.4 | 0.16 | 0.01 | 0.06 | 0.01 | 100 | 0 | 15 | 0.8 |
| OK1068112 | 0 | 8 | 0.5 | 7 | 0.5 | 0.15 | 0.01 | 0.06 | 0.01 | 100 | 0 | 14 | 0.6 |
| OK1070275 | 0 | 8 | 0.5 | 7 | 0.4 | 0.16 | 0.01 | 0.06 | 0.01 | 100 | 0 | 14 | 0.3 |
| OK1070267 | 0 | 8 | 0.5 | 7 | 0.5 | 0.16 | 0.01 | 0.06 | 0.01 | 100 | 0 | 15 | 0.4 |
| OK09634 | 0 | 8 | 0.4 | 7 | 0.5 | 0.15 | 0.01 | 0.06 | 0.01 | 100 | 0 | 14 | 0.3 |
| OK10119 | 0 | 8 | 0.4 | 7 | 0.5 | 0.15 | 0.01 | 0.06 | 0.00 | 100 | 0 | 14 | 0.3 |
| GALLAGHER | 0 | 8 | 0.6 | 7 | 0.5 | 0.16 | 0.01 | 0.06 | 0.01 | 100 | 0 | 15 | 0.3 |
| OK07231 | 0 | 8 | 0.5 | 7 | 0.5 | 0.16 | 0.01 | 0.06 | 0.01 | 100 | 0 | 15 | 0.6 |
| OK07S117 | 0 | 8 | 0.5 | 7 | 0.6 | 0.15 | 0.01 | 0.06 | 0.01 | 100 | 0 | 15 | 0.3 |
| OK08328 | 0 | 8 | 0.6 | 7 | 0.5 | 0.16 | 0.01 | 0.06 | 0.01 | 100 | 0 | 15 | 0.7 |
| BIG_SKY | 0 | 8 | 0.5 | 6 | 0.4 | 0.16 | 0.01 | 0.06 | 0.01 | 100 | 0 | 14 | 0.2 |
| DANBY | 0 | 8 | 0.3 | 7 | 0.3 | 0.16 | 0.01 | 0.06 | 0.01 | 100 | 0 | 15 | 0.6 |
| E2041 | 0 | 8 | 0.5 | 7 | 0.5 | 0.15 | 0.01 | 0.06 | 0.01 | 100 | 0 | 15 | 0.8 |
| DENALI | 0 | 8 | 0.4 | 7 | 0.4 | 0.16 | 0.01 | 0.06 | 0.01 | 100 | 0 | 14 | 1.2 |
| CO050337-2 | 0 | 8 | 0.7 | 7 | 0.3 | 0.15 | 0.01 | 0.06 | 0.00 | 100 | 0 | 14 | 1.3 |
| BYRD | 0 | 8 | 0.3 | 7 | 0.4 | 0.16 | 0.01 | 0.06 | 0.01 | 100 | 0 | 15 | 0.3 |
| CO07W245 | 0 | 8 | 0.5 | 7 | 0.5 | 0.15 | 0.01 | 0.06 | 0.00 | 100 | 0 | 15 | 0.2 |
| MCGILL | 0 | 8 | 0.1 | 7 | 0.3 | 0.15 | 0.01 | 0.05 | 0.01 | 100 | 0 | 15 | 0.9 |
| NE02558 | 0 | 8 | 0.6 | 7 | 0.4 | 0.16 | 0.01 | 0.06 | 0.01 | 100 | 0 | 15 | 0.6 |
| NW03666 | 0 | 8 | 0.3 | 7 | 0.5 | 0.16 | 0.01 | 0.06 | 0.01 | 100 | 0 | 15 | 0.3 |
| NE04490 | 0 | 8 | 0.4 | 7 | 0.5 | 0.16 | 0.01 | 0.06 | 0.01 | 100 | 0 | 14 | 0.6 |
| NE05430 | 0 | 8 | 0.5 | 7 | 0.5 | 0.16 | 0.01 | 0.06 | 0.01 | 100 | 0 | 15 | 0.3 |
| NE05496 | 0 | 8 | 0.3 | 7 | 0.3 | 0.16 | 0.01 | 0.06 | 0.01 | 100 | 0 | 15 | 0.4 |
| NE05548 | 0 | 8 | 0.6 | 7 | 0.5 | 0.16 | 0.01 | 0.06 | 0.01 | 100 | 0 | 15 | 0.7 |
| NE06545 | 0 | 8 | 0.4 | 7 | 0.3 | 0.16 | 0.01 | 0.06 | 0.01 | 100 | 0 | 15 | 0.4 |
| NE06607 | 0 | 8 | 0.5 | 7 | 0.5 | 0.16 | 0.01 | 0.06 | 0.01 | 100 | 0 | 15 | 0.5 |
| ROBIDOUX | 0 | 8 | 0.5 | 7 | 0.4 | 0.16 | 0.01 | 0.06 | 0.01 | 100 | 0 | 15 | 0.4 |
| NI06736 | 0 | 8 | 0.5 | 7 | 0.5 | 0.16 | 0.01 | 0.06 | 0.01 | 100 | 0 | 15 | 0.4 |
| NI06737 | 0 | 8 | 0.4 | 7 | 0.3 | 0.16 | 0.01 | 0.06 | 0.01 | 100 | 0 | 14 | 0.8 |
| NI07703 | 0 | 8 | 0.6 | 7 | 0.5 | 0.16 | 0.01 | 0.06 | 0.01 | 100 | 0 | 14 | 0.7 |
| NI08707 | 0 | 8 | 0.6 | 7 | 0.4 | 0.16 | 0.01 | 0.06 | 0.01 | 100 | 0 | 14 | 1.1 |
| NI08708 | 0 | 8 | 0.7 | 7 | 0.5 | 0.16 | 0.01 | 0.06 | 0.01 | 100 | 0 | 14 | 0.7 |
| EVEREST | 0 | 8 | 0.6 | 7 | 0.4 | 0.16 | 0.01 | 0.06 | 0.01 | 100 | 0 | 15 | 0.5 |
| TRIUMPH64 | 60 | 6 | 0.6 | 4 | 0.3 | 0.12 | 0.02 | 0.04 | 0.00 | 66 | 11 | 9 | 1.0 |
| CHISHOLM | 60 | 6 | 0.3 | 4 | 0.6 | 0.14 | 0.01 | 0.05 | 0.01 | 77 | 9 | 10 | 0.7 |
| CUSTER | 60 | 6 | 0.5 | 4 | 0.3 | 0.13 | 0.01 | 0.04 | 0.00 | 75 | 15 | 9 | 0.4 |
| 2174-05 | 60 | 5 | 0.6 | 4 | 0.6 | 0.14 | 0.01 | 0.04 | 0.01 | 58 | 9 | 9 | 0.8 |
| INTRADA | 60 | 5 | 0.4 | 4 | 0.4 | 0.13 | 0.01 | 0.03 | 0.00 | 50 | 8 | 8 | 0.4 |
| OK101 | 60 | 5 | 0.3 | 4 | 0.4 | 0.14 | 0.01 | 0.03 | 0.01 | 65 | 15 | 8 | 0.5 |
| OK102 | 60 | 6 | 0.5 | 4 | 0.7 | 0.13 | 0.01 | 0.03 | 0.01 | 60 | 12 | 9 | 0.7 |
| ENDURANCE | 60 | 6 | 0.2 | 5 | 0.3 | 0.15 | 0.01 | 0.04 | 0.00 | 76 | 10 | 11 | 0.4 |
| DELIVER | 60 | 5 | 0.4 | 4 | 0.8 | 0.14 | 0.01 | 0.03 | 0.01 | 58 | 17 | 9 | 1.0 |
| OK_BULLET | 60 | 5 | 0.5 | 4 | 0.6 | 0.14 | 0.01 | 0.03 | 0.01 | 57 | 14 | 8 | 0.2 |
| CENTERFIELD | 60 | 5 | 0.4 | 4 | 0.6 | 0.12 | 0.01 | 0.03 | 0.01 | 59 | 11 | 8 | 0.6 |
| GUYMON | 60 | 7 | 1.0 | 6 | 0.8 | 0.14 | 0.01 | 0.05 | 0.01 | 80 | 15 | 12 | 1.1 |
| DUSTER | 60 | 5 | 0.4 | 4 | 0.5 | 0.14 | 0.01 | 0.03 | 0.01 | 52 | 13 | 8 | 0.5 |
| OK_RISING | 60 | 5 | 0.4 | 4 | 0.5 | 0.13 | 0.01 | 0.05 | 0.01 | 81 | 14 | 8 | 0.5 |
| OK02405 | 60 | 5 | 0.3 | 4 | 0.6 | 0.14 | 0.01 | 0.03 | 0.01 | 60 | 18 | 9 | 0.9 |
| PETE | 60 | 5 | 0.3 | 4 | 0.6 | 0.14 | 0.00 | 0.04 | 0.01 | 75 | 13 | 9 | 0.4 |
| BILLINGS | 60 | 5 | 0.4 | 4 | 0.5 | 0.13 | 0.01 | 0.04 | 0.01 | 65 | 14 | 8 | 0.7 |
| OK04505 | 60 | 5 | 0.5 | 4 | 0.3 | 0.14 | 0.01 | 0.04 | 0.01 | 78 | 13 | 9 | 0.5 |
| OK04525 | 60 | 6 | 0.3 | 4 | 0.4 | 0.13 | 0.01 | 0.03 | 0.00 | 51 | 9 | 9 | 0.4 |
| OK04507 | 60 | 6 | 0.4 | 4 | 0.3 | 0.14 | 0.01 | 0.04 | 0.01 | 71 | 14 | 9 | 0.3 |
| OK05830 | 60 | 6 | 0.4 | 4 | 0.8 | 0.13 | 0.01 | 0.03 | 0.00 | 51 | 9 | 9 | 0.6 |
| OK04111 | 60 | 5 | 0.5 | 4 | 0.8 | 0.14 | 0.01 | 0.04 | 0.01 | 64 | 14 | 9 | 0.5 |
| OK04415 | 60 | 5 | 0.3 | 4 | 0.7 | 0.13 | 0.01 | 0.03 | 0.01 | 59 | 15 | 8 | 0.4 |
| OK05711W | 60 | 5 | 0.4 | 4 | 0.4 | 0.15 | 0.01 | 0.04 | 0.01 | 71 | 14 | 9 | 0.6 |
| OK05723W | 60 | 5 | 0.3 | 5 | 0.5 | 0.15 | 0.01 | 0.04 | 0.01 | 76 | 11 | 9 | 0.4 |
| OK05108 | 60 | 6 | 0.7 | 5 | 0.9 | 0.15 | 0.01 | 0.05 | 0.01 | 71 | 16 | 10 | 0.3 |
| OK05122 | 60 | 5 | 0.4 | 4 | 0.4 | 0.12 | 0.02 | 0.04 | 0.01 | 77 | 13 | 8 | 0.5 |
| OK05526 | 60 | 5 | 0.5 | 4 | 0.7 | 0.13 | 0.01 | 0.04 | 0.01 | 62 | 12 | 9 | 0.2 |
| OK05134 | 60 | 5 | 0.5 | 4 | 0.5 | 0.13 | 0.01 | 0.04 | 0.01 | 65 | 14 | 9 | 0.7 |
| OK05303 | 60 | 5 | 0.3 | 4 | 0.6 | 0.14 | 0.01 | 0.03 | 0.01 | 65 | 10 | 8 | 0.5 |
| OK05312 | 60 | 5 | 0.3 | 4 | 0.6 | 0.14 | 0.01 | 0.03 | 0.00 | 57 | 11 | 9 | 0.7 |
| OK05511 | 60 | 5 | 0.4 | 4 | 0.7 | 0.13 | 0.01 | 0.03 | 0.00 | 59 | 11 | 9 | 0.4 |
| OK05204 | 60 | 5 | 0.3 | 4 | 0.6 | 0.13 | 0.01 | 0.04 | 0.01 | 65 | 11 | 9 | 0.2 |
| GARRISON | 60 | 5 | 0.3 | 4 | 0.4 | 0.14 | 0.01 | 0.05 | 0.01 | 77 | 8 | 9 | 0.2 |
| OK06114 | 60 | 6 | 0.3 | 4 | 0.6 | 0.14 | 0.01 | 0.03 | 0.00 | 54 | 7 | 9 | 0.3 |
| OK06210 | 60 | 5 | 0.4 | 5 | 0.4 | 0.15 | 0.01 | 0.04 | 0.01 | 74 | 9 | 9 | 0.5 |
| OK06319 | 60 | 5 | 0.5 | 4 | 0.5 | 0.13 | 0.01 | 0.04 | 0.01 | 72 | 11 | 9 | 0.7 |
| OK06318 | 60 | 6 | 0.5 | 4 | 0.2 | 0.13 | 0.01 | 0.04 | 0.01 | 74 | 9 | 8 | 1.6 |
| OK06336 | 60 | 5 | 0.3 | 4 | 0.5 | 0.12 | 0.01 | 0.03 | 0.00 | 58 | 8 | 9 | 0.1 |
| AGATE | 60 | 5 | 0.3 | 4 | 0.3 | 0.13 | 0.02 | 0.03 | 0.00 | 53 | 9 | 8 | 0.3 |
| ALLIANCE | 60 | 6 | 0.3 | 4 | 0.2 | 0.12 | 0.01 | 0.03 | 0.00 | 56 | 9 | 9 | 0.8 |
| ANTELOPE | 60 | 5 | 0.3 | 4 | 0.5 | 0.13 | 0.01 | 0.03 | 0.00 | 53 | 10 | 9 | 0.3 |
| ARAPAHOE | 60 | 6 | 0.3 | 4 | 0.5 | 0.13 | 0.01 | 0.03 | 0.00 | 57 | 8 | 9 | 0.5 |
| BENNETT | 60 | 6 | 0.2 | 5 | 0.5 | 0.14 | 0.01 | 0.04 | 0.01 | 77 | 13 | 10 | 0.4 |
| BUCKSKIN | 60 | 5 | 0.4 | 4 | 0.6 | 0.14 | 0.01 | 0.04 | 0.01 | 70 | 17 | 9 | 0.6 |
| CENTURK78 | 60 | 5 | 0.3 | 4 | 0.3 | 0.14 | 0.01 | 0.05 | 0.00 | 78 | 12 | 9 | 0.3 |
| CHEYENNE | 60 | 5 | 0.4 | 4 | 0.5 | 0.14 | 0.01 | 0.04 | 0.01 | 73 | 10 | 9 | 0.6 |
| COLT | 60 | 5 | 0.4 | 4 | 0.7 | 0.13 | 0.02 | 0.03 | 0.00 | 58 | 11 | 9 | 0.2 |
| COUGAR | 60 | 5 | 0.4 | 4 | 0.7 | 0.13 | 0.02 | 0.03 | 0.00 | 58 | 8 | 9 | 0.9 |
| CULVER | 60 | 5 | 0.4 | 4 | 0.7 | 0.13 | 0.01 | 0.03 | 0.01 | 61 | 12 | 9 | 0.7 |
| GAGE | 60 | 7 | 0.7 | 5 | 0.7 | 0.14 | 0.01 | 0.04 | 0.01 | 81 | 16 | 11 | 0.6 |
| GOODSTREAK | 60 | 5 | 0.4 | 4 | 0.4 | 0.15 | 0.01 | 0.04 | 0.01 | 75 | 10 | 8 | 0.6 |
| HALLAM | 60 | 4 | 0.4 | 3 | 0.2 | 0.13 | 0.01 | 0.04 | 0.01 | 66 | 12 | 7 | 0.2 |
| HARRY | 60 | 5 | 0.4 | 4 | 0.6 | 0.14 | 0.01 | 0.03 | 0.00 | 54 | 5 | 9 | 0.8 |
| HOMESTEAD | 60 | 6 | 0.5 | 4 | 0.3 | 0.14 | 0.01 | 0.04 | 0.01 | 73 | 9 | 9 | 0.7 |
| INFINITY_CL | 60 | 5 | 0.3 | 4 | 0.6 | 0.13 | 0.01 | 0.04 | 0.01 | 64 | 10 | 9 | 0.5 |
| KHARKOF | 60 | 5 | 0.4 | 4 | 0.4 | 0.14 | 0.01 | 0.05 | 0.00 | 76 | 8 | 9 | 0.3 |
| MILLENNIUM | 60 | 5 | 0.3 | 4 | 0.7 | 0.14 | 0.01 | 0.04 | 0.01 | 61 | 10 | 8 | 0.5 |
| CAMELOT | 60 | 5 | 0.5 | 4 | 0.5 | 0.13 | 0.01 | 0.04 | 0.01 | 60 | 9 | 9 | 0.6 |
| OVERLAND | 60 | 5 | 0.4 | 4 | 0.4 | 0.14 | 0.01 | 0.03 | 0.01 | 58 | 11 | 9 | 0.4 |
| NE99495 | 60 | 4 | 0.2 | 4 | 0.2 | 0.14 | 0.01 | 0.05 | 0.01 | 74 | 9 | 8 | 0.2 |
| NIOBRARA | 60 | 5 | 0.3 | 4 | 0.2 | 0.14 | 0.01 | 0.04 | 0.01 | 71 | 10 | 8 | 0.5 |
| NUPLAINS | 60 | 5 | 0.4 | 4 | 0.3 | 0.13 | 0.02 | 0.04 | 0.01 | 60 | 13 | 8 | 0.7 |
| PRONGHORN | 60 | 5 | 0.4 | 4 | 0.6 | 0.14 | 0.01 | 0.03 | 0.00 | 60 | 13 | 8 | 0.5 |
| RAWHIDE | 60 | 5 | 0.4 | 4 | 0.5 | 0.13 | 0.01 | 0.04 | 0.01 | 63 | 14 | 9 | 0.3 |
| REDLAND | 60 | 5 | 0.3 | 4 | 0.7 | 0.13 | 0.01 | 0.03 | 0.00 | 60 | 14 | 9 | 0.2 |
| SCOUT66 | 60 | 5 | 0.4 | 4 | 0.4 | 0.14 | 0.01 | 0.04 | 0.01 | 73 | 11 | 9 | 0.6 |
| SIOUXLAND | 60 | 6 | 0.4 | 4 | 0.6 | 0.14 | 0.01 | 0.04 | 0.01 | 61 | 13 | 8 | 0.6 |
| TURKEY_NEBSEL | 60 | 5 | 0.5 | 4 | 0.5 | 0.13 | 0.02 | 0.04 | 0.01 | 65 | 12 | 9 | 0.4 |
| VISTA | 60 | 5 | 0.5 | 4 | 0.5 | 0.13 | 0.02 | 0.03 | 0.00 | 55 | 8 | 9 | 0.3 |
| WAHOO | 60 | 5 | 0.3 | 4 | 0.6 | 0.12 | 0.02 | 0.03 | 0.01 | 58 | 11 | 9 | 0.7 |
| WARRIOR | 60 | 5 | 0.4 | 4 | 0.5 | 0.14 | 0.01 | 0.04 | 0.01 | 72 | 9 | 9 | 0.4 |
| WESLEY | 60 | 5 | 0.3 | 4 | 0.2 | 0.14 | 0.01 | 0.04 | 0.01 | 73 | 12 | 8 | 0.4 |
| WICHITA | 60 | 5 | 0.5 | 4 | 0.4 | 0.14 | 0.00 | 0.04 | 0.01 | 74 | 9 | 9 | 1.0 |
| WINDSTAR | 60 | 5 | 0.5 | 4 | 0.5 | 0.13 | 0.01 | 0.03 | 0.01 | 60 | 8 | 9 | 0.8 |
| LANCER | 60 | 5 | 0.5 | 4 | 0.3 | 0.14 | 0.01 | 0.05 | 0.01 | 75 | 7 | 8 | 0.6 |
| ANTON | 60 | 6 | 0.4 | 4 | 0.3 | 0.13 | 0.02 | 0.04 | 0.01 | 62 | 11 | 8 | 0.4 |
| MACE | 60 | 5 | 0.4 | 4 | 0.3 | 0.11 | 0.02 | 0.04 | 0.00 | 64 | 9 | 8 | 0.5 |
| TAM107-R7 | 60 | 5 | 0.4 | 4 | 0.5 | 0.13 | 0.01 | 0.03 | 0.00 | 60 | 14 | 9 | 0.6 |
| ARLIN | 60 | 6 | 0.5 | 4 | 0.5 | 0.14 | 0.01 | 0.04 | 0.01 | 68 | 16 | 9 | 0.4 |
| ALICE | 60 | 5 | 0.4 | 4 | 0.3 | 0.13 | 0.02 | 0.03 | 0.00 | 59 | 11 | 9 | 0.3 |
| DARRELL | 60 | 5 | 0.3 | 4 | 0.3 | 0.13 | 0.01 | 0.03 | 0.00 | 57 | 10 | 9 | 0.4 |
| EXPEDITION | 60 | 5 | 0.3 | 4 | 0.3 | 0.13 | 0.01 | 0.03 | 0.01 | 60 | 10 | 8 | 0.3 |
| WENDY | 60 | 5 | 0.3 | 4 | 0.2 | 0.14 | 0.01 | 0.04 | 0.01 | 68 | 10 | 8 | 0.3 |
| SD00111-9 | 60 | 5 | 0.4 | 4 | 0.3 | 0.13 | 0.02 | 0.03 | 0.00 | 57 | 8 | 8 | 0.5 |
| SD01237 | 60 | 5 | 0.3 | 4 | 0.5 | 0.12 | 0.02 | 0.03 | 0.00 | 57 | 9 | 9 | 0.4 |
| SD01058 | 60 | 5 | 0.4 | 4 | 0.5 | 0.13 | 0.02 | 0.03 | 0.00 | 58 | 9 | 9 | 0.2 |
| SD05118 | 60 | 5 | 0.5 | 4 | 0.4 | 0.14 | 0.01 | 0.04 | 0.01 | 72 | 12 | 9 | 0.6 |
| SD05210 | 60 | 5 | 0.3 | 4 | 0.6 | 0.13 | 0.02 | 0.03 | 0.00 | 55 | 10 | 8 | 1.0 |
| SD05W018 | 60 | 5 | 0.3 | 4 | 0.6 | 0.13 | 0.01 | 0.03 | 0.00 | 56 | 9 | 9 | 0.4 |
| NEKOTA | 60 | 5 | 0.5 | 4 | 0.3 | 0.14 | 0.01 | 0.05 | 0.01 | 78 | 11 | 9 | 0.4 |
| TANDEM | 60 | 5 | 0.4 | 4 | 0.5 | 0.13 | 0.01 | 0.04 | 0.01 | 71 | 9 | 9 | 0.2 |
| CRIMSON | 60 | 5 | 0.4 | 4 | 0.5 | 0.13 | 0.01 | 0.03 | 0.00 | 57 | 11 | 9 | 0.3 |
| ROSE | 60 | 5 | 0.3 | 4 | 0.5 | 0.13 | 0.02 | 0.03 | 0.00 | 57 | 10 | 8 | 0.8 |
| DAWN | 60 | 5 | 0.4 | 4 | 0.3 | 0.14 | 0.01 | 0.05 | 0.01 | 76 | 12 | 9 | 0.3 |
| WINOKA | 60 | 5 | 0.4 | 4 | 0.3 | 0.14 | 0.01 | 0.03 | 0.00 | 57 | 17 | 8 | 0.4 |
| NELL | 60 | 5 | 0.5 | 4 | 0.5 | 0.14 | 0.01 | 0.04 | 0.00 | 73 | 12 | 9 | 0.3 |
| RITA | 60 | 5 | 0.5 | 4 | 0.5 | 0.13 | 0.02 | 0.03 | 0.00 | 61 | 12 | 9 | 0.6 |
| BRONZE | 60 | 5 | 0.3 | 4 | 0.5 | 0.11 | 0.02 | 0.03 | 0.00 | 56 | 8 | 9 | 0.2 |
| HUME | 60 | 5 | 0.4 | 4 | 0.7 | 0.13 | 0.02 | 0.03 | 0.00 | 52 | 12 | 9 | 0.0 |
| GENT | 60 | 5 | 0.4 | 4 | 0.4 | 0.13 | 0.02 | 0.03 | 0.01 | 62 | 16 | 8 | 0.6 |
| HARDING | 60 | 5 | 0.4 | 4 | 0.4 | 0.13 | 0.02 | 0.03 | 0.00 | 58 | 10 | 8 | 0.8 |
| HV9W03-1551WP | 60 | 5 | 0.5 | 4 | 0.2 | 0.14 | 0.01 | 0.04 | 0.01 | 76 | 13 | 9 | 0.6 |
| G1878 | 60 | 5 | 0.4 | 4 | 0.5 | 0.13 | 0.01 | 0.05 | 0.01 | 75 | 12 | 9 | 0.5 |
| HV9W03-1379R | 60 | 5 | 0.5 | 4 | 0.4 | 0.13 | 0.02 | 0.03 | 0.00 | 52 | 7 | 9 | 0.9 |
| HV9W03-1596R | 60 | 5 | 0.6 | 4 | 0.5 | 0.13 | 0.01 | 0.03 | 0.00 | 55 | 11 | 8 | 0.6 |
| HV9W05-1280R | 60 | 5 | 0.4 | 4 | 0.5 | 0.13 | 0.02 | 0.04 | 0.01 | 71 | 15 | 9 | 0.4 |
| HV9W06-504 | 60 | 5 | 0.4 | 4 | 0.7 | 0.13 | 0.01 | 0.03 | 0.00 | 61 | 11 | 9 | 0.4 |
| SPARTAN | 60 | 5 | 0.5 | 4 | 0.4 | 0.13 | 0.01 | 0.04 | 0.01 | 64 | 16 | 9 | 0.8 |
| HV906-865 | 60 | 5 | 0.5 | 4 | 0.3 | 0.13 | 0.01 | 0.05 | 0.01 | 71 | 8 | 9 | 0.4 |
| TARKIO | 60 | 5 | 0.5 | 4 | 0.5 | 0.13 | 0.01 | 0.03 | 0.00 | 57 | 10 | 9 | 0.6 |
| SMOKYHILL | 60 | 6 | 0.5 | 4 | 0.5 | 0.12 | 0.02 | 0.04 | 0.01 | 68 | 13 | 8 | 0.6 |
| SHOCKER | 60 | 5 | 0.4 | 4 | 0.4 | 0.13 | 0.01 | 0.03 | 0.01 | 57 | 11 | 8 | 0.5 |
| VONA | 60 | 5 | 0.4 | 4 | 0.4 | 0.13 | 0.02 | 0.03 | 0.00 | 54 | 9 | 8 | 0.6 |
| CO940610 | 60 | 5 | 0.4 | 4 | 0.5 | 0.12 | 0.02 | 0.03 | 0.00 | 55 | 11 | 8 | 0.6 |
| AVALANCHE | 60 | 6 | 0.9 | 5 | 0.7 | 0.13 | 0.01 | 0.04 | 0.01 | 69 | 15 | 10 | 0.8 |
| BOND_CL | 60 | 5 | 0.5 | 4 | 0.6 | 0.13 | 0.02 | 0.03 | 0.00 | 59 | 10 | 9 | 0.5 |
| PLATTE | 60 | 5 | 0.5 | 4 | 0.6 | 0.13 | 0.01 | 0.03 | 0.00 | 61 | 11 | 9 | 0.4 |
| LINDON | 60 | 5 | 0.4 | 4 | 0.4 | 0.13 | 0.01 | 0.04 | 0.01 | 62 | 11 | 8 | 0.7 |
| CO03W043 | 60 | 5 | 0.5 | 4 | 0.5 | 0.13 | 0.01 | 0.03 | 0.01 | 60 | 12 | 9 | 0.4 |
| SNOWMASS | 60 | 5 | 0.5 | 4 | 0.5 | 0.13 | 0.02 | 0.03 | 0.01 | 59 | 10 | 8 | 0.4 |
| THUNDER_CL | 60 | 5 | 0.4 | 4 | 0.2 | 0.13 | 0.01 | 0.04 | 0.01 | 72 | 10 | 9 | 0.5 |
| CO04025 | 60 | 5 | 0.4 | 4 | 0.5 | 0.13 | 0.01 | 0.03 | 0.00 | 57 | 11 | 9 | 0.7 |
| CO04393 | 60 | 5 | 0.5 | 4 | 0.4 | 0.13 | 0.01 | 0.04 | 0.00 | 74 | 11 | 9 | 0.4 |
| CO04499 | 60 | 5 | 0.4 | 4 | 0.4 | 0.14 | 0.01 | 0.03 | 0.00 | 54 | 9 | 9 | 0.6 |
| CO04W320 | 60 | 6 | 0.9 | 5 | 1.0 | 0.14 | 0.01 | 0.04 | 0.01 | 56 | 17 | 9 | 0.7 |
| LAMAR | 60 | 5 | 0.4 | 4 | 0.5 | 0.14 | 0.01 | 0.04 | 0.01 | 69 | 10 | 9 | 0.8 |
| CARSON | 60 | 6 | 0.9 | 5 | 0.8 | 0.15 | 0.01 | 0.04 | 0.01 | 59 | 8 | 10 | 0.1 |
| HAIL | 60 | 5 | 0.2 | 4 | 0.7 | 0.14 | 0.01 | 0.03 | 0.00 | 52 | 5 | 8 | 0.7 |
| SANDY | 60 | 5 | 0.4 | 4 | 0.5 | 0.13 | 0.01 | 0.03 | 0.00 | 56 | 8 | 9 | 0.7 |
| DUKE | 60 | 5 | 0.4 | 4 | 0.5 | 0.13 | 0.01 | 0.03 | 0.00 | 57 | 8 | 9 | 0.4 |
| HALT | 60 | 5 | 0.5 | 4 | 0.4 | 0.14 | 0.01 | 0.05 | 0.01 | 73 | 10 | 9 | 0.2 |
| HATCHER | 60 | 5 | 0.4 | 4 | 0.6 | 0.13 | 0.01 | 0.03 | 0.00 | 57 | 8 | 8 | 0.5 |
| PRAIRIE_RED | 60 | 5 | 0.5 | 5 | 0.4 | 0.13 | 0.01 | 0.04 | 0.01 | 63 | 14 | 9 | 0.4 |
| ABOVE | 60 | 5 | 0.3 | 4 | 0.6 | 0.13 | 0.00 | 0.03 | 0.00 | 57 | 9 | 9 | 0.6 |
| CO03064 | 60 | 5 | 0.3 | 4 | 0.6 | 0.13 | 0.01 | 0.04 | 0.01 | 68 | 13 | 9 | 0.6 |
| BILL_BROWN | 60 | 5 | 0.4 | 4 | 0.5 | 0.13 | 0.01 | 0.03 | 0.01 | 59 | 12 | 8 | 0.3 |
| RIPPER | 60 | 5 | 0.4 | 4 | 0.6 | 0.12 | 0.02 | 0.03 | 0.00 | 53 | 6 | 9 | 0.3 |
| PROWERS | 60 | 5 | 0.4 | 4 | 0.3 | 0.13 | 0.01 | 0.04 | 0.01 | 64 | 11 | 8 | 0.4 |
| AKRON | 60 | 5 | 0.5 | 4 | 0.5 | 0.13 | 0.01 | 0.04 | 0.01 | 67 | 14 | 9 | 0.6 |
| JULES | 60 | 5 | 0.4 | 4 | 0.3 | 0.14 | 0.01 | 0.04 | 0.01 | 66 | 11 | 9 | 0.9 |
| YUMA | 60 | 5 | 0.5 | 4 | 0.5 | 0.13 | 0.01 | 0.04 | 0.01 | 66 | 11 | 8 | 0.7 |
| TAMW-101 | 60 | 5 | 0.4 | 4 | 0.3 | 0.13 | 0.01 | 0.04 | 0.01 | 67 | 10 | 8 | 0.4 |
| TAM105 | 60 | 5 | 0.4 | 4 | 0.4 | 0.14 | 0.01 | 0.03 | 0.01 | 58 | 10 | 9 | 0.4 |
| TAM107 | 60 | 5 | 0.4 | 4 | 0.5 | 0.13 | 0.01 | 0.04 | 0.01 | 65 | 10 | 8 | 0.2 |
| TAM109 | 60 | 5 | 0.4 | 4 | 0.5 | 0.13 | 0.01 | 0.04 | 0.00 | 73 | 10 | 9 | 0.3 |
| TAM110 | 60 | 5 | 0.4 | 4 | 0.2 | 0.14 | 0.01 | 0.04 | 0.00 | 70 | 10 | 9 | 0.5 |
| TAM111 | 60 | 5 | 0.5 | 4 | 0.4 | 0.14 | 0.01 | 0.04 | 0.01 | 76 | 17 | 9 | 0.5 |
| TAM112 | 60 | 5 | 0.5 | 4 | 0.2 | 0.12 | 0.01 | 0.04 | 0.00 | 77 | 10 | 8 | 0.4 |
| TAM200 | 60 | 5 | 0.3 | 4 | 0.3 | 0.13 | 0.02 | 0.04 | 0.01 | 77 | 13 | 8 | 0.3 |
| TAM202 | 60 | 5 | 0.5 | 4 | 0.3 | 0.13 | 0.01 | 0.03 | 0.01 | 62 | 12 | 7 | 0.8 |
| TAM203 | 60 | 5 | 0.5 | 4 | 0.2 | 0.13 | 0.01 | 0.04 | 0.00 | 75 | 8 | 8 | 0.5 |
| TAM302 | 60 | 5 | 0.4 | 4 | 0.4 | 0.13 | 0.01 | 0.04 | 0.01 | 71 | 9 | 8 | 0.3 |
| TAM303 | 60 | 5 | 0.4 | 4 | 0.3 | 0.13 | 0.01 | 0.03 | 0.00 | 63 | 14 | 8 | 0.6 |
| TAM304 | 60 | 5 | 0.4 | 4 | 0.2 | 0.13 | 0.01 | 0.04 | 0.01 | 67 | 11 | 8 | 0.4 |
| TAM400 | 60 | 5 | 0.3 | 4 | 0.3 | 0.14 | 0.01 | 0.03 | 0.01 | 65 | 10 | 7 | 0.2 |
| LOCKETT | 60 | 5 | 0.5 | 4 | 0.4 | 0.14 | 0.01 | 0.03 | 0.01 | 63 | 11 | 9 | 0.6 |
| STURDY | 60 | 5 | 0.3 | 4 | 0.5 | 0.13 | 0.01 | 0.04 | 0.01 | 70 | 14 | 8 | 0.6 |
| STURDY_2K | 60 | 5 | 0.5 | 4 | 0.6 | 0.13 | 0.01 | 0.04 | 0.00 | 79 | 12 | 9 | 0.3 |
| MIT | 60 | 5 | 0.4 | 4 | 0.3 | 0.13 | 0.02 | 0.04 | 0.01 | 74 | 14 | 8 | 0.6 |
| CAPROCK | 60 | 5 | 0.4 | 4 | 0.4 | 0.13 | 0.01 | 0.04 | 0.00 | 71 | 10 | 9 | 0.5 |
| TX01A5936 | 60 | 5 | 0.6 | 4 | 0.2 | 0.13 | 0.01 | 0.03 | 0.00 | 61 | 13 | 8 | 0.4 |
| TAM401 | 60 | 5 | 0.4 | 4 | 0.5 | 0.13 | 0.01 | 0.04 | 0.00 | 72 | 10 | 9 | 0.3 |
| TX02A0252 | 60 | 5 | 0.5 | 4 | 0.4 | 0.13 | 0.01 | 0.04 | 0.01 | 80 | 12 | 9 | 0.5 |
| TX03A0148 | 60 | 5 | 0.4 | 4 | 0.6 | 0.13 | 0.01 | 0.04 | 0.01 | 71 | 12 | 8 | 0.2 |
| TX03A0563 | 60 | 5 | 0.4 | 4 | 0.5 | 0.13 | 0.01 | 0.04 | 0.01 | 64 | 12 | 8 | 0.8 |
| TX04A001246 | 60 | 5 | 0.3 | 4 | 0.5 | 0.12 | 0.02 | 0.05 | 0.01 | 84 | 13 | 8 | 0.7 |
| TX01V5134RC-3 | 60 | 4 | 0.4 | 4 | 0.3 | 0.13 | 0.01 | 0.04 | 0.00 | 76 | 11 | 8 | 0.1 |
| TX04M410164 | 60 | 5 | 0.4 | 4 | 0.4 | 0.13 | 0.01 | 0.04 | 0.01 | 66 | 13 | 8 | 0.6 |
| TX04M410211 | 60 | 6 | 0.6 | 5 | 0.8 | 0.15 | 0.01 | 0.04 | 0.01 | 59 | 17 | 11 | 0.6 |
| TX04V075080 | 60 | 5 | 0.4 | 4 | 0.4 | 0.13 | 0.01 | 0.04 | 0.00 | 72 | 8 | 7 | 0.4 |
| TX99A0153-1 | 60 | 5 | 0.4 | 4 | 0.6 | 0.14 | 0.01 | 0.04 | 0.01 | 63 | 9 | 8 | 0.6 |
| TX01M5009-28 | 60 | 5 | 0.4 | 4 | 0.5 | 0.13 | 0.01 | 0.04 | 0.00 | 75 | 8 | 8 | 0.8 |
| TX00V1131 | 60 | 5 | 0.4 | 4 | 0.6 | 0.13 | 0.01 | 0.04 | 0.00 | 71 | 9 | 9 | 0.3 |
| TX99U8618 | 60 | 4 | 0.4 | 4 | 0.4 | 0.13 | 0.01 | 0.04 | 0.01 | 64 | 12 | 8 | 0.8 |
| TX96D1073 | 60 | 5 | 0.4 | 4 | 0.4 | 0.13 | 0.01 | 0.04 | 0.01 | 69 | 11 | 8 | 0.4 |
| 2180 | 60 | 5 | 0.3 | 4 | 0.6 | 0.14 | 0.01 | 0.03 | 0.00 | 58 | 9 | 9 | 0.6 |
| HG-9 | 60 | 5 | 0.4 | 4 | 0.5 | 0.14 | 0.01 | 0.04 | 0.01 | 69 | 15 | 8 | 0.4 |
| TX86A5606 | 60 | 6 | 0.7 | 5 | 0.7 | 0.15 | 0.01 | 0.05 | 0.01 | 69 | 9 | 11 | 0.5 |
| TX86A8072 | 60 | 5 | 0.4 | 4 | 0.4 | 0.14 | 0.01 | 0.04 | 0.00 | 77 | 9 | 8 | 0.4 |
| CREST | 60 | 5 | 0.5 | 4 | 0.4 | 0.13 | 0.01 | 0.04 | 0.01 | 68 | 12 | 9 | 0.9 |
| ROSEBUD | 60 | 5 | 0.5 | 4 | 0.4 | 0.13 | 0.01 | 0.04 | 0.00 | 70 | 9 | 9 | 0.3 |
| JUDITH | 60 | 5 | 0.5 | 4 | 0.6 | 0.13 | 0.01 | 0.04 | 0.01 | 65 | 10 | 8 | 0.6 |
| MT85200 | 60 | 5 | 0.5 | 4 | 0.3 | 0.14 | 0.01 | 0.04 | 0.01 | 71 | 15 | 9 | 0.8 |
| NUSKY | 60 | 5 | 0.4 | 4 | 0.5 | 0.14 | 0.01 | 0.03 | 0.00 | 59 | 13 | 8 | 0.8 |
| MT9513 | 60 | 5 | 0.3 | 4 | 0.5 | 0.14 | 0.01 | 0.04 | 0.01 | 69 | 13 | 8 | 0.5 |
| MT9904 | 60 | 5 | 0.4 | 4 | 0.4 | 0.13 | 0.01 | 0.04 | 0.01 | 72 | 11 | 9 | 0.2 |
| NORRIS | 60 | 5 | 0.3 | 4 | 0.5 | 0.14 | 0.01 | 0.03 | 0.01 | 59 | 9 | 8 | 0.2 |
| YELLOWSTONE | 60 | 5 | 0.4 | 4 | 0.5 | 0.14 | 0.01 | 0.03 | 0.01 | 57 | 12 | 9 | 0.9 |
| MT0495 | 60 | 5 | 0.4 | 4 | 0.5 | 0.12 | 0.02 | 0.04 | 0.01 | 78 | 15 | 8 | 0.3 |
| MTS0531 | 60 | 6 | 0.9 | 5 | 0.8 | 0.13 | 0.01 | 0.04 | 0.01 | 80 | 14 | 11 | 1.5 |
| DECADE | 60 | 6 | 0.4 | 4 | 0.3 | 0.13 | 0.01 | 0.04 | 0.01 | 68 | 16 | 9 | 0.5 |
| MT06103 | 60 | 5 | 0.4 | 4 | 0.5 | 0.14 | 0.01 | 0.04 | 0.01 | 64 | 11 | 8 | 0.5 |
| JUDEE | 60 | 5 | 0.3 | 4 | 0.3 | 0.13 | 0.00 | 0.04 | 0.01 | 63 | 11 | 8 | 0.1 |
| LAKIN | 60 | 5 | 0.4 | 4 | 0.4 | 0.13 | 0.01 | 0.03 | 0.00 | 59 | 10 | 9 | 0.5 |
| STANTON | 60 | 6 | 0.5 | 4 | 0.4 | 0.13 | 0.01 | 0.04 | 0.01 | 67 | 12 | 9 | 0.2 |
| TREGO | 60 | 5 | 0.3 | 4 | 0.4 | 0.14 | 0.01 | 0.04 | 0.00 | 70 | 13 | 9 | 0.7 |
| KARL_92 | 60 | 5 | 0.4 | 4 | 0.5 | 0.13 | 0.01 | 0.03 | 0.00 | 62 | 9 | 9 | 0.5 |
| DODGE | 60 | 5 | 0.3 | 4 | 0.5 | 0.13 | 0.01 | 0.04 | 0.01 | 71 | 21 | 9 | 0.5 |
| NORKAN | 60 | 5 | 0.4 | 4 | 0.5 | 0.13 | 0.01 | 0.04 | 0.01 | 63 | 10 | 9 | 0.6 |
| CHENEY | 60 | 5 | 0.4 | 4 | 0.4 | 0.13 | 0.01 | 0.03 | 0.00 | 61 | 11 | 8 | 0.4 |
| NEWTON | 60 | 5 | 0.4 | 4 | 0.4 | 0.12 | 0.02 | 0.04 | 0.01 | 64 | 11 | 8 | 0.6 |
| LARNED | 60 | 5 | 0.4 | 4 | 0.5 | 0.13 | 0.01 | 0.04 | 0.01 | 67 | 13 | 8 | 0.7 |
| PARKER76 | 60 | 5 | 0.3 | 4 | 0.6 | 0.13 | 0.01 | 0.03 | 0.00 | 59 | 6 | 8 | 0.2 |
| KIRWIN | 60 | 5 | 0.4 | 4 | 0.4 | 0.12 | 0.01 | 0.03 | 0.00 | 60 | 6 | 9 | 0.7 |
| SAGE | 60 | 5 | 0.5 | 4 | 0.6 | 0.12 | 0.02 | 0.03 | 0.00 | 61 | 10 | 9 | 0.5 |
| TRISON | 60 | 5 | 0.4 | 4 | 0.6 | 0.12 | 0.02 | 0.03 | 0.00 | 60 | 13 | 9 | 0.5 |
| EAGLE | 60 | 5 | 0.4 | 4 | 0.6 | 0.12 | 0.02 | 0.03 | 0.00 | 57 | 7 | 8 | 0.5 |
| SHAWNEE | 60 | 5 | 0.4 | 4 | 0.6 | 0.13 | 0.02 | 0.03 | 0.00 | 60 | 11 | 8 | 0.5 |
| PARKER | 60 | 5 | 0.4 | 4 | 0.3 | 0.14 | 0.01 | 0.04 | 0.01 | 65 | 14 | 9 | 1.0 |
| KAW61 | 60 | 5 | 0.4 | 4 | 0.3 | 0.13 | 0.01 | 0.03 | 0.01 | 59 | 8 | 8 | 0.5 |
| TASCOSA | 60 | 7 | 0.8 | 5 | 0.9 | 0.14 | 0.01 | 0.04 | 0.01 | 79 | 16 | 11 | 0.3 |
| BISON | 60 | 5 | 0.3 | 4 | 0.5 | 0.12 | 0.02 | 0.03 | 0.01 | 62 | 14 | 9 | 0.7 |
| KIOWA | 60 | 5 | 0.5 | 4 | 0.2 | 0.13 | 0.02 | 0.03 | 0.01 | 64 | 12 | 8 | 0.5 |
| WICHITA | 60 | 5 | 0.4 | 4 | 0.2 | 0.13 | 0.01 | 0.04 | 0.01 | 64 | 12 | 8 | 0.1 |
| COMANCHE | 60 | 5 | 0.4 | 4 | 0.4 | 0.13 | 0.01 | 0.04 | 0.01 | 58 | 9 | 8 | 0.4 |
| BAKERS_WHITE | 60 | 5 | 0.4 | 4 | 0.5 | 0.13 | 0.01 | 0.03 | 0.00 | 60 | 9 | 8 | 0.5 |
| BURCHETT | 60 | 5 | 0.4 | 4 | 0.6 | 0.13 | 0.01 | 0.03 | 0.00 | 54 | 7 | 8 | 0.4 |
| CUTTER | 60 | 5 | 0.4 | 4 | 0.5 | 0.13 | 0.01 | 0.03 | 0.01 | 58 | 13 | 9 | 0.9 |
| DUMAS | 60 | 5 | 0.3 | 4 | 0.6 | 0.13 | 0.02 | 0.03 | 0.01 | 64 | 10 | 8 | 0.5 |
| HONDO | 60 | 5 | 0.4 | 4 | 0.5 | 0.13 | 0.02 | 0.03 | 0.00 | 56 | 6 | 8 | 1.0 |
| JAGALENE | 60 | 5 | 0.4 | 4 | 0.4 | 0.13 | 0.01 | 0.03 | 0.00 | 62 | 12 | 9 | 0.5 |
| LONGHORN | 60 | 5 | 0.4 | 4 | 0.5 | 0.13 | 0.01 | 0.03 | 0.00 | 62 | 10 | 8 | 0.5 |
| NEOSHO | 60 | 5 | 0.4 | 4 | 0.5 | 0.13 | 0.01 | 0.03 | 0.00 | 54 | 8 | 9 | 0.5 |
| OGALLALA | 60 | 5 | 0.4 | 4 | 0.6 | 0.13 | 0.01 | 0.03 | 0.00 | 57 | 5 | 9 | 0.7 |
| POSTROCK | 60 | 5 | 0.3 | 4 | 0.8 | 0.13 | 0.01 | 0.03 | 0.00 | 53 | 7 | 8 | 0.8 |
| THUNDERBOLT | 60 | 5 | 0.4 | 4 | 0.7 | 0.13 | 0.01 | 0.03 | 0.01 | 55 | 11 | 8 | 0.4 |
| W04-417 | 60 | 5 | 0.4 | 4 | 0.6 | 0.13 | 0.01 | 0.03 | 0.01 | 54 | 8 | 9 | 0.4 |
| NUFRONTIER | 60 | 5 | 0.3 | 4 | 0.6 | 0.12 | 0.02 | 0.03 | 0.00 | 57 | 6 | 8 | 0.3 |
| NUHORIZON | 60 | 5 | 0.5 | 4 | 0.5 | 0.13 | 0.01 | 0.03 | 0.00 | 57 | 9 | 8 | 0.3 |
| ONAGA | 60 | 6 | 0.5 | 5 | 0.7 | 0.15 | 0.01 | 0.04 | 0.01 | 64 | 9 | 11 | 0.9 |
| RONL | 60 | 5 | 0.4 | 4 | 0.5 | 0.13 | 0.01 | 0.03 | 0.01 | 53 | 11 | 8 | 0.7 |
| 2145 | 60 | 5 | 0.4 | 4 | 0.4 | 0.13 | 0.01 | 0.03 | 0.00 | 49 | 9 | 8 | 0.3 |
| HEYNE | 60 | 5 | 0.5 | 4 | 0.2 | 0.13 | 0.01 | 0.03 | 0.01 | 50 | 10 | 8 | 0.4 |
| KS00F5-20-3 | 60 | 5 | 0.4 | 4 | 0.3 | 0.13 | 0.01 | 0.03 | 0.00 | 52 | 8 | 9 | 0.8 |
| OVERLEY | 60 | 5 | 0.4 | 4 | 0.3 | 0.13 | 0.01 | 0.03 | 0.00 | 51 | 8 | 9 | 0.4 |
| FULLER | 60 | 5 | 0.4 | 4 | 0.5 | 0.13 | 0.01 | 0.03 | 0.01 | 50 | 9 | 8 | 0.5 |
| COSSACK | 60 | 5 | 0.4 | 4 | 0.4 | 0.13 | 0.01 | 0.03 | 0.01 | 55 | 11 | 8 | 0.8 |
| ENHANCER | 60 | 5 | 0.4 | 4 | 0.3 | 0.13 | 0.01 | 0.03 | 0.00 | 52 | 6 | 8 | 0.9 |
| SANTA_FE | 60 | 5 | 0.4 | 4 | 0.6 | 0.13 | 0.01 | 0.03 | 0.00 | 54 | 8 | 9 | 0.2 |
| VENANGO | 60 | 5 | 0.4 | 4 | 0.5 | 0.12 | 0.02 | 0.03 | 0.00 | 54 | 8 | 8 | 0.5 |
| WB411W | 60 | 5 | 0.4 | 4 | 0.6 | 0.13 | 0.01 | 0.03 | 0.00 | 56 | 10 | 8 | 0.8 |
| KEOTA | 60 | 5 | 0.5 | 4 | 0.5 | 0.13 | 0.00 | 0.03 | 0.00 | 53 | 5 | 9 | 0.4 |
| TX05A001822 | 60 | 6 | 0.4 | 4 | 0.6 | 0.13 | 0.00 | 0.03 | 0.00 | 52 | 5 | 8 | 0.4 |
| TX06A001263 | 60 | 5 | 0.5 | 4 | 0.5 | 0.13 | 0.00 | 0.03 | 0.00 | 56 | 8 | 8 | 0.7 |
| TX06A001132 | 60 | 5 | 0.4 | 4 | 0.5 | 0.13 | 0.00 | 0.03 | 0.00 | 56 | 8 | 8 | 0.7 |
| TX06A001281 | 60 | 5 | 0.4 | 4 | 0.5 | 0.13 | 0.00 | 0.03 | 0.00 | 56 | 6 | 9 | 0.5 |
| TX06A001386 | 60 | 5 | 0.4 | 4 | 0.4 | 0.13 | 0.00 | 0.03 | 0.00 | 55 | 6 | 9 | 0.8 |
| TX05V7259 | 60 | 5 | 0.4 | 4 | 0.6 | 0.13 | 0.02 | 0.03 | 0.00 | 50 | 9 | 8 | 0.6 |
| TX05V7269 | 60 | 5 | 0.4 | 4 | 0.5 | 0.13 | 0.00 | 0.03 | 0.00 | 55 | 5 | 8 | 0.8 |
| TX05A001188 | 60 | 5 | 0.3 | 4 | 0.5 | 0.13 | 0.00 | 0.03 | 0.00 | 54 | 5 | 8 | 0.3 |
| TX07A001279 | 60 | 5 | 0.4 | 4 | 0.6 | 0.13 | 0.00 | 0.03 | 0.00 | 57 | 8 | 9 | 0.7 |
| TX07A001318 | 60 | 5 | 0.4 | 4 | 0.6 | 0.13 | 0.00 | 0.03 | 0.00 | 58 | 11 | 8 | 0.7 |
| TX07A001420 | 60 | 6 | 0.5 | 4 | 0.5 | 0.13 | 0.01 | 0.03 | 0.01 | 58 | 7 | 8 | 0.9 |
| TX06V7266 | 60 | 5 | 0.5 | 4 | 0.5 | 0.13 | 0.00 | 0.03 | 0.01 | 61 | 8 | 9 | 0.7 |
| OK1067071 | 60 | 5 | 0.4 | 4 | 0.4 | 0.13 | 0.00 | 0.04 | 0.01 | 64 | 12 | 8 | 0.9 |
| OK1067274 | 60 | 5 | 0.4 | 4 | 0.4 | 0.13 | 0.00 | 0.03 | 0.00 | 57 | 7 | 9 | 0.8 |
| OK1068002 | 60 | 5 | 0.4 | 4 | 0.4 | 0.13 | 0.00 | 0.04 | 0.01 | 62 | 10 | 8 | 1.1 |
| OK1068009 | 60 | 6 | 0.5 | 4 | 0.7 | 0.13 | 0.00 | 0.03 | 0.00 | 62 | 10 | 9 | 0.9 |
| OK1068026 | 60 | 5 | 0.4 | 4 | 0.5 | 0.12 | 0.02 | 0.03 | 0.00 | 57 | 9 | 9 | 0.6 |
| OK1068112 | 60 | 5 | 0.4 | 4 | 0.5 | 0.13 | 0.02 | 0.03 | 0.01 | 59 | 10 | 8 | 0.6 |
| OK1070275 | 60 | 5 | 0.3 | 4 | 0.7 | 0.13 | 0.00 | 0.03 | 0.00 | 60 | 9 | 9 | 0.5 |
| OK1070267 | 60 | 6 | 0.5 | 4 | 0.5 | 0.13 | 0.00 | 0.03 | 0.01 | 59 | 6 | 8 | 0.6 |
| OK09634 | 60 | 5 | 0.3 | 4 | 0.5 | 0.13 | 0.00 | 0.03 | 0.00 | 61 | 9 | 9 | 0.8 |
| OK10119 | 60 | 5 | 0.4 | 4 | 0.5 | 0.13 | 0.00 | 0.03 | 0.00 | 59 | 7 | 8 | 0.4 |
| GALLAGHER | 60 | 5 | 0.5 | 4 | 0.5 | 0.13 | 0.01 | 0.03 | 0.01 | 58 | 7 | 9 | 0.9 |
| OK07231 | 60 | 5 | 0.4 | 4 | 0.5 | 0.13 | 0.00 | 0.03 | 0.01 | 64 | 15 | 8 | 0.9 |
| OK07S117 | 60 | 5 | 0.4 | 4 | 0.5 | 0.13 | 0.01 | 0.03 | 0.01 | 60 | 10 | 8 | 0.8 |
| OK08328 | 60 | 5 | 0.4 | 4 | 0.6 | 0.13 | 0.00 | 0.03 | 0.00 | 59 | 7 | 8 | 0.9 |
| BIG_SKY | 60 | 5 | 0.3 | 4 | 0.4 | 0.13 | 0.01 | 0.03 | 0.01 | 62 | 10 | 8 | 0.2 |
| DANBY | 60 | 6 | 0.5 | 4 | 0.6 | 0.13 | 0.01 | 0.03 | 0.01 | 59 | 10 | 9 | 0.5 |
| E2041 | 60 | 5 | 0.4 | 4 | 0.3 | 0.13 | 0.01 | 0.03 | 0.00 | 55 | 7 | 9 | 0.5 |
| DENALI | 60 | 5 | 0.4 | 4 | 0.3 | 0.13 | 0.01 | 0.03 | 0.00 | 55 | 9 | 8 | 0.8 |
| CO050337-2 | 60 | 5 | 0.4 | 4 | 0.3 | 0.13 | 0.00 | 0.03 | 0.00 | 58 | 11 | 8 | 0.5 |
| BYRD | 60 | 5 | 0.4 | 4 | 0.3 | 0.13 | 0.01 | 0.03 | 0.01 | 56 | 8 | 9 | 0.8 |
| CO07W245 | 60 | 5 | 0.4 | 4 | 0.4 | 0.13 | 0.00 | 0.03 | 0.00 | 60 | 10 | 8 | 0.7 |
| MCGILL | 60 | 5 | 0.5 | 4 | 0.5 | 0.13 | 0.01 | 0.03 | 0.00 | 61 | 10 | 9 | 0.5 |
| NE02558 | 60 | 5 | 0.4 | 4 | 0.4 | 0.13 | 0.01 | 0.03 | 0.01 | 62 | 11 | 8 | 0.6 |
| NW03666 | 60 | 5 | 0.4 | 4 | 0.3 | 0.13 | 0.01 | 0.04 | 0.01 | 70 | 17 | 8 | 0.2 |
| NE04490 | 60 | 5 | 0.4 | 4 | 0.4 | 0.13 | 0.01 | 0.03 | 0.01 | 62 | 9 | 8 | 0.5 |
| NE05430 | 60 | 5 | 0.5 | 4 | 0.3 | 0.13 | 0.01 | 0.04 | 0.01 | 62 | 9 | 8 | 0.2 |
| NE05496 | 60 | 5 | 0.4 | 4 | 0.5 | 0.13 | 0.00 | 0.04 | 0.01 | 63 | 7 | 9 | 0.7 |
| NE05548 | 60 | 5 | 0.4 | 4 | 0.5 | 0.13 | 0.00 | 0.04 | 0.01 | 63 | 8 | 8 | 0.3 |
| NE06545 | 60 | 5 | 0.5 | 4 | 0.5 | 0.13 | 0.01 | 0.04 | 0.01 | 62 | 12 | 8 | 1.0 |
| NE06607 | 60 | 5 | 0.5 | 4 | 0.5 | 0.13 | 0.01 | 0.04 | 0.01 | 65 | 10 | 8 | 0.8 |
| ROBIDOUX | 60 | 5 | 0.4 | 4 | 0.5 | 0.13 | 0.01 | 0.04 | 0.01 | 62 | 8 | 8 | 0.2 |
| NI06736 | 60 | 5 | 0.4 | 4 | 0.5 | 0.13 | 0.01 | 0.03 | 0.01 | 59 | 8 | 8 | 0.4 |
| NI06737 | 60 | 5 | 0.5 | 4 | 0.5 | 0.13 | 0.01 | 0.03 | 0.01 | 59 | 10 | 8 | 0.5 |
| NI07703 | 60 | 5 | 0.3 | 4 | 0.5 | 0.13 | 0.01 | 0.03 | 0.01 | 57 | 12 | 8 | 0.7 |
| NI08707 | 60 | 5 | 0.4 | 4 | 0.4 | 0.13 | 0.00 | 0.03 | 0.01 | 61 | 11 | 8 | 0.4 |
| NI08708 | 60 | 5 | 0.4 | 4 | 0.5 | 0.13 | 0.01 | 0.04 | 0.01 | 61 | 9 | 8 | 0.3 |
| EVEREST | 60 | 5 | 0.5 | 4 | 0.5 | 0.13 | 0.01 | 0.04 | 0.01 | 61 | 7 | 8 | 0.8 |
| TRIUMPH64 | 120 | 2 | 0.6 | 2 | 0.4 | 0.08 | 0.01 | 0.02 | 0.01 | 28 | 11 | 3 | 0.5 |
| CHISHOLM | 120 | 3 | 0.5 | 2 | 0.4 | 0.10 | 0.01 | 0.02 | 0.01 | 35 | 16 | 4 | 0.5 |
| CUSTER | 120 | 3 | 0.6 | 2 | 0.5 | 0.09 | 0.01 | 0.02 | 0.01 | 38 | 16 | 2 | 0.4 |
| 2174-05 | 120 | 3 | 0.8 | 2 | 0.7 | 0.10 | 0.02 | 0.02 | 0.01 | 23 | 11 | 1 | 0.2 |
| INTRADA | 120 | 2 | 0.5 | 1 | 0.4 | 0.08 | 0.01 | 0.01 | 0.01 | 26 | 10 | 2 | 0.3 |
| OK101 | 120 | 3 | 0.6 | 2 | 0.3 | 0.10 | 0.01 | 0.02 | 0.01 | 41 | 18 | 2 | 0.4 |
| OK102 | 120 | 3 | 0.5 | 2 | 0.4 | 0.08 | 0.01 | 0.02 | 0.01 | 31 | 15 | 2 | 0.4 |
| ENDURANCE | 120 | 4 | 0.3 | 3 | 0.2 | 0.10 | 0.01 | 0.03 | 0.01 | 44 | 7 | 6 | 0.4 |
| DELIVER | 120 | 3 | 0.6 | 1 | 0.4 | 0.09 | 0.01 | 0.02 | 0.01 | 30 | 16 | 2 | 0.5 |
| OK_BULLET | 120 | 3 | 0.6 | 2 | 0.4 | 0.09 | 0.01 | 0.02 | 0.01 | 33 | 12 | 2 | 0.6 |
| CENTERFIELD | 120 | 3 | 0.6 | 2 | 0.4 | 0.09 | 0.01 | 0.02 | 0.01 | 28 | 13 | 3 | 0.2 |
| GUYMON | 120 | 6 | 1.0 | 4 | 0.6 | 0.11 | 0.01 | 0.03 | 0.01 | 53 | 18 | 8 | 0.4 |
| DUSTER | 120 | 3 | 0.6 | 2 | 0.5 | 0.08 | 0.01 | 0.02 | 0.01 | 31 | 16 | 3 | 0.5 |
| OK_RISING | 120 | 3 | 0.4 | 2 | 0.4 | 0.10 | 0.01 | 0.02 | 0.01 | 30 | 17 | 2 | 0.5 |
| OK02405 | 120 | 2 | 0.5 | 2 | 0.4 | 0.10 | 0.01 | 0.02 | 0.01 | 28 | 15 | 2 | 0.3 |
| PETE | 120 | 3 | 0.6 | 2 | 0.4 | 0.10 | 0.01 | 0.02 | 0.01 | 38 | 17 | 2 | 0.3 |
| BILLINGS | 120 | 2 | 0.5 | 2 | 0.5 | 0.08 | 0.01 | 0.02 | 0.01 | 33 | 15 | 2 | 0.4 |
| OK04505 | 120 | 2 | 0.4 | 1 | 0.5 | 0.10 | 0.01 | 0.01 | 0.01 | 26 | 17 | 2 | 0.5 |
| OK04525 | 120 | 3 | 0.6 | 2 | 0.5 | 0.09 | 0.01 | 0.02 | 0.01 | 29 | 15 | 2 | 0.6 |
| OK04507 | 120 | 3 | 0.3 | 3 | 0.3 | 0.10 | 0.01 | 0.02 | 0.01 | 38 | 13 | 5 | 0.4 |
| OK05830 | 120 | 3 | 0.5 | 2 | 0.5 | 0.09 | 0.01 | 0.02 | 0.01 | 32 | 19 | 2 | 0.3 |
| OK04111 | 120 | 3 | 0.5 | 2 | 0.4 | 0.09 | 0.01 | 0.02 | 0.01 | 31 | 15 | 2 | 0.5 |
| OK04415 | 120 | 3 | 0.5 | 2 | 0.4 | 0.09 | 0.01 | 0.01 | 0.01 | 27 | 14 | 2 | 0.6 |
| OK05711W | 120 | 3 | 0.6 | 2 | 0.2 | 0.10 | 0.01 | 0.02 | 0.01 | 35 | 16 | 3 | 0.2 |
| OK05723W | 120 | 2 | 0.4 | 1 | 0.3 | 0.10 | 0.01 | 0.01 | 0.01 | 26 | 16 | 2 | 0.3 |
| OK05108 | 120 | 3 | 0.6 | 2 | 0.6 | 0.11 | 0.01 | 0.02 | 0.01 | 32 | 9 | 3 | 0.1 |
| OK05122 | 120 | 3 | 0.6 | 2 | 0.4 | 0.09 | 0.01 | 0.02 | 0.01 | 41 | 19 | 2 | 0.4 |
| OK05526 | 120 | 3 | 0.6 | 2 | 0.5 | 0.09 | 0.01 | 0.02 | 0.01 | 28 | 14 | 2 | 0.4 |
| OK05134 | 120 | 3 | 0.6 | 2 | 0.5 | 0.10 | 0.01 | 0.02 | 0.01 | 27 | 15 | 2 | 0.8 |
| OK05303 | 120 | 2 | 0.6 | 2 | 0.5 | 0.09 | 0.01 | 0.02 | 0.01 | 36 | 15 | 2 | 0.5 |
| OK05312 | 120 | 3 | 0.5 | 2 | 0.3 | 0.10 | 0.01 | 0.02 | 0.01 | 30 | 15 | 2 | 0.3 |
| OK05511 | 120 | 3 | 0.6 | 2 | 0.4 | 0.09 | 0.01 | 0.01 | 0.01 | 25 | 14 | 2 | 0.5 |
| OK05204 | 120 | 3 | 0.4 | 2 | 0.4 | 0.10 | 0.01 | 0.02 | 0.01 | 39 | 18 | 2 | 0.5 |
| GARRISON | 120 | 3 | 0.4 | 2 | 0.4 | 0.10 | 0.01 | 0.02 | 0.01 | 35 | 13 | 3 | 0.3 |
| OK06114 | 120 | 3 | 0.5 | 2 | 0.6 | 0.09 | 0.01 | 0.02 | 0.01 | 27 | 13 | 2 | 0.5 |
| OK06210 | 120 | 3 | 0.4 | 2 | 0.3 | 0.10 | 0.01 | 0.02 | 0.01 | 41 | 17 | 4 | 0.4 |
| OK06319 | 120 | 2 | 0.4 | 2 | 0.5 | 0.09 | 0.01 | 0.02 | 0.01 | 33 | 17 | 2 | 0.4 |
| OK06318 | 120 | 2 | 0.4 | 2 | 0.3 | 0.10 | 0.01 | 0.02 | 0.01 | 35 | 17 | 2 | 0.2 |
| OK06336 | 120 | 2 | 0.2 | 2 | 0.5 | 0.10 | 0.01 | 0.02 | 0.01 | 37 | 16 | 2 | 0.3 |
| AGATE | 120 | 2 | 0.3 | 1 | 0.4 | 0.10 | 0.01 | 0.01 | 0.01 | 25 | 14 | 1 | 0.4 |
| ALLIANCE | 120 | 2 | 0.4 | 1 | 0.5 | 0.09 | 0.01 | 0.01 | 0.01 | 26 | 16 | 2 | 0.1 |
| ANTELOPE | 120 | 2 | 0.3 | 1 | 0.5 | 0.08 | 0.01 | 0.01 | 0.01 | 23 | 16 | 2 | 0.5 |
| ARAPAHOE | 120 | 2 | 0.4 | 1 | 0.6 | 0.10 | 0.01 | 0.02 | 0.01 | 29 | 13 | 1 | 0.6 |
| BENNETT | 120 | 3 | 0.6 | 2 | 0.4 | 0.10 | 0.01 | 0.02 | 0.01 | 34 | 15 | 2 | 0.3 |
| BUCKSKIN | 120 | 2 | 0.5 | 2 | 0.5 | 0.09 | 0.01 | 0.02 | 0.01 | 34 | 17 | 2 | 0.2 |
| CENTURK78 | 120 | 3 | 0.6 | 2 | 0.5 | 0.10 | 0.01 | 0.02 | 0.01 | 37 | 16 | 2 | 0.6 |
| CHEYENNE | 120 | 3 | 0.5 | 2 | 0.3 | 0.10 | 0.01 | 0.02 | 0.01 | 37 | 17 | 3 | 0.2 |
| COLT | 120 | 2 | 0.4 | 1 | 0.6 | 0.10 | 0.01 | 0.02 | 0.01 | 29 | 15 | 2 | 0.4 |
| COUGAR | 120 | 2 | 0.5 | 2 | 0.6 | 0.09 | 0.01 | 0.02 | 0.01 | 29 | 15 | 3 | 0.6 |
| CULVER | 120 | 2 | 0.6 | 2 | 0.5 | 0.08 | 0.01 | 0.02 | 0.01 | 32 | 18 | 2 | 0.4 |
| GAGE | 120 | 4 | 0.5 | 3 | 0.8 | 0.10 | 0.01 | 0.03 | 0.01 | 52 | 19 | 7 | 0.5 |
| GOODSTREAK | 120 | 3 | 0.2 | 2 | 0.3 | 0.10 | 0.01 | 0.02 | 0.01 | 36 | 15 | 5 | 0.1 |
| HALLAM | 120 | 3 | 0.3 | 2 | 0.2 | 0.09 | 0.01 | 0.01 | 0.01 | 26 | 14 | 4 | 0.2 |
| HARRY | 120 | 2 | 0.4 | 1 | 0.5 | 0.09 | 0.01 | 0.02 | 0.01 | 28 | 14 | 1 | 0.3 |
| HOMESTEAD | 120 | 3 | 0.5 | 2 | 0.3 | 0.10 | 0.01 | 0.02 | 0.01 | 34 | 16 | 3 | 0.1 |
| INFINITY_CL | 120 | 2 | 0.4 | 2 | 0.4 | 0.09 | 0.01 | 0.02 | 0.01 | 29 | 18 | 2 | 0.5 |
| KHARKOF | 120 | 3 | 0.6 | 2 | 0.3 | 0.10 | 0.01 | 0.02 | 0.01 | 34 | 16 | 3 | 0.3 |
| MILLENNIUM | 120 | 2 | 0.5 | 2 | 0.4 | 0.09 | 0.01 | 0.02 | 0.01 | 32 | 15 | 2 | 0.4 |
| CAMELOT | 120 | 2 | 0.4 | 1 | 0.4 | 0.09 | 0.01 | 0.02 | 0.01 | 27 | 13 | 2 | 0.5 |
| OVERLAND | 120 | 3 | 0.6 | 2 | 0.4 | 0.09 | 0.01 | 0.02 | 0.01 | 34 | 17 | 2 | 0.5 |
| NE99495 | 120 | 3 | 0.3 | 2 | 0.2 | 0.10 | 0.01 | 0.02 | 0.01 | 33 | 14 | 5 | 0.3 |
| NIOBRARA | 120 | 4 | 0.4 | 3 | 0.2 | 0.10 | 0.01 | 0.02 | 0.01 | 38 | 18 | 5 | 0.5 |
| NUPLAINS | 120 | 2 | 0.5 | 2 | 0.5 | 0.09 | 0.01 | 0.02 | 0.01 | 26 | 16 | 3 | 0.4 |
| PRONGHORN | 120 | 2 | 0.3 | 2 | 0.5 | 0.09 | 0.01 | 0.02 | 0.01 | 28 | 16 | 2 | 0.2 |
| RAWHIDE | 120 | 2 | 0.4 | 1 | 0.6 | 0.09 | 0.02 | 0.02 | 0.01 | 35 | 19 | 2 | 0.3 |
| REDLAND | 120 | 2 | 0.5 | 2 | 0.5 | 0.09 | 0.01 | 0.02 | 0.01 | 30 | 15 | 2 | 0.4 |
| SCOUT66 | 120 | 3 | 0.5 | 2 | 0.3 | 0.10 | 0.01 | 0.02 | 0.01 | 36 | 18 | 3 | 0.2 |
| SIOUXLAND | 120 | 2 | 0.5 | 1 | 0.5 | 0.09 | 0.01 | 0.02 | 0.01 | 32 | 13 | 2 | 0.3 |
| TURKEY_NEBSEL | 120 | 2 | 0.4 | 2 | 0.6 | 0.09 | 0.01 | 0.02 | 0.01 | 32 | 18 | 3 | 0.4 |
| VISTA | 120 | 2 | 0.5 | 1 | 0.5 | 0.09 | 0.01 | 0.02 | 0.01 | 34 | 19 | 2 | 0.5 |
| WAHOO | 120 | 2 | 0.5 | 1 | 0.6 | 0.09 | 0.01 | 0.01 | 0.01 | 25 | 16 | 2 | 0.4 |
| WARRIOR | 120 | 3 | 0.6 | 2 | 0.6 | 0.09 | 0.01 | 0.02 | 0.01 | 35 | 13 | 3 | 0.3 |
| WESLEY | 120 | 3 | 0.2 | 2 | 0.5 | 0.10 | 0.01 | 0.02 | 0.01 | 38 | 18 | 5 | 0.5 |
| WICHITA | 120 | 3 | 0.6 | 2 | 0.6 | 0.09 | 0.01 | 0.02 | 0.01 | 38 | 16 | 3 | 0.3 |
| WINDSTAR | 120 | 2 | 0.5 | 2 | 0.6 | 0.09 | 0.01 | 0.02 | 0.01 | 30 | 19 | 2 | 0.6 |
| LANCER | 120 | 2 | 0.6 | 2 | 0.7 | 0.10 | 0.01 | 0.02 | 0.01 | 33 | 15 | 3 | 0.3 |
| ANTON | 120 | 2 | 0.5 | 1 | 0.6 | 0.09 | 0.01 | 0.02 | 0.01 | 34 | 15 | 2 | 0.4 |
| MACE | 120 | 2 | 0.4 | 1 | 0.5 | 0.08 | 0.01 | 0.01 | 0.01 | 25 | 18 | 2 | 0.5 |
| TAM107-R7 | 120 | 2 | 0.5 | 2 | 0.5 | 0.09 | 0.01 | 0.01 | 0.01 | 23 | 16 | 2 | 0.3 |
| ARLIN | 120 | 3 | 0.7 | 2 | 0.4 | 0.10 | 0.01 | 0.02 | 0.01 | 40 | 19 | 2 | 0.3 |
| ALICE | 120 | 2 | 0.4 | 2 | 0.5 | 0.09 | 0.01 | 0.02 | 0.01 | 28 | 12 | 2 | 0.5 |
| DARRELL | 120 | 2 | 0.4 | 2 | 0.7 | 0.09 | 0.01 | 0.02 | 0.01 | 32 | 12 | 3 | 0.5 |
| EXPEDITION | 120 | 2 | 0.4 | 2 | 0.5 | 0.09 | 0.01 | 0.02 | 0.01 | 30 | 18 | 3 | 0.3 |
| WENDY | 120 | 3 | 0.3 | 2 | 0.3 | 0.11 | 0.01 | 0.02 | 0.01 | 41 | 13 | 5 | 0.5 |
| SD00111-9 | 120 | 2 | 0.4 | 2 | 0.5 | 0.09 | 0.01 | 0.02 | 0.01 | 30 | 12 | 2 | 0.5 |
| SD01237 | 120 | 2 | 0.4 | 2 | 0.6 | 0.09 | 0.01 | 0.02 | 0.01 | 32 | 15 | 3 | 0.4 |
| SD01058 | 120 | 2 | 0.4 | 1 | 0.4 | 0.09 | 0.01 | 0.02 | 0.01 | 32 | 13 | 2 | 0.2 |
| SD05118 | 120 | 3 | 0.7 | 2 | 0.6 | 0.10 | 0.01 | 0.02 | 0.01 | 37 | 18 | 3 | 0.3 |
| SD05210 | 120 | 2 | 0.5 | 1 | 0.4 | 0.09 | 0.01 | 0.01 | 0.01 | 27 | 17 | 2 | 0.3 |
| SD05W018 | 120 | 2 | 0.4 | 2 | 0.5 | 0.09 | 0.01 | 0.02 | 0.01 | 27 | 14 | 3 | 0.6 |
| NEKOTA | 120 | 3 | 0.6 | 2 | 0.3 | 0.10 | 0.01 | 0.02 | 0.01 | 38 | 16 | 3 | 0.2 |
| TANDEM | 120 | 3 | 0.6 | 2 | 0.4 | 0.10 | 0.01 | 0.02 | 0.01 | 35 | 17 | 3 | 0.2 |
| CRIMSON | 120 | 2 | 0.4 | 1 | 0.6 | 0.09 | 0.01 | 0.02 | 0.01 | 31 | 15 | 2 | 0.4 |
| ROSE | 120 | 2 | 0.3 | 1 | 0.5 | 0.09 | 0.01 | 0.02 | 0.01 | 27 | 16 | 1 | 0.2 |
| DAWN | 120 | 3 | 0.4 | 2 | 0.5 | 0.10 | 0.01 | 0.02 | 0.01 | 35 | 14 | 3 | 0.4 |
| WINOKA | 120 | 2 | 0.5 | 2 | 0.5 | 0.09 | 0.01 | 0.02 | 0.01 | 31 | 20 | 1 | 0.6 |
| NELL | 120 | 3 | 0.5 | 2 | 0.5 | 0.10 | 0.01 | 0.02 | 0.01 | 36 | 16 | 3 | 0.5 |
| RITA | 120 | 2 | 0.4 | 2 | 0.5 | 0.09 | 0.01 | 0.02 | 0.01 | 30 | 16 | 3 | 0.3 |
| BRONZE | 120 | 2 | 0.5 | 1 | 0.4 | 0.08 | 0.01 | 0.02 | 0.01 | 28 | 15 | 2 | 0.3 |
| HUME | 120 | 3 | 0.5 | 2 | 0.4 | 0.09 | 0.01 | 0.02 | 0.01 | 31 | 14 | 2 | 0.4 |
| GENT | 120 | 2 | 0.4 | 2 | 0.6 | 0.09 | 0.01 | 0.02 | 0.01 | 28 | 15 | 2 | 0.5 |
| HARDING | 120 | 2 | 0.6 | 1 | 0.5 | 0.09 | 0.01 | 0.02 | 0.01 | 32 | 16 | 2 | 0.4 |
| HV9W03-1551WP | 120 | 2 | 0.6 | 2 | 0.3 | 0.10 | 0.01 | 0.02 | 0.01 | 33 | 17 | 2 | 0.2 |
| G1878 | 120 | 2 | 0.5 | 2 | 0.6 | 0.10 | 0.01 | 0.02 | 0.01 | 34 | 16 | 2 | 0.3 |
| HV9W03-1379R | 120 | 2 | 0.3 | 1 | 0.5 | 0.09 | 0.01 | 0.01 | 0.01 | 26 | 16 | 2 | 0.3 |
| HV9W03-1596R | 120 | 2 | 0.4 | 2 | 0.6 | 0.08 | 0.01 | 0.02 | 0.01 | 28 | 16 | 3 | 0.4 |
| HV9W05-1280R | 120 | 2 | 0.6 | 2 | 0.7 | 0.09 | 0.01 | 0.02 | 0.01 | 33 | 19 | 2 | 0.6 |
| HV9W06-504 | 120 | 2 | 0.5 | 2 | 0.5 | 0.09 | 0.01 | 0.02 | 0.01 | 30 | 16 | 3 | 0.3 |
| SPARTAN | 120 | 2 | 0.5 | 1 | 0.6 | 0.09 | 0.01 | 0.02 | 0.01 | 28 | 16 | 2 | 0.4 |
| HV906-865 | 120 | 2 | 0.6 | 2 | 0.6 | 0.10 | 0.01 | 0.02 | 0.01 | 36 | 16 | 3 | 0.4 |
| TARKIO | 120 | 2 | 0.5 | 1 | 0.6 | 0.09 | 0.01 | 0.02 | 0.01 | 32 | 18 | 2 | 0.3 |
| SMOKYHILL | 120 | 2 | 0.4 | 1 | 0.5 | 0.09 | 0.01 | 0.02 | 0.01 | 33 | 17 | 2 | 0.5 |
| SHOCKER | 120 | 2 | 0.4 | 1 | 0.5 | 0.10 | 0.01 | 0.02 | 0.01 | 27 | 16 | 2 | 0.4 |
| VONA | 120 | 2 | 0.4 | 2 | 0.5 | 0.08 | 0.01 | 0.02 | 0.01 | 31 | 16 | 2 | 0.4 |
| CO940610 | 120 | 2 | 0.4 | 1 | 0.5 | 0.09 | 0.01 | 0.02 | 0.01 | 32 | 16 | 2 | 0.3 |
| AVALANCHE | 120 | 3 | 0.9 | 2 | 0.8 | 0.10 | 0.01 | 0.02 | 0.01 | 40 | 17 | 3 | 0.4 |
| BOND_CL | 120 | 2 | 0.5 | 2 | 0.5 | 0.09 | 0.01 | 0.02 | 0.01 | 29 | 16 | 2 | 0.5 |
| PLATTE | 120 | 2 | 0.3 | 1 | 0.6 | 0.09 | 0.01 | 0.02 | 0.01 | 32 | 14 | 2 | 0.4 |
| LINDON | 120 | 2 | 0.4 | 1 | 0.5 | 0.08 | 0.01 | 0.02 | 0.01 | 32 | 18 | 2 | 0.2 |
| CO03W043 | 120 | 2 | 0.5 | 1 | 0.6 | 0.10 | 0.01 | 0.02 | 0.01 | 29 | 12 | 3 | 0.4 |
| SNOWMASS | 120 | 2 | 0.5 | 2 | 0.5 | 0.09 | 0.01 | 0.02 | 0.01 | 28 | 11 | 3 | 0.8 |
| THUNDER_CL | 120 | 3 | 0.5 | 2 | 0.4 | 0.10 | 0.01 | 0.02 | 0.01 | 33 | 15 | 3 | 0.4 |
| CO04025 | 120 | 2 | 0.4 | 2 | 0.6 | 0.09 | 0.01 | 0.02 | 0.01 | 30 | 16 | 3 | 0.5 |
| CO04393 | 120 | 3 | 0.4 | 2 | 0.5 | 0.10 | 0.01 | 0.02 | 0.01 | 37 | 16 | 4 | 0.5 |
| CO04499 | 120 | 2 | 0.5 | 2 | 0.6 | 0.09 | 0.01 | 0.01 | 0.01 | 25 | 17 | 3 | 0.9 |
| CO04W320 | 120 | 4 | 0.7 | 2 | 0.8 | 0.11 | 0.01 | 0.02 | 0.01 | 28 | 11 | 2 | 0.5 |
| LAMAR | 120 | 3 | 0.5 | 2 | 0.5 | 0.10 | 0.01 | 0.02 | 0.01 | 35 | 18 | 3 | 0.4 |
| CARSON | 120 | 4 | 0.7 | 2 | 0.9 | 0.10 | 0.02 | 0.02 | 0.01 | 33 | 10 | 2 | 0.3 |
| HAIL | 120 | 2 | 0.7 | 2 | 0.5 | 0.10 | 0.01 | 0.02 | 0.01 | 28 | 13 | 1 | 0.2 |
| SANDY | 120 | 2 | 0.5 | 2 | 0.5 | 0.10 | 0.01 | 0.02 | 0.01 | 30 | 16 | 2 | 0.4 |
| DUKE | 120 | 2 | 0.6 | 1 | 0.6 | 0.09 | 0.01 | 0.02 | 0.01 | 31 | 14 | 2 | 0.3 |
| HALT | 120 | 3 | 0.5 | 2 | 0.5 | 0.10 | 0.01 | 0.02 | 0.01 | 33 | 16 | 3 | 0.1 |
| HATCHER | 120 | 2 | 0.5 | 2 | 0.4 | 0.09 | 0.01 | 0.01 | 0.01 | 27 | 15 | 1 | 0.4 |
| PRAIRIE_RED | 120 | 3 | 0.6 | 2 | 0.5 | 0.10 | 0.01 | 0.02 | 0.01 | 33 | 17 | 3 | 0.7 |
| ABOVE | 120 | 2 | 0.4 | 2 | 0.5 | 0.10 | 0.01 | 0.02 | 0.01 | 30 | 16 | 3 | 0.5 |
| CO03064 | 120 | 2 | 0.4 | 2 | 0.5 | 0.09 | 0.01 | 0.02 | 0.01 | 33 | 18 | 2 | 0.4 |
| BILL_BROWN | 120 | 2 | 0.5 | 1 | 0.6 | 0.09 | 0.01 | 0.02 | 0.01 | 27 | 16 | 1 | 0.2 |
| RIPPER | 120 | 2 | 0.3 | 1 | 0.4 | 0.09 | 0.01 | 0.02 | 0.01 | 30 | 12 | 1 | 0.2 |
| PROWERS | 120 | 3 | 0.5 | 2 | 0.6 | 0.10 | 0.01 | 0.02 | 0.01 | 34 | 17 | 4 | 0.4 |
| AKRON | 120 | 2 | 0.5 | 2 | 0.4 | 0.10 | 0.01 | 0.02 | 0.01 | 35 | 17 | 2 | 0.2 |
| JULES | 120 | 3 | 0.6 | 2 | 0.5 | 0.10 | 0.01 | 0.02 | 0.01 | 33 | 17 | 3 | 0.3 |
| YUMA | 120 | 2 | 0.3 | 1 | 0.4 | 0.09 | 0.01 | 0.02 | 0.01 | 29 | 18 | 2 | 0.4 |
| TAMW-101 | 120 | 2 | 0.4 | 1 | 0.5 | 0.10 | 0.01 | 0.02 | 0.01 | 34 | 17 | 3 | 0.3 |
| TAM105 | 120 | 2 | 0.4 | 1 | 0.5 | 0.09 | 0.01 | 0.02 | 0.01 | 32 | 18 | 2 | 0.4 |
| TAM107 | 120 | 2 | 0.4 | 1 | 0.4 | 0.08 | 0.01 | 0.02 | 0.01 | 30 | 14 | 2 | 0.6 |
| TAM109 | 120 | 2 | 0.4 | 1 | 0.4 | 0.08 | 0.01 | 0.02 | 0.01 | 39 | 21 | 3 | 0.6 |
| TAM110 | 120 | 3 | 0.6 | 2 | 0.4 | 0.10 | 0.01 | 0.02 | 0.01 | 40 | 18 | 3 | 0.2 |
| TAM111 | 120 | 3 | 0.6 | 2 | 0.4 | 0.10 | 0.01 | 0.02 | 0.01 | 31 | 15 | 3 | 0.4 |
| TAM112 | 120 | 3 | 0.5 | 2 | 0.5 | 0.09 | 0.01 | 0.02 | 0.01 | 36 | 18 | 3 | 0.2 |
| TAM200 | 120 | 3 | 0.3 | 2 | 0.3 | 0.09 | 0.01 | 0.02 | 0.01 | 31 | 15 | 3 | 0.2 |
| TAM202 | 120 | 2 | 0.4 | 2 | 0.4 | 0.09 | 0.01 | 0.02 | 0.01 | 29 | 11 | 1 | 0.2 |
| TAM203 | 120 | 2 | 0.4 | 2 | 0.6 | 0.09 | 0.01 | 0.02 | 0.01 | 29 | 15 | 2 | 0.4 |
| TAM302 | 120 | 3 | 0.2 | 3 | 0.3 | 0.10 | 0.01 | 0.02 | 0.01 | 35 | 14 | 5 | 0.5 |
| TAM303 | 120 | 2 | 0.4 | 1 | 0.5 | 0.09 | 0.01 | 0.02 | 0.01 | 33 | 19 | 1 | 0.3 |
| TAM304 | 120 | 2 | 0.3 | 1 | 0.3 | 0.09 | 0.01 | 0.01 | 0.01 | 27 | 16 | 2 | 0.2 |
| TAM400 | 120 | 2 | 0.4 | 2 | 0.5 | 0.09 | 0.01 | 0.02 | 0.01 | 31 | 14 | 1 | 0.3 |
| LOCKETT | 120 | 3 | 0.5 | 2 | 0.1 | 0.10 | 0.01 | 0.02 | 0.01 | 39 | 15 | 3 | 0.2 |
| STURDY | 120 | 2 | 0.3 | 1 | 0.4 | 0.09 | 0.01 | 0.02 | 0.01 | 34 | 16 | 1 | 0.1 |
| STURDY_2K | 120 | 2 | 0.4 | 1 | 0.5 | 0.08 | 0.01 | 0.02 | 0.01 | 31 | 17 | 2 | 0.3 |
| MIT | 120 | 2 | 0.4 | 1 | 0.5 | 0.08 | 0.01 | 0.01 | 0.01 | 28 | 15 | 2 | 0.3 |
| CAPROCK | 120 | 3 | 0.6 | 2 | 0.4 | 0.10 | 0.01 | 0.02 | 0.01 | 35 | 15 | 3 | 0.4 |
| TX01A5936 | 120 | 2 | 0.2 | 1 | 0.3 | 0.08 | 0.00 | 0.02 | 0.01 | 33 | 17 | 2 | 0.3 |
| TAM401 | 120 | 2 | 0.3 | 2 | 0.4 | 0.10 | 0.01 | 0.02 | 0.01 | 32 | 17 | 3 | 0.5 |
| TX02A0252 | 120 | 2 | 0.4 | 1 | 0.5 | 0.09 | 0.01 | 0.01 | 0.01 | 29 | 17 | 1 | 0.4 |
| TX03A0148 | 120 | 2 | 0.4 | 1 | 0.5 | 0.08 | 0.01 | 0.01 | 0.01 | 28 | 16 | 1 | 0.3 |
| TX03A0563 | 120 | 2 | 0.4 | 1 | 0.5 | 0.09 | 0.01 | 0.02 | 0.01 | 31 | 17 | 1 | 0.3 |
| TX04A001246 | 120 | 2 | 0.2 | 1 | 0.5 | 0.09 | 0.01 | 0.02 | 0.01 | 31 | 22 | 2 | 0.4 |
| TX01V5134RC-3 | 120 | 3 | 0.2 | 2 | 0.5 | 0.10 | 0.01 | 0.02 | 0.01 | 39 | 14 | 5 | 0.2 |
| TX04M410164 | 120 | 2 | 0.2 | 1 | 0.3 | 0.09 | 0.01 | 0.01 | 0.01 | 24 | 14 | 2 | 0.3 |
| TX04M410211 | 120 | 3 | 0.7 | 2 | 0.7 | 0.10 | 0.01 | 0.02 | 0.01 | 29 | 15 | 2 | 0.3 |
| TX04V075080 | 120 | 2 | 0.4 | 1 | 0.5 | 0.09 | 0.01 | 0.02 | 0.01 | 32 | 13 | 1 | 0.4 |
| TX99A0153-1 | 120 | 2 | 0.4 | 1 | 0.5 | 0.08 | 0.01 | 0.01 | 0.01 | 27 | 17 | 2 | 0.2 |
| TX01M5009-28 | 120 | 2 | 0.5 | 2 | 0.4 | 0.10 | 0.01 | 0.02 | 0.01 | 35 | 12 | 2 | 0.3 |
| TX00V1131 | 120 | 2 | 0.6 | 1 | 0.5 | 0.08 | 0.01 | 0.02 | 0.01 | 38 | 20 | 2 | 0.3 |
| TX99U8618 | 120 | 3 | 0.1 | 2 | 0.3 | 0.10 | 0.01 | 0.02 | 0.01 | 34 | 16 | 4 | 0.4 |
| TX96D1073 | 120 | 2 | 0.4 | 1 | 0.5 | 0.08 | 0.01 | 0.01 | 0.01 | 26 | 14 | 2 | 0.1 |
| 2180 | 120 | 2 | 0.3 | 1 | 0.5 | 0.09 | 0.01 | 0.02 | 0.01 | 30 | 16 | 2 | 0.2 |
| HG-9 | 120 | 2 | 0.4 | 2 | 0.5 | 0.09 | 0.01 | 0.01 | 0.01 | 27 | 15 | 1 | 0.4 |
| TX86A5606 | 120 | 3 | 0.7 | 2 | 0.7 | 0.11 | 0.01 | 0.03 | 0.01 | 40 | 11 | 3 | 0.5 |
| TX86A8072 | 120 | 3 | 0.5 | 2 | 0.3 | 0.10 | 0.01 | 0.02 | 0.01 | 38 | 15 | 3 | 0.5 |
| CREST | 120 | 3 | 0.5 | 2 | 0.2 | 0.09 | 0.01 | 0.02 | 0.01 | 30 | 16 | 2 | 0.2 |
| ROSEBUD | 120 | 3 | 0.5 | 2 | 0.3 | 0.10 | 0.01 | 0.02 | 0.01 | 37 | 19 | 3 | 0.3 |
| JUDITH | 120 | 2 | 0.4 | 1 | 0.5 | 0.08 | 0.01 | 0.01 | 0.01 | 28 | 16 | 2 | 0.3 |
| MT85200 | 120 | 3 | 0.5 | 2 | 0.6 | 0.10 | 0.01 | 0.02 | 0.01 | 36 | 18 | 3 | 0.3 |
| NUSKY | 120 | 2 | 0.5 | 1 | 0.5 | 0.08 | 0.01 | 0.02 | 0.01 | 30 | 19 | 1 | 0.4 |
| MT9513 | 120 | 2 | 0.6 | 2 | 0.5 | 0.10 | 0.01 | 0.02 | 0.01 | 37 | 16 | 3 | 0.3 |
| MT9904 | 120 | 3 | 0.7 | 2 | 0.5 | 0.10 | 0.01 | 0.02 | 0.01 | 35 | 14 | 3 | 0.7 |
| NORRIS | 120 | 3 | 0.6 | 2 | 0.5 | 0.10 | 0.01 | 0.02 | 0.01 | 32 | 14 | 3 | 0.4 |
| YELLOWSTONE | 120 | 2 | 0.5 | 1 | 0.5 | 0.10 | 0.01 | 0.02 | 0.01 | 32 | 14 | 2 | 0.3 |
| MT0495 | 120 | 2 | 0.3 | 1 | 0.4 | 0.08 | 0.01 | 0.02 | 0.01 | 30 | 18 | 1 | 0.3 |
| MTS0531 | 120 | 4 | 0.7 | 4 | 0.7 | 0.10 | 0.01 | 0.03 | 0.01 | 50 | 19 | 7 | 0.6 |
| DECADE | 120 | 2 | 0.4 | 1 | 0.5 | 0.10 | 0.01 | 0.02 | 0.01 | 32 | 18 | 3 | 0.5 |
| MT06103 | 120 | 2 | 0.2 | 1 | 0.4 | 0.10 | 0.01 | 0.02 | 0.01 | 33 | 18 | 2 | 0.3 |
| JUDEE | 120 | 2 | 0.4 | 2 | 0.5 | 0.09 | 0.01 | 0.02 | 0.01 | 30 | 17 | 2 | 0.3 |
| LAKIN | 120 | 2 | 0.3 | 1 | 0.4 | 0.10 | 0.01 | 0.02 | 0.01 | 34 | 19 | 2 | 0.5 |
| STANTON | 120 | 2 | 0.3 | 1 | 0.5 | 0.09 | 0.01 | 0.02 | 0.01 | 31 | 18 | 2 | 0.3 |
| TREGO | 120 | 2 | 0.1 | 1 | 0.3 | 0.10 | 0.01 | 0.02 | 0.01 | 32 | 19 | 2 | 0.0 |
| KARL_92 | 120 | 2 | 0.4 | 1 | 0.4 | 0.09 | 0.01 | 0.02 | 0.01 | 32 | 17 | 3 | 0.3 |
| DODGE | 120 | 2 | 0.4 | 2 | 0.4 | 0.09 | 0.01 | 0.02 | 0.01 | 32 | 18 | 3 | 0.4 |
| NORKAN | 120 | 2 | 0.2 | 1 | 0.3 | 0.08 | 0.01 | 0.01 | 0.01 | 28 | 18 | 2 | 0.1 |
| CHENEY | 120 | 2 | 0.4 | 1 | 0.4 | 0.08 | 0.01 | 0.02 | 0.01 | 30 | 16 | 1 | 0.3 |
| NEWTON | 120 | 2 | 0.2 | 1 | 0.4 | 0.08 | 0.01 | 0.01 | 0.01 | 28 | 15 | 2 | 0.4 |
| LARNED | 120 | 2 | 0.5 | 2 | 0.4 | 0.10 | 0.01 | 0.02 | 0.01 | 32 | 15 | 3 | 0.2 |
| PARKER76 | 120 | 2 | 0.2 | 1 | 0.4 | 0.09 | 0.01 | 0.02 | 0.01 | 32 | 14 | 2 | 0.3 |
| KIRWIN | 120 | 2 | 0.5 | 2 | 0.5 | 0.10 | 0.01 | 0.02 | 0.01 | 30 | 16 | 3 | 0.4 |
| SAGE | 120 | 2 | 0.4 | 1 | 0.5 | 0.08 | 0.01 | 0.01 | 0.01 | 28 | 18 | 2 | 0.3 |
| TRISON | 120 | 2 | 0.3 | 1 | 0.5 | 0.08 | 0.01 | 0.01 | 0.01 | 30 | 18 | 2 | 0.2 |
| EAGLE | 120 | 2 | 0.3 | 1 | 0.3 | 0.08 | 0.01 | 0.02 | 0.01 | 29 | 18 | 2 | 0.1 |
| SHAWNEE | 120 | 2 | 0.1 | 1 | 0.4 | 0.08 | 0.01 | 0.01 | 0.01 | 27 | 18 | 2 | 0.1 |
| PARKER | 120 | 2 | 0.4 | 2 | 0.6 | 0.10 | 0.01 | 0.02 | 0.01 | 36 | 16 | 3 | 0.5 |
| KAW61 | 120 | 2 | 0.4 | 2 | 0.5 | 0.10 | 0.01 | 0.02 | 0.01 | 29 | 12 | 3 | 0.4 |
| TASCOSA | 120 | 4 | 0.5 | 3 | 0.5 | 0.11 | 0.01 | 0.03 | 0.01 | 51 | 14 | 7 | 0.1 |
| BISON | 120 | 2 | 0.6 | 1 | 0.5 | 0.08 | 0.01 | 0.02 | 0.01 | 30 | 19 | 3 | 0.3 |
| KIOWA | 120 | 2 | 0.2 | 1 | 0.2 | 0.09 | 0.01 | 0.01 | 0.01 | 28 | 17 | 2 | 0.2 |
| WICHITA | 120 | 2 | 0.2 | 1 | 0.4 | 0.09 | 0.01 | 0.02 | 0.01 | 29 | 19 | 2 | 0.2 |
| COMANCHE | 120 | 2 | 0.5 | 2 | 0.5 | 0.10 | 0.01 | 0.02 | 0.01 | 34 | 16 | 3 | 0.6 |
| BAKERS_WHITE | 120 | 2 | 0.3 | 1 | 0.4 | 0.09 | 0.01 | 0.02 | 0.01 | 29 | 16 | 2 | 0.1 |
| BURCHETT | 120 | 2 | 0.5 | 1 | 0.4 | 0.08 | 0.01 | 0.01 | 0.01 | 25 | 15 | 1 | 0.3 |
| CUTTER | 120 | 3 | 0.5 | 2 | 0.5 | 0.10 | 0.01 | 0.02 | 0.01 | 33 | 17 | 3 | 0.3 |
| DUMAS | 120 | 2 | 0.3 | 1 | 0.4 | 0.08 | 0.01 | 0.01 | 0.01 | 28 | 15 | 2 | 0.2 |
| HONDO | 120 | 2 | 0.1 | 1 | 0.2 | 0.08 | 0.01 | 0.01 | 0.01 | 27 | 16 | 1 | 0.1 |
| JAGALENE | 120 | 2 | 0.4 | 2 | 0.4 | 0.09 | 0.01 | 0.02 | 0.01 | 30 | 18 | 3 | 0.3 |
| LONGHORN | 120 | 2 | 0.1 | 1 | 0.2 | 0.08 | 0.01 | 0.01 | 0.01 | 28 | 13 | 2 | 0.1 |
| NEOSHO | 120 | 2 | 0.5 | 2 | 0.4 | 0.10 | 0.01 | 0.02 | 0.01 | 26 | 14 | 3 | 0.4 |
| OGALLALA | 120 | 2 | 0.2 | 1 | 0.4 | 0.08 | 0.01 | 0.01 | 0.01 | 28 | 16 | 2 | 0.4 |
| POSTROCK | 120 | 3 | 0.5 | 2 | 0.5 | 0.09 | 0.02 | 0.02 | 0.01 | 39 | 23 | 2 | 0.4 |
| THUNDERBOLT | 120 | 2 | 0.4 | 2 | 0.4 | 0.10 | 0.01 | 0.02 | 0.01 | 35 | 16 | 3 | 0.3 |
| W04-417 | 120 | 2 | 0.2 | 1 | 0.3 | 0.09 | 0.01 | 0.02 | 0.01 | 29 | 16 | 1 | 0.2 |
| NUFRONTIER | 120 | 3 | 0.5 | 2 | 0.6 | 0.08 | 0.01 | 0.02 | 0.01 | 31 | 17 | 3 | 0.3 |
| NUHORIZON | 120 | 2 | 0.2 | 1 | 0.3 | 0.10 | 0.01 | 0.02 | 0.01 | 29 | 17 | 2 | 0.2 |
| ONAGA | 120 | 4 | 0.7 | 2 | 0.5 | 0.12 | 0.02 | 0.02 | 0.01 | 37 | 11 | 4 | 0.5 |
| RONL | 120 | 2 | 0.2 | 1 | 0.3 | 0.09 | 0.01 | 0.02 | 0.01 | 29 | 17 | 2 | 0.1 |
| 2145 | 120 | 2 | 0.2 | 1 | 0.2 | 0.09 | 0.01 | 0.01 | 0.01 | 26 | 16 | 1 | 0.0 |
| HEYNE | 120 | 2 | 0.2 | 1 | 0.2 | 0.09 | 0.01 | 0.01 | 0.01 | 27 | 15 | 2 | 0.1 |
| KS00F5-20-3 | 120 | 2 | 0.3 | 1 | 0.3 | 0.08 | 0.01 | 0.01 | 0.01 | 25 | 14 | 1 | 0.2 |
| OVERLEY | 120 | 3 | 0.7 | 1 | 0.6 | 0.09 | 0.01 | 0.02 | 0.01 | 33 | 19 | 2 | 0.4 |
| FULLER | 120 | 3 | 0.6 | 2 | 0.4 | 0.10 | 0.01 | 0.02 | 0.01 | 30 | 13 | 3 | 0.5 |
| COSSACK | 120 | 3 | 0.6 | 2 | 0.5 | 0.10 | 0.01 | 0.02 | 0.01 | 33 | 15 | 3 | 0.4 |
| ENHANCER | 120 | 3 | 0.3 | 2 | 0.2 | 0.10 | 0.01 | 0.02 | 0.01 | 31 | 16 | 4 | 0.4 |
| SANTA_FE | 120 | 2 | 0.2 | 1 | 0.3 | 0.10 | 0.01 | 0.01 | 0.01 | 28 | 14 | 2 | 0.2 |
| VENANGO | 120 | 2 | 0.3 | 1 | 0.3 | 0.08 | 0.01 | 0.01 | 0.01 | 28 | 18 | 2 | 0.1 |
| WB411W | 120 | 2 | 0.4 | 2 | 0.4 | 0.10 | 0.01 | 0.02 | 0.01 | 30 | 11 | 3 | 0.4 |
| KEOTA | 120 | 2 | 0.4 | 1 | 0.5 | 0.09 | 0.01 | 0.01 | 0.01 | 25 | 14 | 2 | 0.1 |
| TX05A001822 | 120 | 2 | 0.2 | 1 | 0.3 | 0.08 | 0.01 | 0.01 | 0.01 | 25 | 17 | 1 | 0.1 |
| TX06A001263 | 120 | 2 | 0.3 | 1 | 0.3 | 0.08 | 0.01 | 0.01 | 0.01 | 27 | 18 | 2 | 0.1 |
| TX06A001132 | 120 | 2 | 0.3 | 1 | 0.3 | 0.08 | 0.01 | 0.01 | 0.01 | 27 | 18 | 2 | 0.3 |
| TX06A001281 | 120 | 2 | 0.7 | 1 | 0.5 | 0.10 | 0.01 | 0.02 | 0.01 | 38 | 22 | 3 | 0.4 |
| TX06A001386 | 120 | 2 | 0.3 | 1 | 0.4 | 0.09 | 0.01 | 0.02 | 0.01 | 28 | 14 | 2 | 0.2 |
| TX05V7259 | 120 | 2 | 0.2 | 1 | 0.5 | 0.09 | 0.01 | 0.02 | 0.01 | 30 | 15 | 2 | 0.3 |
| TX05V7269 | 120 | 2 | 0.2 | 1 | 0.2 | 0.08 | 0.01 | 0.01 | 0.01 | 25 | 12 | 1 | 0.2 |
| TX05A001188 | 120 | 2 | 0.2 | 1 | 0.2 | 0.08 | 0.01 | 0.02 | 0.01 | 28 | 16 | 1 | 0.1 |
| TX07A001279 | 120 | 2 | 0.3 | 1 | 0.3 | 0.08 | 0.01 | 0.01 | 0.01 | 28 | 15 | 2 | 0.4 |
| TX07A001318 | 120 | 2 | 0.2 | 1 | 0.2 | 0.10 | 0.01 | 0.02 | 0.01 | 27 | 14 | 2 | 0.2 |
| TX07A001420 | 120 | 2 | 0.5 | 2 | 0.4 | 0.10 | 0.01 | 0.02 | 0.01 | 30 | 13 | 3 | 0.3 |
| TX06V7266 | 120 | 2 | 0.3 | 1 | 0.4 | 0.09 | 0.01 | 0.01 | 0.01 | 27 | 14 | 2 | 0.1 |
| OK1067071 | 120 | 2 | 0.2 | 1 | 0.3 | 0.08 | 0.01 | 0.02 | 0.01 | 28 | 15 | 2 | 0.2 |
| OK1067274 | 120 | 3 | 0.4 | 2 | 0.3 | 0.10 | 0.01 | 0.02 | 0.01 | 32 | 15 | 3 | 0.5 |
| OK1068002 | 120 | 3 | 0.5 | 2 | 0.2 | 0.10 | 0.01 | 0.02 | 0.01 | 35 | 16 | 4 | 0.4 |
| OK1068009 | 120 | 2 | 0.2 | 1 | 0.2 | 0.10 | 0.01 | 0.02 | 0.01 | 33 | 12 | 2 | 0.2 |
| OK1068026 | 120 | 2 | 0.1 | 1 | 0.3 | 0.08 | 0.01 | 0.01 | 0.01 | 27 | 17 | 2 | 0.2 |
| OK1068112 | 120 | 2 | 0.2 | 1 | 0.2 | 0.08 | 0.01 | 0.01 | 0.01 | 25 | 16 | 1 | 0.1 |
| OK1070275 | 120 | 2 | 0.2 | 1 | 0.2 | 0.08 | 0.01 | 0.01 | 0.01 | 26 | 18 | 1 | 0.1 |
| OK1070267 | 120 | 2 | 0.3 | 2 | 0.5 | 0.10 | 0.01 | 0.02 | 0.01 | 32 | 17 | 3 | 0.3 |
| OK09634 | 120 | 2 | 0.2 | 1 | 0.3 | 0.08 | 0.01 | 0.02 | 0.01 | 30 | 18 | 1 | 0.1 |
| OK10119 | 120 | 2 | 0.1 | 1 | 0.3 | 0.08 | 0.01 | 0.02 | 0.01 | 31 | 18 | 1 | 0.2 |
| GALLAGHER | 120 | 2 | 0.2 | 1 | 0.3 | 0.10 | 0.01 | 0.02 | 0.01 | 36 | 16 | 2 | 0.0 |
| OK07231 | 120 | 2 | 0.5 | 1 | 0.4 | 0.10 | 0.01 | 0.02 | 0.01 | 31 | 18 | 3 | 0.4 |
| OK07S117 | 120 | 3 | 0.6 | 1 | 0.5 | 0.09 | 0.01 | 0.02 | 0.01 | 39 | 23 | 3 | 0.4 |
| OK08328 | 120 | 2 | 0.2 | 1 | 0.3 | 0.09 | 0.01 | 0.02 | 0.01 | 29 | 17 | 2 | 0.1 |
| BIG_SKY | 120 | 2 | 0.2 | 1 | 0.3 | 0.09 | 0.01 | 0.02 | 0.01 | 29 | 14 | 2 | 0.2 |
| DANBY | 120 | 2 | 0.4 | 1 | 0.3 | 0.10 | 0.01 | 0.02 | 0.01 | 30 | 15 | 2 | 0.3 |
| E2041 | 120 | 2 | 0.2 | 1 | 0.2 | 0.09 | 0.01 | 0.01 | 0.01 | 25 | 15 | 1 | 0.1 |
| DENALI | 120 | 2 | 0.5 | 2 | 0.5 | 0.10 | 0.01 | 0.02 | 0.01 | 33 | 13 | 3 | 0.7 |
| CO050337-2 | 120 | 2 | 0.3 | 1 | 0.4 | 0.10 | 0.01 | 0.02 | 0.01 | 30 | 15 | 3 | 0.2 |
| BYRD | 120 | 2 | 0.5 | 2 | 0.4 | 0.10 | 0.01 | 0.02 | 0.01 | 33 | 13 | 3 | 0.3 |
| CO07W245 | 120 | 3 | 0.2 | 2 | 0.2 | 0.10 | 0.01 | 0.02 | 0.01 | 33 | 18 | 4 | 0.3 |
| MCGILL | 120 | 2 | 0.2 | 1 | 0.3 | 0.09 | 0.01 | 0.02 | 0.01 | 31 | 19 | 2 | 0.2 |
| NE02558 | 120 | 2 | 0.3 | 1 | 0.4 | 0.09 | 0.01 | 0.02 | 0.01 | 31 | 19 | 2 | 0.2 |
| NW03666 | 120 | 2 | 0.5 | 1 | 0.5 | 0.10 | 0.01 | 0.02 | 0.01 | 30 | 17 | 2 | 0.5 |
| NE04490 | 120 | 2 | 0.6 | 2 | 0.5 | 0.10 | 0.00 | 0.01 | 0.01 | 27 | 15 | 3 | 0.3 |
| NE05430 | 120 | 2 | 0.1 | 1 | 0.3 | 0.08 | 0.01 | 0.01 | 0.01 | 27 | 17 | 1 | 0.2 |
| NE05496 | 120 | 3 | 0.5 | 2 | 0.5 | 0.09 | 0.01 | 0.02 | 0.01 | 30 | 18 | 4 | 0.3 |
| NE05548 | 120 | 2 | 0.2 | 1 | 0.3 | 0.10 | 0.01 | 0.02 | 0.01 | 29 | 19 | 2 | 0.1 |
| NE06545 | 120 | 2 | 0.2 | 1 | 0.3 | 0.09 | 0.01 | 0.01 | 0.01 | 25 | 18 | 2 | 0.1 |
| NE06607 | 120 | 2 | 0.3 | 1 | 0.4 | 0.09 | 0.01 | 0.02 | 0.01 | 28 | 12 | 2 | 0.2 |
| ROBIDOUX | 120 | 2 | 0.2 | 1 | 0.2 | 0.09 | 0.01 | 0.01 | 0.01 | 25 | 14 | 1 | 0.3 |
| NI06736 | 120 | 2 | 0.2 | 1 | 0.2 | 0.09 | 0.01 | 0.02 | 0.01 | 30 | 17 | 1 | 0.2 |
| NI06737 | 120 | 2 | 0.2 | 1 | 0.3 | 0.10 | 0.01 | 0.02 | 0.01 | 30 | 15 | 2 | 0.2 |
| NI07703 | 120 | 2 | 0.0 | 1 | 0.3 | 0.10 | 0.01 | 0.02 | 0.01 | 31 | 11 | 2 | 0.2 |
| NI08707 | 120 | 3 | 0.3 | 2 | 0.5 | 0.09 | 0.01 | 0.02 | 0.01 | 31 | 16 | 4 | 0.6 |
| NI08708 | 120 | 2 | 0.2 | 1 | 0.3 | 0.10 | 0.01 | 0.02 | 0.01 | 35 | 16 | 2 | 0.2 |
| EVEREST | 120 | 2.6 | 0.6 | 2 | 0.5 | 0.10 | 0.01 | 0.02 | 0.01 | 38 | 14 | 3 | 0.4 |
